# Effect of human urinary microenvironment and fluid flow on antibiotic and phage therapy efficacy against uropathogenic *Escherichia coli*

**DOI:** 10.1038/s41467-026-76589-y

**Published:** 2026-09-04

**Authors:** Ramon Garcia Maset, Aaron Crowther, Victoria Chu, Davide De Grandi, Rizka O. A. Jariah, Jenny Yoon, Dalia Blumgart, Marloes A. E. Hodek, Francesca Torelli, Nicholas Yuen, Benjamin O. Murray, Katie L. Holden, James Bancroft, Laia Pasquina-Lemonche, Sara N. H. Alghamd, Andrew D. Millard, Gareth LuTheryn, Melissa E. K. Haines, Martha R. J. Clokie, Gabriele Pollara, Eleanor Stride, Dario Carugo, Jennifer L. Rohn

**Affiliations:** 1 Centre for Kidney and Bladder Health, Division of Medicine, University College London, London, UK; 2 Institute of Biomedical Engineering, Department of Engineering Science, University of Oxford, Oxford, UK; 3 Nuffield Department of Orthopaedics, Rheumatology and Musculoskeletal Sciences, University of Oxford, Oxford, UK; 4 Becky Meyer Centre for Phage Research, Division of Microbiology and Infection, University of Leicester, Leicester, UK; 5 Medical Laboratory Technology, Faculty of Vocational, Airlangga University, Surabaya, Indonesia; 6 Cellular Imaging Core Facility, Nuffield Department of Medicine, University of Oxford, Oxford, UK; 7 School of Biosciences, University of Sheffield, Sheffield, UK; 8 Institute of Infection, Immunity and Transplantation, University College London, London, UK

**Keywords:** Antimicrobials, Cellular microbiology

## Abstract

Urinary tract infection (UTI) remains a major global health burden, with frequent recurrences and rising antimicrobial resistance compromising treatment efficacy. Conventional susceptibility assays often fail to predict clinical outcomes, highlighting the need for more physiologically relevant infection models. Here, we investigated how microenvironmental complexity influences uropathogenic *Escherichia coli* (UPEC) responses to antibiotics and bacteriophages using human urine, a three-dimensional human urothelial microtissue model (3D-UHU), and a novel mesofluidic platform (P-FLO) that applies physiologically relevant wall shear stress to 3D-UHU. Nitrofurantoin showed the greatest potency in conventional susceptibility assays but did not eradicate infection in the static 3D-UHU model. A bacteriophage cocktail (LCPR1) inhibited intracellular bacterial communities, preserved urothelial viability and induced inflammatory cytokine and chemokine secretion. Combined LCPR1 + nitrofurantoin treatment eliminated planktonic bacteria and reduced extracellular and intracellular bacterial communities under static conditions but did not further reduce urothelium-associated bacterial burden compared with nitrofurantoin alone. Under flow, shear stress promoted bacterial elongation, attachment and intracellular community formation, while reducing nitrofurantoin and combination therapy efficacy despite increased drug exposure. These findings demonstrate that the bladder microenvironment profoundly influences UPEC infection dynamics and therapeutic outcomes, underscoring the need for advanced models to guide treatment strategies in the antibiotic resistance era.

## Abstract (web summary)

Maset et al. show that bladder conditions strongly influence how urinary tract infections respond to antibiotics and bacteriophages, highlighting the need for more realistic lab models to improve diagnosis and treatment of recurrent infections.

## Introduction

With ~400 million cases per year, urinary tract infections (UTI) are among the most prevalent human infections, disproportionately affecting women and contributing significantly to the global burden of the antimicrobial resistance (AMR) crisis (with ~260,000 AMR-associated UTI deaths annually)^1,2^. Approximately 80% of community-acquired UTIs are caused by uropathogenic *E. coli* (UPEC), a species also linked to the highest number of deaths associated with AMR^3–5^. Although UTI is often regarded as readily treatable, its high recurrence rate belies this view, with the biological and ecological processes governing infection, clearance and treatment failure only partly understood^6^.

The urothelium is a highly dynamic environment, shaped by fluctuating osmolarity, pH, nutrient composition, urine flow, and stretching cycles during the filling and voiding phases^7^. These factors influence bacterial physiology (adhesion, filamentation, invasion of uroepithelial cells, and biofilm formation), while host-derived components such as urea concentration, antimicrobial peptides, immune cell infiltration and the resident urinary microbiota further modulate pathogen physiology and antibiotic susceptibility^8–11^. Together, the urinary tract microenvironment creates a complex niche that modulates bacterial behaviour and influences the host/pathogen interaction alongside treatment efficacy.

Despite the availability of effective antibiotics, recurrence and relapse are common (up to 30% of cases), and repeated antimicrobial exposure contributes to the escalating global problem of AMR^12,13^. Treatment failure in UTI often occurs even when pathogens appear to be susceptible to conventional drugs. Standard in vitro susceptibility assays are typically conducted under nutrient-rich and static conditions that fail to recapitulate the physicochemical and fluid mechanical complexity of the urinary tract^14^. As a result, antimicrobial efficacy measured by minimum inhibitory concentration (MIC) can correlate poorly with clinical outcomes^15,16^. Understanding how physiological conditions modulate antibiotic activity is essential for refining clinical breakpoints and optimising dosing strategies for UTI^17^.

Existing experimental models of UTI (ranging from cell culture to animal infection systems) have provided important mechanistic insights into UPEC behaviour but exhibit significant translational limitations. Murine models, for example, differ from humans in terms of bladder physiology, immune response and urine composition^18^. Furthermore, pharmacokinetic and pharmacodynamic parameters of antibiotics often translate poorly between mice and humans, hindering efforts to define optimal treatment regimens^19^.

Recent tissue-engineering advances have provided human-relevant models to study UTI pathology. For example, a donor-derived human bladder organoid model has been developed to investigate UPEC and bacteriophage interaction at the molecular level^20^. To modulate stretching patterns during micturition and immune cell infiltration, a bladder-on-chip model was established to investigate intracellular reservoirs^21^. Advanced mini-bladder microphysiological systems incorporating stratified urothelium, urine perfusion and micturition-like dynamics have further demonstrated how urine composition can reduce fosfomycin efficacy and promote persistent cell wall-deficient UPEC populations^22^. We recently developed the static 3-dimensional urine-tolerant human urothelial microtissue Transwell model (3D-UHU), which allows investigation of UTI in a fully stratified and differentiated urothelium in a 100% human urine apical environment^23,24^. Together, these innovations and others are driving a new era of personalized, mechanism-based approaches to investigate UTI and antimicrobial therapies.

Treatment failure in UTI underscores the urgent need to explore alternative or adjunctive therapeutic approaches that can overcome bacterial adaptation^4,14^. Increasing evidence suggests that non-genetic drivers including phenotypic tolerance and persistence, intracellular reservoirs and biofilm formation enable UPEC and other uropathogens to withstand otherwise effective antibiotic therapy. Innovative strategies that exploit different antibacterial mechanisms or synergise with existing antibiotics are gaining renewed attention. Bacteriophage (phage) therapy, either alone or in combination with antibiotics, offers a promising avenue to address these challenges^25^. Phages exhibit high specificity, self-amplifying dynamics and, in some cases, the capacity to disrupt biofilms and target metabolically quiescent bacterial populations^25^. Recent studies demonstrate that phage-antibiotic combinations can enhance bacterial clearance, suppress resistance evolution, and restore antibiotic susceptibility in vitro and in animal models^26^. Yet how such interventions perform within the complex urinary microenvironment and how host factors, such as flow dynamics and urine composition, influence phage activity remain largely unexplored.

Here, we investigated the antimicrobial efficacy of nitrofurantoin, an antibiotic commonly used to treat UTI, against UPEC in increasingly complex environments. These include human urine; the 3D-UHU human urothelial static microtissue model; and the Plug-Flow Linked Organoid (P-FLO) system, a novel mesofluidic (i.e., operating in the millimetre scale) version of 3D-UHU developed from inexpensive and simple 3D-printed components, allowing us to adopt commercially available Transwells to create a more physiologically relevant urine flow dynamic environment. We also used the static model as well as P-FLO to assess the activity of bacteriophages alone and in combination with antibiotics. These experiments demonstrated the marked effect of microenvironment on the response of UPEC to infection and treatment.

## Results

### Antimicrobial susceptibility testing (AST) against UPEC strains in caMHB vs human urine

**Fig. 1:**
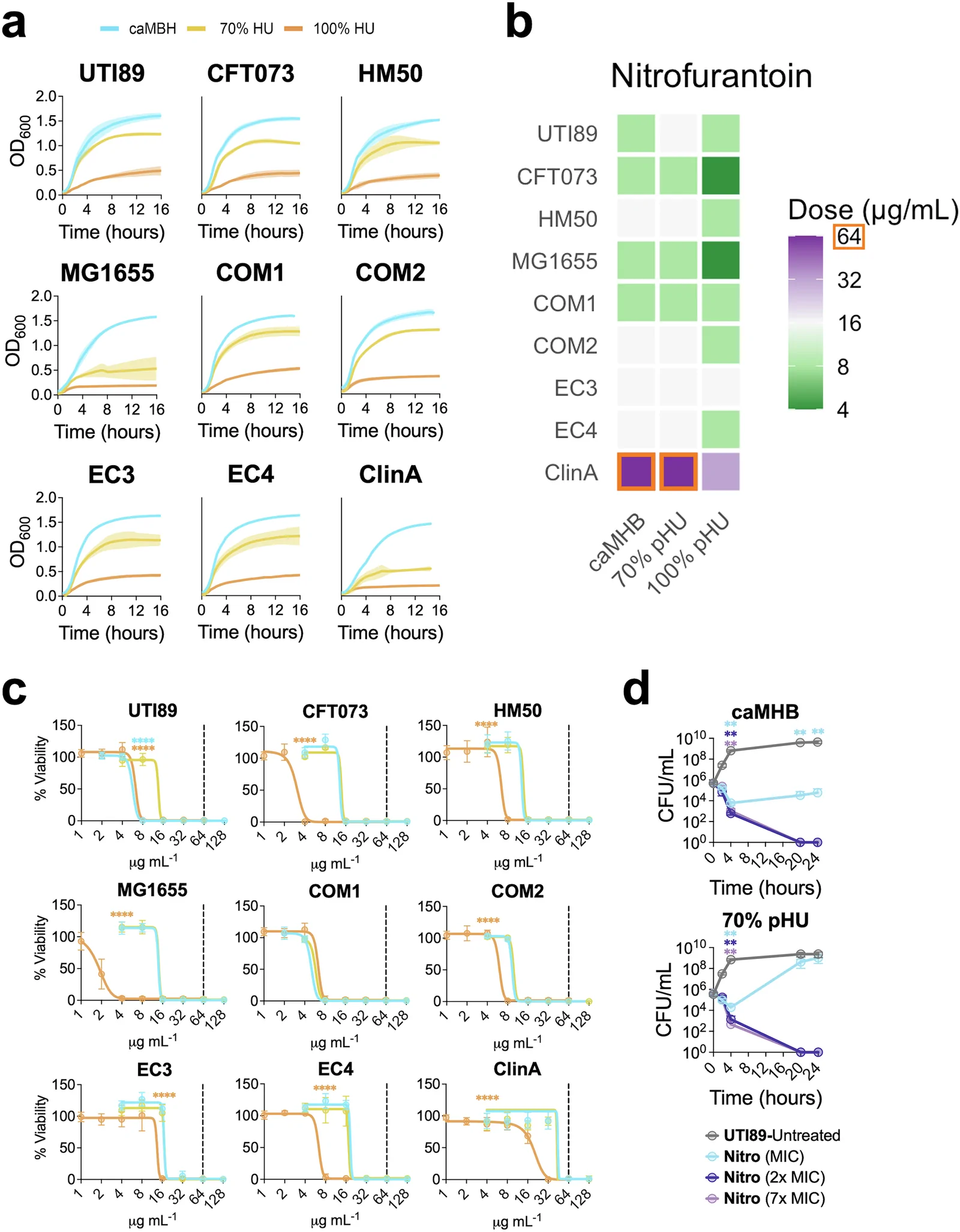
Urine-containing media can affect the growth and antibiotic sensitivity of *E. coli*. **a** Growth curves of *E. coli* strains in caMHB (blue), 70% pHU (yellow) and 100% pHU (orange). Lines represent mean values and shaded regions indicate ± standard deviation (s.d.) derived from *n* = 3 independent biological experiments. Each biological replicate consisted of an independently cultured bacterial population initiated from a separate overnight culture and represents the unit of study. For each biological replicate, measurements were performed in three technical replicates. **b** Heat map of the MIC results of nitrofurantoin against *E. coli* strains in caMHB, 70% pHU and 100% pHU (*n* = 3 independent biological experiments). The orange square border defines a MIC value at the EUCAST breakpoint. **c** Dose-response graphs of *E. coli* strains in caMHB (blue), 70% pHU (yellow) and 100% pHU (orange) against nitrofurantoin. The dashed black line defines the EUCAST breakpoint. A two-sided *t*-test was performed to compare viability at each concentration (*blue indicates comparisons between 70% pHU and caMHB; *orange indicates comparisons between 100% pHU and 70% pHU). The circles present mean values ± s.d. from *n* = 3 independent biological experiments defined as in (**a**). For each biological replicate, measurements were performed in three technical replicates. **d** Time killing assay of *E. coli* UTI89 against nitrofurantoin in caMHB (top) and in 70% pHU (bottom). A two-sided *t*-test was performed to compare CFU counts of each treatment with the untreated control. The circles present mean value ± s.d. from *n* = 4 independent biological experiments defined as above. Statistical significance was defined as none (ns), *p* ≥ 0.05; *p < 0.05; **p < 0.01; ***p < 0.001; and ****p < 0.0001.

Antibiotic efficacy is still primarily assessed using a single in vitro bioassay in standard growth medium, namely the minimum inhibitory concentration (MIC) assay^27^. However, this assay overlooks key environmental factors present during host-pathogen interactions in vivo that profoundly influence drug performance^28^. We therefore evaluated whether the addition of urine into the MIC assay might alter the sensitivity patterns observed. First, we assessed bacterial growth in various media. In addition to the gold-standard growth media (caMHB) recommended by the European Committee on Antimicrobial Susceptibility Testing (EUCAST) guidelines for MIC, we included 70% pooled human urine (pHU) (in 30% caMHB) to mimic a UTI infection microenvironment. Such a 70% urine mix was previously used to determine drug susceptibility while still supporting adequate bacterial growth^29^. Growth in 100% pooled human urine (pHU) was markedly impaired (Fig. 1a), with prolonged doubling times and substantially reduced maximal growth relative to 70% pHU (Supplementary Fig. 1a). Although bacterial growth in 70% pHU remained lower than in caMHB, consistent with the reduced nutrient availability of urine-based media, growth kinetics more closely resembled those observed in caMHB (Fig. 1a), including comparable doubling times and area under the curve (AUC), with strain-dependent differences (Supplementary Fig. 1a). Together, these results indicated that 70% pHU provided a more suitable balance between physiological relevance and bacterial growth support for subsequent antimicrobial assays.

Next, we investigated the AST profiles of nitrofurantoin, one of the first-line antibiotics recommended for acute UTI in the UK and many other countries^30^, against nine *E. coli* strains including clinical UPEC isolates (EC3, EC4, ClinA), non-symptomatic strains (HM50, COM1, COM2), and reference strains such as the commonly studied pyelonephritis strain (CFT073) and cystitis strain (UTI89), alongside a non-UPEC laboratory strain (MG1655). As shown in Fig. 1b, the growth media had minimal effect on nitrofurantoin activity, and differences were observed between strains. All but one of the strains (ClinA) tested showed MICs ranging from 4 to 16 μg/mL. The lowest MICs were reported in 100% urine, which may reflect technical difficulties in visualising endpoints when bacterial growth is not robust, as is the case under this condition. However, there were no differences in nitrofurantoin MIC between caMHB and 70% pHU.

We next tested other antibiotics commonly used to treat UTIs^31^ (Supplementary Fig. 1b). Trimethoprim was active against all strains except EC3 and EC4, with MICs above the breakpoint in both caMHB and 70% pHU. For fosfomycin, amoxicillin and cefalexin, 5 of the 9 strains exhibited MICs above the breakpoints comparably in both microenvironments tested. Overall, despite exceptions such as EC3, EC4 and ClinA showing higher MICs for cefalexin in 70% pHU compared to caMHB, most antibiotics showed comparable antimicrobial activity in 70% pHU as in caMHB, indicating that the presence of urine can affect AST depending on the strain and antibiotic tested, but that these effects are not substantial in an in vitro environment, and are likely not sufficient to explain the lack of concordance between AST results and clinical outcomes.

Next, we determined the minimum bactericidal concentration (MBC) of all the above drugs and strains by measuring bacterial viability as shown in Fig. 1c and Supplementary Fig. 1c–f. In general, we again observed lower MBC levels in the 100% urine condition due to the reduced growth conditions. However, in the more growth-amenable media (caMHB vs 70% pHU), the MBC values for nitrofurantoin were identical for all the strains except UTI89, which showed only a 1-fold increase in the 70% urine microenvironment. This result generally applied to the other drugs tested, with either identical MBCs for both media or a one-fold increase in MBCs for 70% pHU medium. Similar to the MIC results, the urine microenvironment had modest strain- and antibiotic-dependent effects on MBCs that are also unlikely to explain the discordance with clinical outcomes.

Nitrofurantoin remains a resilient and popular therapeutic option for UTIs, due to its localised delivery, favourable pharmacokinetic and pharmacodynamic properties, and multifaceted effects on bacterial physiology^32,33^. Resistance acquisition is constrained by the need for multiple independent mutations and the near absence of horizontally transferable resistance mechanisms^34^. Therefore, we focused on nitrofurantoin for all subsequent steps of this study. In addition, we narrowed our efforts to strain UTI89, as it is one of the most characterised UPEC isolates.

Accordingly, we investigated further the killing kinetics of nitrofurantoin against *E. coli* UTI89, comparing caMHB and 70% pHU microenvironments (Fig. 1d), which showed distinct media-specific profiles. In caMHB, nitrofurantoin at MIC, 2xMIC, and 7xMIC for 4 h caused a significant CFU reduction in comparison with the untreated control (all *p* < 0.001). At 20 h and 24 h, nitrofurantoin at MIC significantly reduced CFUs in comparison with the untreated control (all *p* < 0.001), while 2xMIC and 7xMIC caused total bacterial eradication. In 70% pHU, nitrofurantoin at MIC, 2xMIC and 7xMIC at 4 h caused a statistically significant CFU reduction in comparison with the untreated control (all *p*  < 0.001). At 20 h and 24 h, nitrofurantoin at MIC was not significantly different to the untreated control, while 2xMIC and 7xMIC caused total bacterial eradication. In both microenvironments, at least 2xMIC was needed to eradicate the bacteria. However, in the case of 70% pHU, a higher dose of nitrofurantoin was necessary to achieve bacterial eradication (4-fold increase in drug concentration) in comparison with caMHB. Taken together, these results suggest a modest, but important, effect of urine (one key component of the urinary microenvironment) on nitrofurantoin response, in the in vitro conditions evaluated.

### Effect of nitrofurantoin against *E. coli* UTI89 in a human bladder microtissue model under static (no flow) conditions

**Fig. 2:**
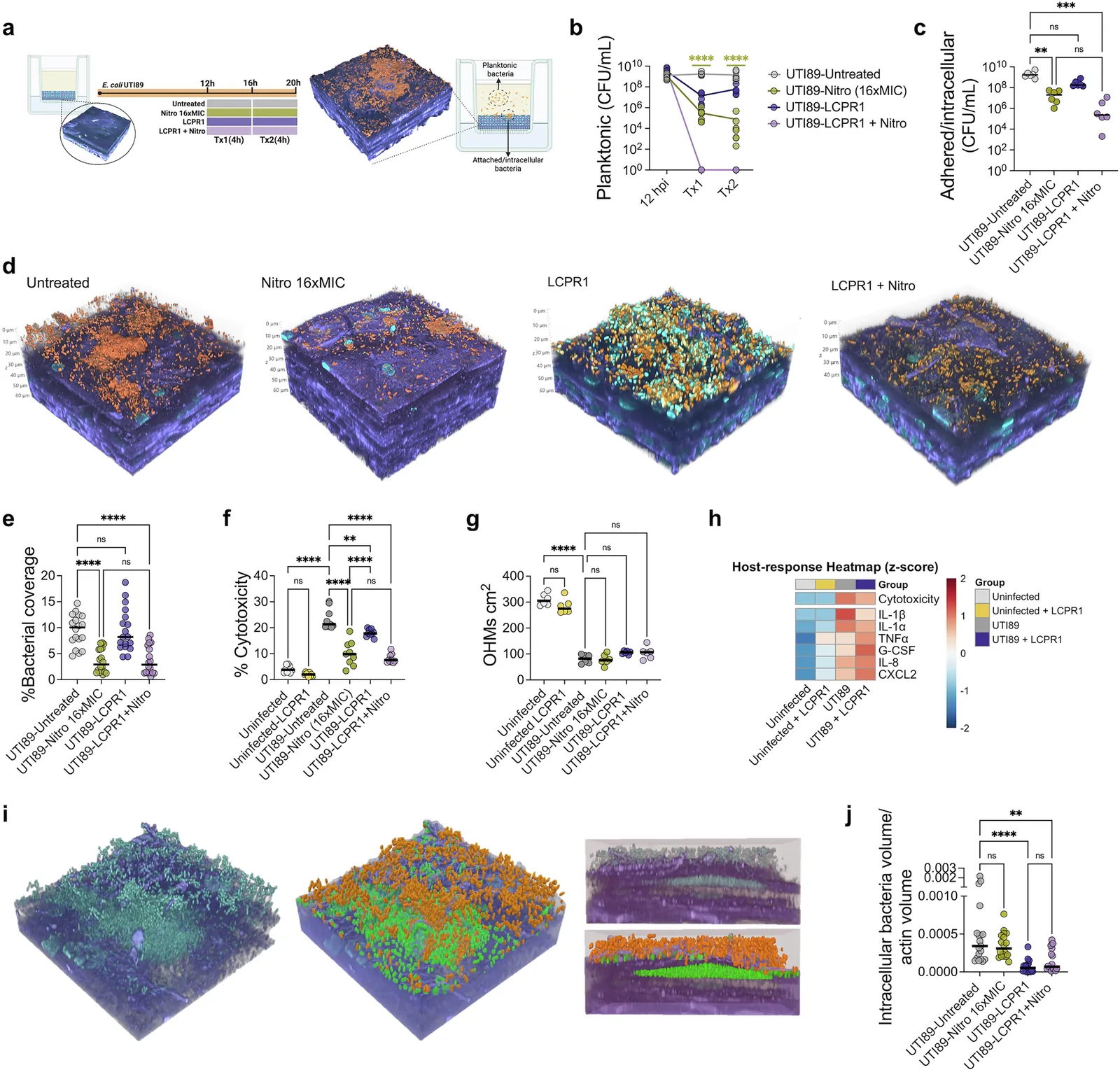
The 3D-UHU urothelial enviroment affects host-*E. coli* interactions and the response to phage and antibiotic treatment. **a** Experimental set-up. Created in BioRender. Garcia Maset, R. (2026) https://BioRender.com/jec2lo0. **b** Bacterial load in apical side (planktonic) after 12 hpi, post-4h 1st treatment (Tx1) and post-4h 2nd treatment (Tx2). Kruskal-Wallis two-sided test compared Tx1 and Tx2 with untreated. **c** Bacterial load of attached/intracellular bacteria after Tx1 and Tx2, Kruskal-Wallis test as above. For (**b–c**), each circle represents one microtissue, *n* = 3 independent biological experiments using distinct cell passage and independent bacterial culture; two technical replicates (microtissues derived from same cell passage and infected with the same culture) were included. **d** 3D confocal micrographs of *E. coli* UTI89-GFP (orange) treated as shown, focused on regions with bacterial attachment due to sample heterogeneity; DNA (cyan), F-actin (purple). **e** Percentage bacterial area coverage of urothelial surface from image analysis. *N* = 3 independent biological experiments as defined above. Each circle represents six images acquired/analysed per biological experiment as technical replicates. Horizontal lines indicate mean values. Ordinary one-way ANOVA (two-sided) compared groups. **f** Cytotoxicity (LDH release) 20 hpi. Each circle represents one microtissue, *n* = 3 independent biological experiments and 3 technical replicates as defined above. Ordinary one-way ANOVA as for (**e**). **g** 3D-UHU barrier integrity (TEER) 20 hpi, replicates/statistics as for (**f**). **h** Host-response heatmap from 3D-UHU supernatant 20 hpi; replicates as for (**b–c**). **i** Intracellular bacteria quantification using image analysis. 3D rendering of untreated microtissue (left), superimposed 3D render mask-segmentation (middle) to distinguish extracellular bacteria (orange) from intracellular (green), and cross-section in xy axis (right top) and with segmentation rendering (bottom) to illustrate an IBC (green). **j** Quantification of IBC in models post-infection and -antimicrobial treatment. Data are *n* = 3 biological experiments defined as above. Each circle represents seven images acquired/analysed per biological experiment as technical replicates. Horizontal lines indicate mean values. An ordinary Anova test (two-sided) compared the different groups. For all graphs, statistical significance was defined as none (ns), *p* ≥ 0.05; *p < 0.05; **p < 0.01; ***p < 0.001; and ****p < 0.0001.

To explore additional aspects of the urinary microenvironment on UPEC growth, we used our urine-tolerant human urothelial (3D-UHU) microtissue model, a more physiologically complex setting that recapitulates key features of host-pathogen interactions, as a novel platform for antimicrobial treatment screening^7,23^. Using the experimental setup outlined in Fig. 2a, the urothelium was infected with *E. coli* UTI89 for 12 h, then infected tissues were treated with two consecutive doses of antimicrobial agents in the apical urine compartment (Tx1 and Tx2), each administered for 4 h.

Firstly, we determined the efficacy of nitrofurantoin at a clinically relevant concentration, consistent with reported human urinary pharmacokinetics^34^ (16xMIC, 256 μg mL^−1^). Treatment significantly reduced planktonic bacterial burden in the apical compartment, with a ~3-4 log CFU reduction observed following both first (Tx1) and second (Tx2) treatment exposures compared with the untreated control (Fig. 2b; *p* < 0.0001). In contrast, nitrofurantoin treatment against urothelium-associated bacteria, which included attached and intracellular bacterial populations, was less effective, only manifesting a ~2-log reduction (Fig. 2c; *p* = 0.0059); this finding was consistent with reductions observed in the confocal images (Fig. 2d) and the subsequent bacterial coverage area analysis (Fig. 2e and Supplementary Fig. 2a) using images acquired at 40x magnification, enabling analysis of a larger microtissue area and providing a more representative assessment of bacterial distribution. Additionally, nitrofurantoin reduced epithelial cytotoxicity, as evidenced by reduced LDH release (*p* < 0.0001) relative to untreated controls (Fig. 2f). However, the barrier function of the microtissue model, as measured by TEER, was already compromised after 12 h of infection (Supplementary Fig. 2b), which could not be restored by the nitrofurantoin treatment (Fig. 2g).

The antimicrobial effect of nitrofurantoin against UTI89 in the 3D-UHU was dose-dependent. At 7xMIC (112 μg mL^−1^), in the planktonic population, a significant CFU reduction (*p* < 0.001) was observed after the Tx1, whilst bacterial re-growth was observed after Tx2 (*p* = 0.494) (Supplementary Fig. 2c). No significant difference in bacterial burden was observed in the attached/intracellular bacterial populations (*p* = 0.7739) (Supplementary Fig. 2d). Post-infection, the bacterial population exposed to the bladder microtissue environment (challenged with 7xMIC nitrofurantoin or untreated) was recovered and exposed to nitrofurantoin using a MIC microdilution method with standard growth medium. The MIC remained unchanged, and both groups exhibited an identical MIC and MBC before and after the exposure to the bladder microenvironment (Supplementary Fig. 2e), confirming that antimicrobial resistance mutations had not occurred during the course of the experiment. Despite this confirmed in vitro drug sensitivity, treatment at concentrations above the MIC (7xMIC) failed to reduce the bacterial burden in the urothelium (Supplementary Fig. 2c, d), exemplifying the discrepancy between simplified in vitro tests and platforms that mimic the host microenvironment and host-pathogen interactions^14^.

### Phage cocktail (LCPR1) and nitrofurantoin combinatorial therapy against *E. coli* UTI89 in a human bladder microtissue model under static (no flow) conditions

Following evaluation of conventional antibiotic therapy, we next investigated the potential of bacteriophage-based therapy to target *E. coli* UTI89. The bacteriophage cocktail (LCPR1) was previously optimised in a separate project and comprises three *E. coli* phages and six *K. pneumonia* phages. It was developed using a broad panel of antibiotic-resistant urinary clinical isolates collected within the UK. The inclusion of multiple phages is a known strategy to broaden the host range and improve the phage cocktail coverage^35–37^.

The growth kinetics of UTI89 were monitored under exposure to LCPR1 using different MOI (phage to bacteria) in two different media (MHB and 70% pHU:30% MHB). 100% pHU was not included because UTI89 exhibited insufficient growth in urine alone (Supplementary Fig. 1a) to support reliable assessment of phage replication and antibacterial activity. The bacterial growth was impaired up to 10 h for all MOIs tested in MHB. After this period, partial regrowth was observed (OD_600_ ~ 0.5), while the untreated control reached full growth (OD_600_ ~ 2) (Supplementary Fig. 3a). In contrast, in 70% pHU:30% MHB, the UTI89 growth curve in the presence of LCPR1 (MOI = 1 and 10) exhibited a similar growth pattern in comparison with the untreated control. Only at MOI 100, a more pronounced effect in growth was observed (Supplementary Fig. 3b). At MOI 100, LCPR1 decreased UTI89 growth in both media versus the untreated UTI89 control, but the area under the curve (AUC) remained higher in 70% pHU:30% MHB than in standard medium, consistent with reduced phage efficacy in urine (Supplementary Fig. 3c). Despite differences in initial killing efficacy, after 24 h, LCPR1 at MOI 100 reduced bacterial CFU by ~4 log in MHB and ~3 log in 70% pHU:30% MHB pHU (Supplementary Fig. 3d). Phage quantification following the killing assay demonstrated varying amplification levels after 24 h for each *E. coli* phage (Leic_001, JK03, and UP17). A reduction of phage titres (uninfected control with LCPR1 and media alone) in 70% pHU:30%MHB titre was also observed for Leic_001 (*p* < 0.0001) and UP17 (*p* = 0.0135) in comparison with MHB, suggesting that phages were less stable in the urine microenvironment media (Supplementary Fig. 3e–g). No differences in phage titre were observed for JK03 in both media. In the UTI89 groups co-infected with LCPR1, the titre of Leic_001 was higher in 70% pHU:30%MHB than in MHB (*p* < 0.0001), whereas this trend was not observed for the other two *E. coli* phages (Supplementary Fig. 3e). The titres of JK03 and UP17 were comparable between the two media (Supplementary Fig. 3f, g). These findings underscore the influence of the type of culture medium on phage activity, suggesting that host-like conditions may limit therapeutic efficacy compared with standard laboratory media, as previously reported by others^38,39^.

Next, we evaluated the effect of LCPR1 (1 × 10^9^ phage mL^−1^) against *E. coli* UTI89 using the static 3D-UHU microtissue infection model (Fig. 2a). LCPR1 failed to significantly reduce bacterial burden (*p* = 0.0971) and colonisation of the urothelium (*p* = 0.8377) in comparison with the untreated control (Fig. 2b–e and Supplementary Fig. 2a). Notably, LCPR1 alone attenuated cellular cytotoxicity induced by UTI89 infection (*p* = 0.001), but this effect was modest relative to the protective effect on cell viability of nitrofurantoin alone (*p* < 0.0001) (Fig. 2f). To better assess the effect of LCPR1 on urothelial function, we also measured secretion of several inflammatory cytokines and chemokines. The addition of LCPR1 alone induced modest secretion of the pro-inflammatory cytokine TNFα, alongside the chemokines CXCL2 and G-CSF, known to promote neutrophil tissue residency^40^. UTI89 infection induced the secretion of pro-inflammatory cytokines and chemokines, although not CXCL1 (Supplementary Fig. 4). Strikingly, LCPR1 co-infection with UTI89 greatly reduced IL-1β secretion, mirroring the effects seen with cytotoxicity. This was in sharp contrast to the effects on secretion of G-CSF, the neutrophil chemoattractants CXCL2 and IL-8 (CXCL8), and the pro-inflammatory cytokine TNFα, all of which showed greater UTI89-induced secretion following LCPR1 co-infection (Fig. 2h and Supplementary Fig. 4). Together, these findings suggest that while LCPR1 lacks bactericidal efficacy in 3D-UHU, it may protect urothelial viability, attenuate inflammasome activation and promote neutrophil influx in the urothelial environment.

We next assessed the impact of combining nitrofurantoin and LCPR1 on bacterial clearance and host tissue damage. The dual treatment caused total bacterial eradication in the apical compartment and achieved a ~4-log CFU reduction (*p* < 0.0001) in the microtissue compartment in comparison with the untreated control; a level of reduction considered clinically relevant^41^ (Fig. 2b, c). The reduction in bacterial coverage was similar to that observed with nitrofurantoin alone (Fig. 2d, e and Supplementary Fig. 2a). Nitrofurantoin alone attenuated cytotoxic effects of UTI89 infection, and there was no additional reduction in cytotoxicity by LCPR1 (*p* = 0.6663) (Fig. 2f).

Phage recovery from the 3D-UHU was also assessed in both the apical (Tx1 and Tx2) and the microtissue compartments to evaluate phage dynamics and replication (Supplementary Fig. 5a–c). In the apical planktonic compartments, phage recovery varied among the individual phages. JK03 showed the lowest titre under the combined treatment in both Tx1 and Tx2 (Supplementary Fig. 5a, b), whereas comparable titres were observed for Leic_001 and UP17 in Tx2 (Supplementary Fig. 5b). In the microtissue compartment (adhered/intracellular), all three phages recovered from LCPR1 alone had higher titres compared with the dual therapy group (Supplementary Fig. 5c). This finding correlated with the bacterial count result, which showed that the combined treatment more effectively reduced bacterial population compared with LCPR1 alone (Fig. 2b, c), thereby limiting phage amplification due to reduced host availability. Nevertheless, this pattern was not observed in the apical phage recovery results, which suggests bacteria-dependent phage uptake or replication within the microtissue compartment, highlighting the complexity of phage-bacteria dynamics within the bladder microenvironment.

### Effect of antimicrobial treatment on intracellular bacterial communities (IBCs)

One possible cause for treatment failure in UTI is the fact that uropathogens such as UPEC can invade the urothelium and form intracellular bacterial communities (IBCs), which are impervious to antibiotics and to the effect of extracellular immune effectors^42^. Although the gentamicin protection assay is widely used to distinguish intracellular from extracellular bacteria, especially in phagocytic cells^43^, its application in infected epithelial cells and tissues is complicated by the fact that gentamicin works poorly against biofilms and may also struggle accessing dense, adhered bacterial clusters, both of which are known to form on the surface of infected 3D-UHU^23^, potentially leading to incomplete clearance of extracellular bacteria. To overcome this challenge, we developed an image analysis pipeline to directly quantify intracellular and extracellular bacterial populations in our 3D-UHU model. Segmentation algorithms implemented in Arivis were used to resolve DAPI-labelled structures (bacterial cells and host nuclei) and the actin-labelled cytoskeleton, followed by three-dimensional reconstruction. Bacterial objects fully enclosed within the actin-defined cellular mask were classified as intracellular communities (Fig. 2i). As an extra validation step for this pipeline, we also confirmed the presence of IBCs with an antibody against *E. coli* deployed on non-permeabilised microtissue to distinguish intracellular from extracellular bacteria, and with an RFP-tagged UTI89 strain carrying chromosomally integrated RFP to distinguish bacterial DAPI signals from fragmented nuclei or condensed mitotic nuclei (Supplementary Fig. 6). Although nitrofurantoin reduced CFU in the adhered/intracellular compartment (Fig. 2c), it did not impair IBC formation compared with the untreated control (Fig. 2j), suggesting limited efficacy against established intracellular communities. In contrast, LCPR1 treatment reduced IBCs despite having no measurable effect on CFU in the adhered/intracellular compartment (Fig. 2c, j), indicating a selective impact on IBC formation rather than overall bacterial burden. Notably, the combined treatment of phage and antibiotic decreased both the adhered and intracellular bacterial load (Fig. 2c, j), consistent with additive effects of both treatments. Together, these findings reveal complementary activities of nitrofurantoin and LCPR1 against extracellular and intracellular UPEC reservoirs within the urothelium.

### Fluid flow triggers UPEC elongation in a human bladder microtissue model

To approximate the mechanical shear forces generated by urine flow, we engineered a mesofluidic platform called the Plug-Flow Linked Organoid (P-FLO) system (Supplementary Fig. 7a), designed to introduce relevant levels and temporal profiles of wall shear stress into the 3D-UHU microtissue model^23,24^ (and indeed to any other microtissue or planar organoid model compatible with the Transwell system). The system enables cyclic changes of flow rate (and hence of applied wall shear stress) in the apical surface of the differentiated urothelial microtissue, resembling the shear forces associated with the filling and voiding phases of the micturition cycle (Supplementary Fig. 7a). For brevity, we will hereafter refer to these P-FLO conditions as “filling” and “voiding” cycles. A continuous flow of 0.02 mL min^−1^ was applied for 6 h to approximate the shear forces of bladder filling. To reflect urination cycles, this flow was interrupted every 2 h by a 1-min voiding event, namely, the application of an increased flow rate of 0.7 mL min^−1^. Additionally, we tested a flow protocol without the voiding phases (in which a continuous flow of 0.02 mL min^−1^ was applied for 6 h).

This novel experimental setup approximates a key mechanical cue of the bladder environment, enabling assessment of infection progression under conditions that echo in vivo wall shear stress levels as well as the dynamic nature (i.e., alternating low and high wall shear stress) of the micturition cycle. Numerical simulations were performed to investigate the shear stress field generated on the urothelium at the corresponding volumetric flow rates tested. The computational modelling results revealed that the area of highest shear stress (HSS) was near the inlet during the voiding phase, with intermediate shear stress (ISS) levels near the outlet region. During the filling phase, the wall shear stress field was more uniformly distributed and exhibited the lowest shear stress (LSS) values among all simulated conditions (Supplementary Fig. 7b). Notably, application of flow (voiding/filling cycles for 6 h) to uninfected 3D-UHU models did not alter barrier integrity, microtissue thickness or expression of the key differentiation markers CK20 and UPIII, compared with static controls (Supplementary Fig. 7c–f).

**Fig. 3:**
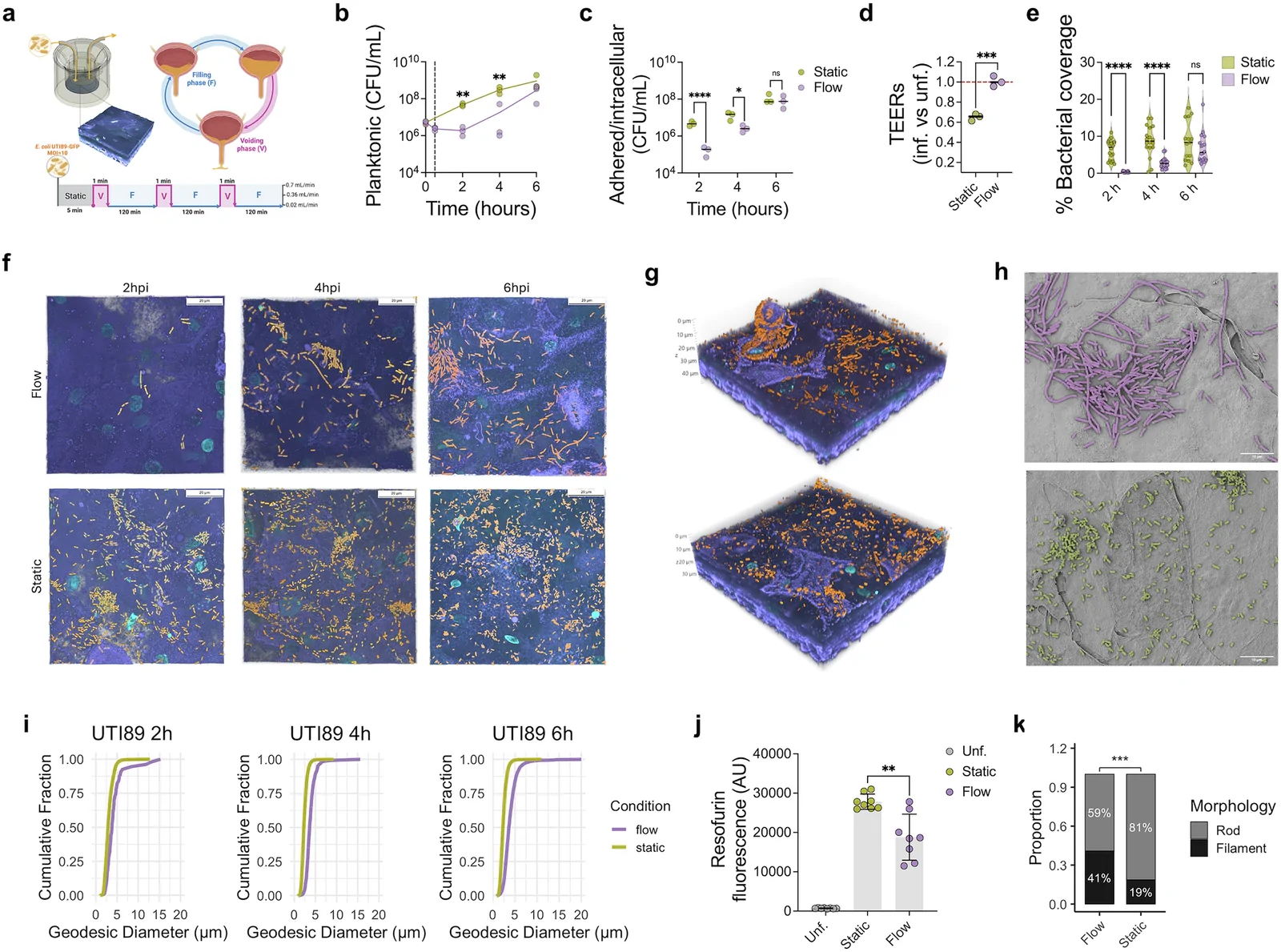
Fluid flow influences host-pathogen interactions and morphology during *E. coli* infection of 3D-UHU. **a** Experimental set-up. Created in BioRender. Garcia Maset, R. (2026) https://BioRender.com/hod2r3. **b** Apical (planktonic) and **c** attached/intracellular *E. coli* UTI89-GFP (*t* = 0, after each void, and *t* = 6 h) in static (no flow) and dynamic (flow) conditions. Data were log-transformed to fit normality and Anova (two sided) compared groups. **d** 3D-UHU barrier integrity (TEER) 6 hpi comparing static vs dynamic conditions; ratio between uninfected and infected. Red dashed line (ratio of 1) indicates uncompromised barrier; values < 1 are compromised. Each circle represents *n* = 3 independent biological experiments, each independently derived from different cell passage; Anova two-sided test as in (**c**). **e** Bacterial coverage area over the urothelial surface from confocal imaging. *N* = 3 biological experiments as defined in (**d**). Each circle represents 6 images acquired/analysed per biological experiment as technical replicates. Horizontal lines indicate mean values; Anova two-sided test as in (**d**). **f** Representative top-down-view confocal micrographs of *E. coli* UTI89-GFP (orange) with indicated conditions; DNA (cyan), F-actin (purple); bar = 20 μm. **g** 3D rendering of UTI89-GFP-infected model with indicated conditions, colours as in (**f**). **h** False-coloured SEM micrographs of UTI89-GFP 6 hpi in dynamic (top) vs static (bottom) conditions; bar=10 μm. **i** Cumulative fraction plot of bacterial length under static (green) vs dynamic (purple) conditions from 3D cell segmentation analysis at 2, 4 and 6 hpi, average cumulative fraction from *n* = 3 biological experiments as defined above, with 3 images acquired/analysed as technical replicates. **j** Metabolic activity (resazurin reduction) of UTI89 from apical (planktonic) compartment 6hpi under dynamic vs. static conditions. Bars represent mean ± standard deviation (s.d.) from *n* = 4 biological experiments as defined above, with two technical replicates (same cell passage and same inoculum). A Welch’s *t-*test (two-sided) compared dynamic vs static. **k** Length analysis of apical UTI89-GFP 6hpi under dynamic vs static conditions. Rods classified as < 6 μm and filaments > 6 μm. Rod/filament proportion in dynamic vs static were compared with a Wilcoxon test; *n* = 3 biological experiments as defined above; 10 images were acquired/analysed per biological experiment as technical replicates. For all graphs, statistical significance was defined as none (ns), *p* ≥ 0.05; *p < 0.05; **p < 0.01; ***p < 0.001; and ****p < 0.0001.

To investigate the impact of fluid dynamics on UPEC infection, 3D-UHU models were infected with *E. coli* UTI89-GFP (Fig. 3a). Exposure to the filling phase alone (continuous flow of 0.02 mL min^−1^ for 6 h), which is associated with the lowest wall shear stress (LSS), resulted in significantly increased bacterial adhesion compared with samples subjected to filling-voiding cycles (*p* < 0.0001; Supplementary Fig. S8a, b). Consistent with previous studies in endothelial cells^44^, our findings suggest that shear stress can enhance UPEC adhesion in the urothelium. However, during cycles reflecting voiding/filling, associated with higher wall shear stress profiles, a reduction of bacterial attachment to the apical surface was observed (Supplementary Fig. 8a). To further assess whether different levels of shear stress reminiscent of voiding differentially affected bacterial adhesion, image analysis was performed in regions exposed to either high (HSS) or intermediate (ISS) wall shear stress. Comparable levels of bacterial apical attachment were observed between these regions (*p* = 0.5642; Supplementary Fig. 8a, b). Therefore, subsequent image analyses were conducted on randomly selected areas across the microtissue surface.

Time-resolved bacterial growth was monitored at *t* = 0, 2, 4, and 6 h under both flow (voiding/filling cycles) and static conditions. Voiding events (*t* = 0, 2, and 4 h) were applied exclusively under flow, with samples collected immediately post-voiding; parallel samples were obtained at matched time points under static conditions (Fig. 3b). Significantly lower CFU counts were recovered from the planktonic compartment at *t* = 2 and 4 h under flow conditions (*p* = 0.0097 and *p* = 0.0017, respectively). By 6 hpi, bacterial loads in the apical compartment were comparable between conditions. A similar trend was observed for the attached bacterial population, with significant reductions in CFUs at *t* = 2 h (*p* < 0.0001) and *t* = 4 h (*p* = 0.0148) under flow conditions, but no difference at *t* = 6 h (*p* = 0.8396) (Fig. 3c). Despite comparable bacterial loads at 6 hpi, barrier integrity was significantly better preserved under flow conditions (Fig. 3d), potentially reflecting reduced cumulative bacteria-epithelium contact over time (Fig. 3b, c). Bacterial attachment to the urothelium was reduced after the voiding-filling cycles in comparison with the static control at 2 and 4 hpi. However, at 6 hpi, similar bacterial coverage area was observed for the static vs flow conditions (Fig. 3e, f). These findings indicate that, despite the mechanical challenges imposed by voiding/filling-associated shear stresses, UTI89 retains the capacity to adhere to the urothelium and establish infection-associated growth over time.

Strikingly, *E. coli* UTI89 underwent marked bacterial elongation/filamentation, as observed by both confocal and electron microscopy under the filling-voiding cycles (6 hpi), which was observed only rarely in the static model (Fig. 3f–h). To assess the impact of flow-induced wall shear stress on bacterial morphology during infection, the cumulative fraction of bacterial objects as a function of their geodesic diameter (i.e., a measure of cell length) under static (no flow) and dynamic (flow) conditions was quantified using our 3D-bacterial segmentation image analysis pipeline named BactoMeasure3D^45^. Cumulative distribution analysis across the 2, 4, and 6 h time points revealed consistent differences in bacterial size profiles between conditions (Fig. 3i). Under static conditions, size distributions were strongly left-shifted at all time points, with the cumulative fraction rapidly approaching 1.0 at relatively short geodesic diameters, indicating a predominance of smaller bacterial cells. In contrast, flow conditions produced broader distributions that were progressively right-shifted, with a more gradual increase in cumulative fraction and enrichment of larger objects. This divergence was evident as early as 2 h and became more pronounced over time, consistent with a flow-dependent increase in cell elongation. This was further evidenced by the AUC of the cumulative fraction plots (Fig. 3i). Under dynamic conditions, AUC values gradually increased over time from 5.83 to 13.1 for 2 h and 6 h, respectively, whilst under static conditions AUC changed from 6.67 to 6.43; a statistical difference between static and dynamic was only observed 6 hpi (*p* = 0.0399). In the HSS and ISS regions after voiding-filling cycles, the extent of bacterial cell elongation was comparable (Supplementary Fig. 8d). To assess whether the observed bacterial elongation was a consequence of the alternating filling-voiding cycles (and the associated temporal change in wall shear stress levels), we tested a flow protocol without the voiding phases. The filling phase alone (6 hpi) induced cell elongation as observed in the confocal images (Supplementary Fig. 8b–d), suggesting that the low wall shear stress regimen representative of bladder filling was sufficient to induce bacterial elongation and that alternation of low and high wall shear stress levels was not the primary mechanism. These findings suggest that flow-induced filamentation represents an adaptive response to the bladder mechanical environment that may enhance bacterial attachment and surface colonization under hydrodynamic stress. Filamentous UPEC morphologies have also previously been associated with reduced susceptibility to phagocytic clearance, supporting the concept that morphological plasticity constitutes a mechanism by which bacterial pathogens can subvert host immunity^46^.

To assess whether mechanical cues influence bacterial redox metabolism, we quantified resazurin reduction, a proxy for overall cellular metabolic activity and respiration. The planktonic population was recovered 6 hpi in both static and flow conditions. As observed in Fig. 3j, reduced metabolic activity was observed for the flow population in comparison with the static conditions (*p* = 0.029). Additionally, bacterial length analysis of the planktonic populations revealed a higher proportion of filaments with respect to rods under flow conditions (*p* < 0.001), as shown in Fig. 3k. Together, these findings indicate that flow-induced mechanical cues modulate bacterial physiology, leading to reduced cellular redox activity and increased filamentation within the planktonic population.

To determine whether our observations held true beyond UTI89, we next assessed two clinical isolates in the P-FLO system: *E. coli* EC3 (derived from urine of a UTI patient) and COM2 (derived from asymptomatic donor urine). As observed for UTI89, EC3 showed significantly fewer planktonic CFUs under flow at 2 and 4 h (*p* = 0.0007 and *p* = 0.0442), with no difference at 6 h (*p* = 0.8525) (Supplementary Fig. 9a). COM2 CFUs were also reduced under flow, but more severely than for EC3 (*p* < 0.0001) (Supplementary Fig. 9B). Under static conditions, COM2 exhibited higher urothelium-associated CFU counts than did EC3 (1-log increase), indicating enhanced urothelial attachment. Flow did not impair EC3 urothelial association, with comparable CFUs (*p* = 0.8447) and surface coverage (*p* = 0.634) to static conditions, whereas COM2 CFUs were reduced (p = 0.024), alongside reduced attachment (*p* = 0.112) (Supplementary Fig. 9c–e). Confocal imaging revealed pronounced EC3 filamentation at 6 h post-infection under filling/voiding cycles. In contrast, COM2 did not filament, but rather formed aggregates under both static and flow conditions (Supplementary Fig. 9e). As shown in Supplementary Fig. 9f, the metabolic activity of COM2 planktonic population was markedly reduced under flow in comparison with static conditions (*p* = 0.0091), similarly to UTI89 (Fig. 3j). However, in EC3, a similar metabolic activity was observed in both conditions (*p* = 0.1273). Additionally, we investigated the bacterial length for the planktonic population in static and flow conditions, and no statistical differences between the rod/filamentation proportions were observed for EC3 and COM2 in static versus flow conditions (Supplementary Fig. 9g).

Together, these results suggest that fluid dynamics is a key regulator of UPEC infection, limiting early bacterial expansion and driving pronounced, strain-specific morphological and metabolic adaptations, which collectively may impact the ability of different UPEC strains to establish infection on the urothelial surface.

### Fluid flow affects antimicrobial susceptibility (for both bacteriophage and antibiotics) against *E. coli* UTI89 in the 3D-UHU urothelial microtissue model

**Fig. 4:**
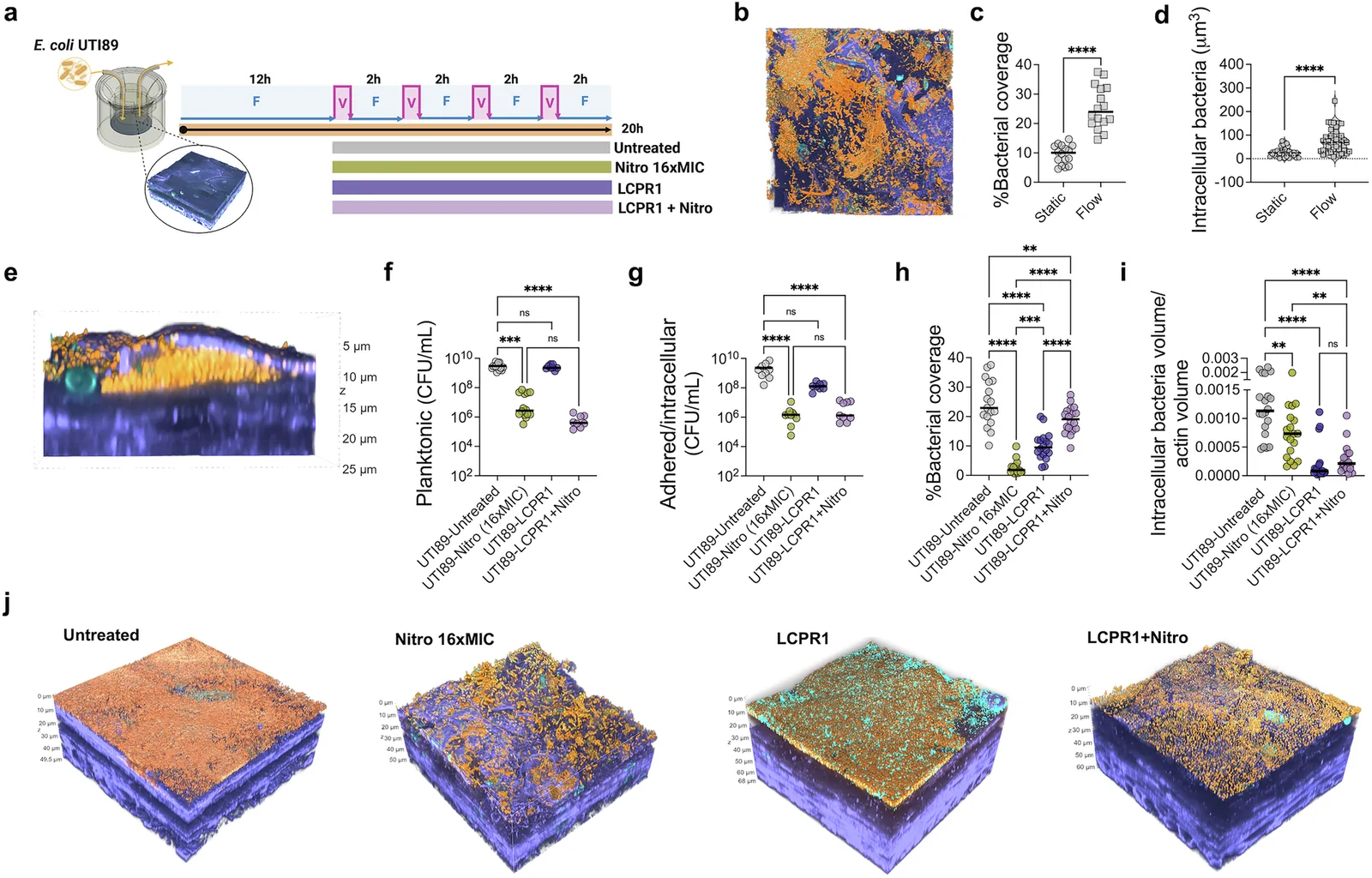
Fluid flow attenuates phage and antibiotic treatment response in 3D-UHU infected with *E. coli*. **a** Experimental set-up. Created in BioRender. Garcia Maset, R. (2026) https://BioRender.com/ac40vcd. **b** Top view of representative 3D confocal micrograph of untreated *E. coli* UTI89-GFP (orange) 20 hpi under flow in 3D-UHU; DNA (cyan), F-actin (purple). **c** UTI89-GFP coverage of urothelial surface (20 hpi, untreated) under dynamic vs. static conditions. *N* = 3 biological experiments described in Fig. 3; each circle represents five images acquired/analysed per biological experiment as technical replicates. Horizontal lines indicate mean values. A Welch’s two-sided *t-*test compared dynamic vs static groups. **d** Intracellular bacterial quantification in models (20 hpi, untreated) under dynamic vs static conditions. *N* = 3 biological experiments as defined above; each circle represents ten images acquired/analysed per biological experiment as technical replicates. Horizontal lines indicate mean values. Welch’s two-sided *t*-test as in (**c**). **e** Cross-section in xz plane illustrating an IBC under flow conditions, colours as in (**b**). **f** Bacterial load in apical (planktonic) and **g** attached/intracellular population post-treatment 20 hpi under dynamic conditions. Two-sided Kruskal-Wallis test compared treated vs untreated. Each circle represents one bladder microtissue, *n* = 4 biological experiments with two technical replicates as defined above. **h** Bacterial coverage of urothelial surface post-treatment in dynamic conditions. *N* = 3 biological experiments as defined above; each circle represents five-six- images acquired/analysed per biological experiment as technical replicates. Horizontal lines indicate mean values; ordinary two-sided ANOVA test compared different groups. **i** Intracellular bacterial quantification post-antimicrobial treatment under flow conditions, *n* = 3 biological experiments as defined above; each circle represents eight images acquired/analysed per biological experiment as technical replicates. Horizontal lines indicate mean values; ANOVA as in (**h**). **j** Representative 3D renderings of confocal micrographs of UTI89 20 hpi under flow conditions with conditions indicated (colours as in **b**). For all graphs, statistical significance was defined as none (ns), *p* ≥ 0.05; *p < 0.05; **p < 0.01; ***p < 0.001; and ****p < 0.0001.

Given that more physiological flow dynamics allows continuous nutrient exchange and exerts wall shear stress on the urothelium, both of which could influence pharmacodynamics, we next used the P-FLO 3D-UHU system to determine how flow affected the antimicrobial susceptibility of UTI89 during infection. After the microtissue was infected with *E. coli* UTI89-GFP, a continuous flow of 0.02 min^−1^ was applied for 12 h to simulate bladder filling. Following the 12 h infection, either nitrofurantoin (16xMIC, 256 μg mL^−1^), LCPR1 (1 × 10^9^ phage mL^−1^) ora dual treatment containing nitrofurantoin and LCPR1, and compared with an untreated control. To mimic urination cycles, the flow was interrupted with 1-minute voiding events at an increased flow rate of 0.7 mL min^−1^ every 2 h for a total of 8 h (Fig. 4a). The addition of flow at 6 hpi resulted in a similar bacterial burden and attachment in the 3D-UHU in comparison with static infection, despite increased bacterial cell elongation under flow (Fig. 3c, f–h). Similarly, at 20 hpi, the cell elongation phenomenon was still observed (Fig. 4b and Supplementary Fig. 10a). Image analysis at 20 hpi (Supplementary Fig. 2a and 10b) at 40× magnification demonstrated that flow conditions markedly increased bacterial attachment to the urothelium, with 22% coverage under flow compared with 10% under static conditions (Fig. 4c), despite comparable urothelium-associated CFU counts between the two conditions. Furthermore, the bacterial invasion strategy of UTI89 previously observed in static conditions (Fig. 2i) was not impaired under flow conditions; indeed, a greater proportion of IBCs were observed under mechanical stress cues (Fig. 4d, e), in comparison with the static model (Supplementary Fig. 6). Taken together, these results suggest that flow conditions promoted UTI89 attachment and intracellular invasion compared with our previously established static infection model, indicating a role for hydrodynamic forces in enhancing these virulence attributes.

To examine the effect of flow conditions on treatment, we first tested nitrofurantoin and found that it resulted in significantly reduced planktonic bacterial burden in the apical compartment, with a ~3-log CFU reduction compared with the untreated control (Fig. 4f; *p* < 0.0001). Against urothelium-associated bacteria, nitrofurantoin achieved a ~3-log reduction (Fig. 4g; *p* < 0.0001). Concordantly, nitrofurantoin produced the greatest reduction in bacterial coverage area (Fig. 4h and Supplementary Fig. 10b).

Nitrofurantoin exhibited distinct killing kinetics against planktonic and urothelium-associated bacterial populations under static versus flow conditions. In static conditions, we observed a greater reduction in planktonic CFUs (*p* = 0.0019), whereas under flow, nitrofurantoin demonstrated a non-significantly different antimicrobial activity (*p* = 0.1547) against urothelium-associated bacteria in comparison with static conditions (Supplementary Fig. 10c, d). Notably, despite increased overall drug exposure under flow, the antibiotic activity against planktonic bacteria was diminished.

LCPR1 failed to reduce bacterial burden under flow conditions to the same extent observed in static environments (Fig. 4f, g and Supplementary Fig. 10c, d). LCPR1 treatment reduced bacterial coverage area relative to the untreated control (*p* < 0.0001), although to a lesser extent than nitrofurantoin (*p* = 0.02), despite not significantly reducing urothelium-associated CFUs (Fig. 4g, h and Supplementary Fig. S10b). This discrepancy may reflect an effect of LCPR1 on plasmid-associated fluorescence in UTI89, as some bacterial populations in Fig. 4j were detected by DAPI staining alone.

The dual treatment did not outperform the antimicrobial effect of nitrofurantoin alone, with comparable bacterial loads detected in both planktonic and urothelium-associated populations under flow (Fig. 4f, g). Bacterial coverage area was also significantly reduced compared with the untreated condition (Fig. 4h; *p* = 0.071 and Supplementary Fig. 10b), although the magnitude of reduction was less pronounced than that observed with nitrofurantoin or LCPR1 alone (*p* < 0.0001, respectively). Given that flow conditions promoted increased bacterial surface attachment (Fig. 4c), these findings may indicate that, following dual treatment, a larger proportion of bacteria remained associated with the urothelial surface despite being non-viable (Fig. 4g), and were therefore still detected in the coverage analysis (Fig. 4h).

The most striking difference in treatment efficacy emerged when comparing the dual therapy’s impact on planktonic bacteria between static and flow conditions (*p* < 0.0001). While complete eradication was achieved in static environments, flow conditions yielded only a ~4-log reduction, highlighting how mechanical forces and microenvironmental cues under flow conditions might impact host-pathogen interactions and bacterial physiology, thereby modulating the overall antimicrobial efficacy (Figs. 4f and 2b and Supplementary Fig. 10c, d). Phage recovery of the planktonic compartment from the flow model showed higher titres in the LCPR1-only group compared with the dual-treatment, consistent with the lower bacterial counts observed under dual treatment in static conditions (Supplementary Figs. 10e and 5a, b). Compared with the static model, phage titres were similar overall, with lower phage counts in the adherent/intracellular compartment (Supplementary Figs. 10f and 5c). Phage UP17 showed reduced titre levels both in the apical and microtissue compartments.

Given that IBC formation in untreated flow conditions was enhanced, we investigated the effect of the antimicrobial therapies against intracellular bacterial communities. Nitrofurantoin decreased CFU within the adhered/intracellular compartment (Fig. 4g) and reduced IBC prevalence (Fig. 4i), whereas LCPR1 limited IBC formation without affecting the overall adhered CFUs (Fig. 4g, i), and the combination treatment reduced both bacterial burden and IBC abundance (Fig. 4g, i).

Together, these findings demonstrate that flow conditions promote bacterial surface attachment and intracellular invasion by UTI89. Although dual treatment and nitrofurantoin monotherapy showed comparable efficacy in reducing overall bacterial burden, combination treatment resulted in a greater reduction in intracellular bacterial communities (IBCs). Treatment responses in urothelium-associated bacteria were broadly similar between static and flow conditions, whereas antimicrobial activity against planktonic populations was substantially reduced under flow conditions, underscoring the importance of more physiologically relevant infection models for evaluating antimicrobial efficacy.

## Discussion

At least ~260,000 deaths were associated with UTIs linked to AMR in 2019^47^. Recurrent UTIs are extremely common, with ~30% of all women experiencing a recurrence within one year of their original infection, and some of these experiencing repeated infections thereafter^48^. Poor treatment outcomes despite appropriate antibiotic therapy highlight the limited predictive value of standard in vitro antimicrobial susceptibility testing^14^. Heithoff et al. showed that, by more closely recapitulating the physiological conditions of infection (i.e., presence of urine, serum or cell culture media) in comparison with standard bacterial growth media (MHB), it was possible to achieve a closer prediction of antimicrobial performance in the clinical setting^29^. In a similar manner, we showed that three UPEC isolates had an increased MIC against cefalexin when 70% pHU was used, crossing the defined EUCAST breakpoint^29^. However, most antibiotics were largely unaffected, suggesting that additional factors besides urine might lead to a lack of concordance between MIC and clinical outcomes.

Nitrofurantoin demonstrated the most potent antimicrobial activity against all the isolates tested in both standard and urine-containing media. As we employed the cationic-adjusted version of MHB (caMHB) with divalent cations at physiological concentrations, a less marked difference between 70% pHU and caMHB microenvironments compared with Heithoff and colleagues’ work was expected. However, when the granularity of the assay was increased by using time-killing assays instead of the fixed endpoint MIC, the efficacy of nitrofurantoin against UPEC UTI89 was shown to be reduced in the urine microenvironment despite this strain’s sensitivity in the standard caMHB-based MIC.

Advances in the bioengineering and microfluidic fields in the last decade have led to the development of organoids and organ-on-chip technologies, which provide a more physiologically relevant platform to investigate infectious diseases^49,50^. For example, a human mini-bladder model incorporating a stratified urothelium, urine perfusion and micturition-like dynamics demonstrated that high-solute urine conditions reduced fosfomycin efficacy and promoted the emergence of tissue-associated cell wall-deficient UPEC populations. This work exemplifies the value of advanced bladder microphysiological systems for investigating how host microenvironments shape antimicrobial activity and bacterial persistence in UTI^22,51^. Hence, we wanted to explore how a urothelial-cell associated environment might further affect the efficacy of nitrofurantoin against UTI89.

The 3D-UHU microtissue model supports the formation of both intracellular bacterial communities (IBC) and surface-associated bacterial clusters and biofilms, both of which represent key pathogenic adaptations employed by UPEC during infection^23^. UPEC invasion has previously been observed many times in mice (e.g.,^52^), shed patient cells (e.g.,^53^), human bladder cell lines (e.g.,^54^), and human urothelial biopsies (e.g.,^55^). In 3D-UHU, while nitrofurantoin significantly reduced bacterial burden, it did not cause complete eradication for the duration of our experiments, even at a concentration achieved with regular clinical dosing. Strikingly, IBCs persisted following nitrofurantoin treatment despite an overall reduction in bacterial surface coverage, supporting the idea that protected urothelial niches can contribute to bacterial survival despite the presence of effective antibiotics in the urine.

In an era of increasing antimicrobial resistance, the development of alternative therapies such as bacteriophages has gained popularity^56^. We evaluated a novel bacteriophage cocktail (LCPR1) both in vitro and in the 3D-UHU model. Within the urine microenvironment, LCPR1 exhibited a reduced antibacterial activity compared with that observed in nutrient-rich media. Similarly, Lui et al. reported decreased phage efficacy in urine relative to rich media^57^. These findings underscore the importance of assessing phage performance under infection-site conditions to more accurately predict clinical efficacy. In the infected 3D-UHU model, under static (non-flow) conditions, LCPR1 alone reduced IBC formation and attenuated cellular cytotoxicity. This was accompanied by lower secretion of IL-1β, but not other cytokines tested. The coupling of LDH and IL-1β release suggests a link between cellular cytotoxicity and inflammasome activation in urothelial cells after UPEC infection. In contrast, bacteriophage co-infection augmented secretion of the pro-inflammatory cytokine TNFα alongside the neutrophil chemokines and maturation factors CXCL2, IL-8 and G-CSF. We hypothesise that phage-mediated lysis of *E. coli* releases bacterial ligands that bind to Toll-like receptors expressed by 3D-UHU^24^, signalling via NF-kB and promoting inflammation, neutrophil chemotaxis and maturation, further supporting bacterial clearance. Such a model requires testing in more complex experimental systems, and does not exclude direct host-phage interactions that may directly modulate the urothelial response to UPEC interaction^58–60^, evidenced by direct induction of several chemokine/cytokines following 3D-UHU exposure to LCPR1 alone.

Compared with the use of phage or antibiotics alone, combination therapy has been reported to provide superior efficacy in suppressing bacterial populations and curbing the evolution and spread of antimicrobial resistance^61^. In the static 3D-UHU model, the dual treatment (i.e., LCPR1 + nitrofurantoin) achieved a total eradication of the planktonic bacteria, outperforming the individual treatments. However, while the combination treatment could not clear the overall microtissue-associated bacterial population, it did appear to recapitulate key features of both monotherapies, producing reductions in bacterial burden and attachment comparable to nitrofurantoin alone while also reducing the proportion of intracellular bacterial communities, consistent with the effect of phage treatment. These data suggest that the therapeutic benefit of the combination may derive from complementary targeting of extracellular and intracellular niches, rather than from increased overall bactericidal activity. Further studies will be required to determine how efficacious phages will ultimately prove to be for UTI treatment in the clinic.

The urinary system has an essential and specific fluid dynamic microenvironment. Despite this, most organoid/microtissue infection work has been performed in static conditions, most likely because of the complexity and cost of commercially available flow-integrated systems. Introducing flow, and its concomitant wall shear stress and nutrient gradients, into model systems is essential to more faithfully reproduce the biophysical and biochemical cues that shape host-pathogen interactions in vivo^51^. To explore pathogenic strategies in the gut mucosa, Mikhaleva et al. established a mucus-secreting human colonoid tissue under flow that replicated conditions experienced in the gastrointestinal tract to investigate *E. faecalis* pathogenicity^62^. In UTI, Sharma et al. deployed a bladder-chip model that allows cyclic stretching and flow during the filling/voiding phases to investigate UPEC invasion and immune infiltration in real time^21^. These and other microfluidic platforms offer the advantage of single-cell resolution and the ability to generate finely controlled laminar flow conditions; however, they often lack the 3D complexity of the urothelium, and such systems are beyond the economic means and technical expertise of many laboratories.

For the current study, we designed a relatively inexpensive mesofluidic system called P-FLO to incorporate controlled flow and cyclical shear stress associated with bladder filling and voiding onto the apical side of commercially available 12-well plate Transwells, which is compatible with the differentiated urothelial 3D-UHU model and many other organoid and microtissue systems. We have made these designs freely available to the community for widespread adoption across laboratories. Using P-FLO, we showed that hydrodynamic conditions substantially reshape UPEC infection dynamics. Filamentation was induced rapidly under flow conditions, occurring in UTI89 from 2 h post-infection onwards and in the clinical isolate EC3 at 6 h post-infection. Although some filamentation occurred in static conditions, 6 h of flow increased the frequency of UTI89 elongation in groups that were exposed to both filling/voiding cycles (with alternating low and high wall shear stress levels) or to filling only. Notably, exposure to the filling phase alone (6 hpi) was sufficient to induce UTI89 filamentation, indicating that the low wall shear stress conditions characteristic of bladder filling can independently promote filamentation. Bacterial attachment was enhanced during the filling phase alone and maintained under filling/voiding flow conditions, with bacterial coverage and burden remaining comparable to those observed under static conditions. Together, these findings suggest that cyclic alternation between low and high wall shear stress is not the principal trigger for this phenotype.

Bacterial filaments are commonly found in UTI^53^, and they can be triggered by stress responses (including pH, UV light, or antibiotics)^63^. Studies in murine UTI models have revealed that UPEC filamentation is an adaptative mechanism that promotes bacterial survival, allowing UPEC to evade or delay phagocytosis by neutrophils and macrophages^64,65^. Additionally, Aguilar-Luviano et al. have shown that filaments confer protection during toxic stress by decreasing the surface area to volume ratio, which slows the diffusion and accumulation of harmful compounds within the bacterial cell^66^. Isofidis et al. characterized the filamentous IBC dispersal at a single-cell level using a microfluidic bladder cell model, and showed that the urinary environment seemed to trigger UPEC filamentation, perhaps due to acidic pH or some urine component^67^. However, the role of mechanical stimuli, such as shear stress, remains unexplored. Recently, Nadal et al. showed that buckling of *E. coli* filaments alters Min protein pattering and division site placement, a clear demonstration that mechanical forces and filament geometry can feed back to the cell division machinery^68^.

In previous 2D bladder flow culture models, UPEC filamentation was observed only during later stages of infection, following prolonged intracellular colonisation, gentamicin-mediated elimination of extracellular bacteria and subsequent bacterial escape (“fluxing”) from urothelial reservoirs during secondary surface colonisation under urine flow^69^. Our findings suggest that hydrodynamic shear stress and urothelial surface-associated microenvironmental cues may directly induce early filamentation independently of the classical intracellular infection cascade, since UPEC filaments were observed at early infection time points (2–6 h).

In addition to effects on filamentation, flow also enhanced the attachment of UTI89 to the urothelial surface and increased IBC formation at later infection time points (20 h), indicating that hydrodynamic cues substantially altered bacterial behaviour during infection. The increased urothelial attachment observed under flow conditions is consistent with the established role of FimH-mediated adhesion during UTI pathogenesis. FimH-dependent binding to mannosylated urothelial receptors is required for bladder colonisation, intracellular invasion and IBC formation in vivo^70^. Previous studies have also demonstrated that shear forces enhance FimH-mannose interactions through catch-bond behaviour, promoting bacterial retention under hydrodynamic stress^71^. The enhanced bacterial attachment and intracellular invasion observed in the flow-enabled 3D-UHU model therefore support the idea that biomechanical stimuli, such as wall shear stress, actively contribute to UPEC persistence by reinforcing urothelial colonisation and invasion pathways that are central to infection progression. Future flow models that combine mechanical stretch in the context of a fully differentiated urothelium will shed more light on these interesting persistence strategies.

Although antimicrobial exposure was increased under flow conditions, neither nitrofurantoin, LCPR1 nor the combination treatment achieved greater reductions in urothelium-associated bacterial burden compared with the static model. In contrast to the urothelium-associated population, antimicrobial activity against planktonic bacteria was diminished under flow. This effect may reflect the reduced metabolic activity of UTI89 observed in the P-FLO/3D-UHU model at 6 hpi in comparison with the static model, which could contribute to decreased bacterial susceptibility to antimicrobial treatment. Nitrofurantoin and the dual treatment produced substantially lower reductions in planktonic bacterial burden under flow than under static conditions, where complete eradication was observed following combination therapy. Despite this, combination treatment reduced IBC abundance more effectively than did nitrofurantoin alone, indicating differential treatment responses between extracellular and intracellular bacterial populations. Our findings demonstrate that more physiological flow conditions strongly influence UPEC infection dynamics and antimicrobial susceptibility, emphasizing the importance of incorporating hydrodynamic forces into preclinical infection models—while acknowledging that P-FLO does lack other important cues, such as broader mechanical and volumetric dynamics of true bladder filling/micturition cycles including urothelial stretching, pressure changes and urine accumulation. Future studies with more diverse strains of *E. coli*, alongside other species of uropathogenic bacteria, would also be of interest to determine how universal such effects are.

In conclusion, we examined the antimicrobial activity of the widely used antibiotic nitrofurantoin against UPEC across progressively complex environments. These included human urine; a static human urothelial microtissue model; and a novel mesofluidic urothelial model built from simple, low-cost 3D-printed components to create a more physiologically relevant flow-based environment. In addition, we employed both the static and mesofluidic models to evaluate the efficacy of bacteriophages alone and in combination with antibiotics. Collectively, these experiments revealed the profound influence of microenvironmental context on UPEC infection dynamics and treatment response.

A limitation of this study is the restricted diversity of treatments evaluated in the infection experiments. We chose to focus specifically on two fundamentally different antimicrobial approaches: phage therapy and the most widely used antibiotic for UTI treatment, both individually and in combination. This strategy allowed us to directly compare conventional antimicrobial treatment with phage-based therapy within the same experimental framework. Nevertheless, evaluation of additional antibiotic classes in future studies would help determine whether the observed effects are generalizable. In addition, we focused on treatment failure during therapy administration, so while our findings demonstrate incomplete bacterial clearance under these conditions, future studies in this model should explore post-treatment regrowth dynamics^22^. Additional improvements to our model could include the use of donor-derived stem cells instead of the commercially available HBLAK cells, which would allow assessment across different host backgrounds and the use of autologous immune cells.

Although human cell-based 3D organoid, microtissue and organ-on-chip models lack crucial in vivo animal model components such as a blood supply, a complete repertoire of other cell types and a full immune system, they are nevertheless emerging as crucial complementary approaches to animal models, providing a human-specific micro-environment to understand host-pathogen interaction and treatment responses. Further development and incorporation of complexity into these systems will generate an increasingly valuable platform for discovery science in infection biology as well as for testing novel antimicrobial strategies.

## Methods

### Ethics

For decellularised urine harvested from healthy adult volunteers, we operated under ethics granted by the UCL Research Ethics Committee (Project ID 23285/002), conforming to all relevant ethics regulations, including informed consent.

### Bacterial strains

Uropathogenic *Escherichia coli* (UPEC) strains used in this study included CFT073 (pyelonephritis isolate) and HM50 (asymptomatic bacteriuria isolate), obtained from the American Type Culture Collection (ATCC). UTI89, isolated from patients with acute cystitis and its GFP-expressing derivative (UTI89-GFP), were generously provided by Scott Hultgren’s lab (Washington University in St. Louis, USA). UTI89-RPF^72^ was provided by Molly Ingersoll’s lab (Institut Cochin, Paris). The non-pathogenic laboratory strain *E. coli* MG1655 was kindly provided by Meriem El Karoui (University of Edinburgh, UK). The clinical isolates from Royal Free Hospital (London, UK) include three previously described UPEC strains isolated from clean-catch midstream urine samples of patients with chronic UTI (ClinA, EC3 and EC4), and two commensal *E. coli* isolates (COM1 to COM2) similarly recovered from healthy individuals^73^. Bacterial strains were routinely plated on Luria Broth (LB) agar (BD Difco LB agar, Lennox) and supplemented with kanamycin (50 μg ml^−1^) for the GFP or RFP expression depending on the strain used.

### mCherry (pGI6) transformations

mCherry (pGI6) plasmids^74^ were kindly provided by Bill Söderström’s lab. Briefly, 3-4 colonies from a fresh agar plate of COM2 and EC3 were resuspended in 1 mL of ice-cold 10% (v/v) glycerol (Sigma-Aldrich) in distilled water and centrifuged at 5000 × *g* for 1 min. The pellet was broken down by pipetting the residual medium, and 2 μL of plasmid DNA was added (20 μg mL^−1^) and incubated on ice for 5 min. Electroporation was performed in 1 mm gap cuvettes using a Bio-Rad Gene Pulser Xcell (EC1 program). After electroporation, 1 mL of SOC media was added and transferred to a 5 mL Corning tube and cultured for 1 h at 37 °C under shaking conditions (200 rpm). Then, the bacterial solution was plated on LB agar supplemented with 100 μg mL^−1^ of spectinomycin and incubated at 37 °C overnight. Fluorescent colonies were observed with a Leica DM2500 epifluorescence microscope to confirm plasmid transformation and isolated to make bacterial stocks; the resulting strains were named COM2-mCherry and EC3-mCherry. Bacterial strains were routinely plated on LB agar (BD Difco LB agar, Lennox) and supplemented with 100 μg ml^−1^ of spectinomycin for the mCherry expression.

### LCPR1-Phage cocktail propagation and purification

The optimised bacteriophage cocktail LCPR1 has been developed for another project as a broader-spectrum therapy for UTIs caused by *E. coli* or *Klebsiella pneumoniae*. Host liquid propagation was used for each phage with its respective host (Table 1). In brief, bacterial strains were cultured to log phase (OD_600_ ~ 0.15–0.2) using an overnight culture as a starting point. 200 μl of bacterial culture and 200 μl of phage stock were then added to 20 mL of LB medium and incubated in a shaking incubator at 37 °C overnight (~18 h). The next day, the lysate was centrifuged at 4000 × *g* for 10 min and the supernatant was filter-sterilised through 0.2 μm pore-sized filters (Fisherbrand™). The phage stock titre was determined using plaque assays^75^ and phage stocks were stored at 4 °C. The phage cocktail was produced by mixing all 9 phages in a 1:1 ratio (10^11^ PFU mL^−1^ each of phage stock), then diluted for the final LCPR1 cocktail to a concentration of 10^10^ PFU mL^−1^ 1 mL of purified phage cocktail was then run through an Endotrap HD column (LIONEX, GmBH) using gravity following the manufacturer’s instructions. 200 μl of void volume was collected in a separate tube.

**Table 1:** Phages included in LCPR1 cocktail and original propagation hosts used in this study

Phages	Source of Isolation	Accession No.	Host Strain	Host Source	Accession No.
*E. coli* Phages					
vB_EcoM_UP17	Sewage	ERS3957426	*E. coli EA2*	Leicester Royal Infirmary, UK	ERZ1207058
JK03	Sewage	OZ470850	*Shigella B31*	Leicester Royal Infirmary, UK	ERS29408128
Leic_001	Sewage	OZ470821	*E. coli MH10*	Leicester Royal Infirmary, UK	ERZ1207058
Klebsiella Phages					
vB_KpnM_311F	Sewage	LR877331	*K. pneumoniae KR311*	Leicester Royal Infirmary, UK	ERZ1207061
vB_KpnM_05F	Sewage	LR746310	*K. pneumoniae MH05*	Leicester Royal Infirmary, UK	ERZ1207078
vB_KppS_Anoxic	Sewage-anoxic sludge	LR881109	*K. pneumoniae 30104*	DSMZ Culture Collection (Human Blood)	AJ233420
vB_KpM_SoFaint	Sewage-mixed liquor	LR881141	*K. pneumoniae 13440*	NCTC Culture Collection (Clinical)	UGKX00000000
vB_KpM_Centimanus	Sewage	OZ240615	*K. pneumoniae 13440*	NCTC Culture Collection (Clinical)	UGKX00000000
vB_KppS_**Storm**	Sewage-storm tank	LR881112	*K. pneumoniae 30104*	DSMZ Culture Collection (Human Blood)	AJ233420

### Bacterial growth curves

Bacterial cultures were made by inoculating a single colony from fresh agar plates into 5 mL of LB broth (Sigma-Aldrich) and placed in an orbital shaking incubator at 37 °C for 24 h at 200 rpm. Cultures were adjusted to OD_600_ ~ 0.1 in phosphate-buffered saline (PBS, Gibco) and further diluted 1:100 in cation-adjusted Müller-Hinton broth (caMHB, Sigma-Aldrich); 100% pooled human urine (pHU); or a mixture of the two (70%pHU:30%caMHB). The pHU was obtained commercially (BioIVT) or obtained and decellularized from healthy volunteers of both sexes under ethics granted by the UCL Research Ethics Committee (Project ID 23285/002). Each batch of pHU, whether commercially sourced or collected, consisted of samples from 10/15 mixed sex donors. Then, 200 μL were transferred to flat-bottomed 96-well plates (ThermoFisher) in triplicate technical replicates, and growth curves were generated using a microtiter plate reader (Tecan Spark® 10 M), measuring OD_600_ every 30 min over 16 h at 37 °C. QurvE^76^ in RStudio was used to analyse growth curve data using a non-parametric fitting. Three biological experiments were performed with three technical replicates per experiment.

### Minimum inhibitory concentration (MIC)

MIC was determined according to the standard broth microdilution method by the European Committee on Antimicrobial Susceptibility Testing (EUCAST)^27,77^ using caMHB. Additionally, MICs were determined using pHU mixed in caMHB (70:30 v/v%). Overnight cultures of bacteria were grown in caMHB and in 70% pHU in shaking conditions at 37 °C. Bacterial cultures were adjusted to OD_600_ ~ 0.08-0.1 in PBS, corresponding to a bacterial concentration of ∼10^8^ colony forming units per mL (CFU mL^−1^). The solution was diluted 100-fold to obtain a concentration of 10^6^ CFU mL^–1^ in caMHB or 70% pHU. Nitrofurantoin (Sigma), cefalexin hydrate (Sigma), fosfomycin sodium (TOKU-E), trimethoprim (Sigma) or amoxicillin trihydrate (Merk) were dissolved in caMHB, 70% pHU or 100% pHU, and 100 μL of each antibiotic solution was added and serially diluted into the 96-well plates followed by the addition of the same volume of bacterial suspension, resulting in a final bacterial density of 5 × 10^5^ CFU mL^−1^. The microwell plates were incubated at 37 °C statically for 24 h, and growth was evaluated by eye. Triplicates were performed for each concentration. Bacteria alone or uninfected media were used as positive and negative controls, respectively. The experiment was repeated three times, and the highest MIC value was reported.

### Minimum bactericidal concentration (MBC) and dose-response

After MIC determination, the bactericidal effect of the antibiotics was evaluated by adding 20 μL of resazurin sodium dye (Cambridge Bioscience, UK) with a final concentration of 0.5 mg mL^−1^ per well^78^. The plates were then incubated for 6 h at 37 °C. A noticeable change of colour could be observed for bacterial cells exhibiting growth (pink) versus non-detectable growth (blue). The lowest concentration where blue colour was observed (bactericidal concentration) was recorded as the MBC. Three independent experiments were performed. After the incubation of resazurin, the fluorescence signal in the 96-well plate was measured in a Tecan Spark® 10 M plate reader (excitation wavelength = 571 nm; emission wavelength = 584 nm). The bacterial viability in each well was then calculated using the positive control (untreated bacteria) and the negative control or blank (sterile media with resazurin) as follows:

$$\frac{{{{{\rm{\lambda }}}}}_{584({{{\rm{treatment}}}})}-{{{{\rm{\lambda }}}}}_{584({{{\rm{blank}}}})}}{{{{{\rm{\lambda }}}}}_{584({{{\rm{positive}}}}{{{\rm{control}}}})}-{{{{\rm{\lambda }}}}}_{584({{{\rm{blank}}}})}}\times 100$$

### Time-killing assay

Overnight cultures in caMHB and 70% pHU:caMHB were grown at 37 °C under shaking conditions (200 rpm). These cultures were subsequently diluted to OD_600_ ~ 0.01 in their respective media and grown to mid-exponential growth phase (OD_600_ ~ 0.3), for approximately 2-3 h. Bacterial cultures were adjusted to OD_600_ ~ 0.08-0.1 in PBS. The bacterial solution was diluted 100-fold to obtain a concentration of 10^6^ CFU mL^−1^ in a total volume of 5 mL of caMHB or 70% pHU. Nitrofurantoin was added at the desired concentrations, and cultures were then placed in the shaking incubator at 37 °C. Samples (80 μL) were taken at several time points as shown on the resulting graphs. Serial dilutions were performed with PBS in 96-well plates and plated on LB agar plates for CFU enumeration. Three independent experiments were performed. Untreated bacteria were used as a positive control.

### Human bladder cells and the 3D-UHU urothelial microtissue 3D model

Spontaneously immortalised, non-transformed human bladder epithelial cells (HBLAK; CELLnTEC) were cultured as described by Flores et al.^23^. HBLAK cells were originally derived from a male bladder; transcriptomic profiling has previously shown that male and female bladders are highly concordant at the genome-wide level^79^. As UTIs are more common in females, a significant contribution to their increased clinical risk is likely due to anatomical differences and hormonal influence^80^. Briefly, HBLAK were cultured in CnT-Prime medium (CELLnTEC) under standard conditions (37 °C, 5% CO₂, humidified atmosphere). Cells at 80-90% confluence were passaged by first rinsing with calcium- and magnesium-free phosphate-buffered saline (PBS; Gibco), followed by enzymatic detachment using CnT-Accutase solution (CELLnTEC) for 10 min at 37 °C. Three-dimensional urine-tolerant human urothelial models (3D-UHU) were created as described by Flores et al.^23^. Briefly, between passages 9 and 15 (where the passage number refers to the passage after thawing factory vials from the manufacturer), HBLAK cells at 80-90% confluency were enzymatically detached and resuspended at a density of 5 × 10⁵ cells mL^−1^ in pre-warmed CnT-Prime Epithelial Proliferation medium (CnT-PR, CELLnTEC). 400 μL of cell suspension was seeded onto 12-mm diameter, 0.4-μm pore polycarbonate membranes in plastic Transwell inserts (Corning), with 1.5 mL of medium added to the basal compartment. After 48 h of incubation at 37 °C and 5% CO₂, the medium in both apical and basal compartments was replaced with CnT-Prime Epithelial 3D Airlift Medium (CnT-PR-3D, CELLnTEC), designated as day 0. Following 48 h incubation, the Transwells were transferred onto 12-well Thincert plates (Greiner) and the apical medium was substituted with 300 μL of sterile-filtered pooled human urine (BioIVT), collected from 10/15 healthy donors of both sexes, while fresh differentiation medium was replenished in the basal chamber (4.5 mL). Urine and medium were refreshed every 3–4 days until days 16–20, at which point the matured urothelial microtissue models were used for infections.

### Antimicrobial efficacy in the 3D-UHU model under static (non-flowing) conditions

Fully differentiated 3D-UHU microtissue models from day 16 were used for the infection. Overnight cultures of *E. coli* UTI89 (LB) and *E. coli* UTI89-GFP (LB + supplemented with 50 μg mL^−1^ of kanamycin) were cultured in static conditions over 2 days (~48 h)^81^. Bacterial concentration (bacterial cells mL^−1^) was quantified with the QUANTOM Tx Microbial Cell Counter (Logos) following the manufacturer’s protocol. Then, the bacterial overnight culture was resuspended in pHU at 5.0 × 10^6^ bacterial cells mL^−1^ (similar inoculum as the MIC assay). Next, the models were infected for 12 h at 37 °C with 5% CO_2_. After 12 h, two doses of antibiotic (nitrofurantoin, 256 μg mL^−1^), phage cocktail (LCPR1, 1 × 10^9^ phage mL^−1^), and the combination of both treatments (256 μg mL^−1^ and 1 × 10^9^ phage mL^−1^) were added in intervals of 4 h. Before and after the treatment addition, the urine in the apical compartment was collected and serially diluted for CFU enumeration on LB agar plates. At the end of the second treatment, urine was collected for CFU enumeration, and the rest was immediately centrifuged (10,000 × *g* for 5 min) for further analysis (see below). Next, the microtissue models were washed twice with PBS and lysed with 1% Triton X-100 (ThermoFisher) for 15 min at 37 °C, followed by a manual physical disruption to enumerate CFUs of the intracellular and attached/intracellular communities. As positive control, infected models without treatment were used, and uninfected samples were used as negative controls. Two microtissues per condition were used, and three independent experiments were performed.

### Phage recovery assay

After the microtissue model was homogenised for CFU enumeration, the samples were centrifuged at 4000 × *g* for 10 min for a phage counting spot assay on double layer agar. Briefly, three *E. coli* phage hosts (EA2, B31, and MH10) were grown overnight and cultured to log phase (OD_600_ = 0.15–0.2). Double-layer agar was then prepared by adding 100 μL of bacterial culture to 8 mL of 0.7% (w v^−1^) LB agar, then poured onto square LB 1.5% (w v^−1^) agar plates. The sample supernatant was serially diluted with SM Buffer [100 mM NaCl (Sigma-Aldrich, United Kingdom)], 8 mm MgSO4•7H2O (Sigma-Aldrich), 50 mM Tris-HCl pH 7.5 (Sigma-Aldrich), then spotted onto dried double-layer agar and incubated 37 °C overnight ( ~ 18 h).

### LCPR1 planktonic killing assay (PKA)

*E. coli* UTI89 overnight cultures were diluted 1:100 either in Mueller Hinton broth (MHB) or 70% pHU:30% MHB (70% pHU:MHB) and added to flat-bottomed 96 well plates (Fisher Scientific, UK) in triplicate per treatment. The bacteria were grown to OD_600_ 0.1 (1 × 10^8^ CFU mL^−1^), and 100 μL of phage cocktail (containing 10^8^ PFU mL^−1^ of each individual phage) was added to each well, with dilution of phages made using MHB or 70% pHU:MHB. The PKA was performed at a multiplicity of infection (MOI) of 0.1, 1, and 10. The experiments were performed using the BMG Labtech SPECTROstar Omega, with measurements every 5 min (OD_600_) for 24 h, with shaking of 10 s before each reading. Three independent biological experiments were performed. Additionally, serial dilutions were performed with PBS in 96-well plates and plated on LB agar plates for CFU enumeration to quantify bacterial killing, and for the phage recovery assay (as described above) to quantify phage infectivity.

### Plug-Flow Linked Organoid (P-FLO) system

The P-FLO system utilised in this work was designed and manufactured in-house. A bespoke polydimethylsiloxane (PDMS) plug was designed for integration with 12-well plate Transwells (Corning) to allow continuous flow in the apical compartment of the insert. The plug featured inlet and outlet ports to enable flow through the model. The Transwell plugs were manufactured by replica-moulding using 3D-printed resin moulds. The material used for the plugs was PDMS (Sylgard^TM^ 184) at a 10:1 weight ratio of PDMS-to-curing agent. The computer-aided design (CAD) files for the moulds (in STL format) are freely available for download and use^82^. The mould model designs were sliced using PreForm software (Formlabs) and then 3D-printed using a Formlabs Form 3B + SLA 3D printer in Grey resin V4 material (Formlabs). The print resolution was set to 50 μm. To enable effective curing and detachment of PDMS from the moulds, these were treated for 10 h at 80 °C and lined with a thin layer of silicon spray (HD-Sil Release, Ambersil) before pouring of liquid PDMS. Once poured over the moulds, PDMS was allowed to cure overnight at 60 °C, and the solidified part was subsequently removed from the moulds. A New Era NE-1000 syringe pump (New Era Pump Systems Inc., Farmingdale, NY, USA) and PTFE 16 mm diameter tubing (Darwin Microfluidics) were utilised to deliver media through the P-FLO system at the desired flow rate. 20 or 60 mL capacity syringes (Fisher Scientific) were used as a media reservoir in this work.

### Numerical simulation of the fluid dynamic field within the P-FLO system

The P-FLO system was developed to simulate the levels of wall shear stress to which the bladder urothelium is subjected during bladder filling and voiding. Data on such wall shear stress values are underreported in the literature, due to impracticality of measurement and significant individual variability. A generally accepted estimation for shear stress on the bladder wall during filling is 0.02 (N m^−2^)^83^. During voiding, the estimated wall shear stress increases 10- to 100-fold for a short period of time^83,84^. Two different volumetric flow rate values were thus selected for this work, to simulate bladder filling and voiding, respectively. These were estimated by manual calculation to be 0.02 mL min^−1^ and 0.7 mL min^−1^, respectively. Subsequently, a CFD simulation was run on the P-FLO model to confirm the expected shear stress profile acting over the microtissue surface area. Given the relatively low flow rate necessary to obtain wall shear stress values in the 0.017 to 1.7 Pa range, the flow regime was modelled as laminar (the highest Reynolds number expected in the system is estimated to be ~12). Before starting the experiment, both the apical and basolateral sections of the P-FLO and the tubing were prefilled with urine to avoid entrapment of air bubbles in the system. Although the model was designed to approximate the shear stress experienced by bladder’s endothelial cells, the total apical volume in the P-FLO system is substantially lower than that of a human bladder, amounting to ~357 mm^3^.

A steady-state laminar flow simulation was carried out using COMSOL Multiphysics (COMSOL Inc., Burlington, MA, USA) with the Laminar Flow (spf) physics interface. The model was set up to represent flow of water (modelled as incompressible and Newtonian fluid) through the P-FLO system. COMSOL solves the governing equations for laminar flow, consisting of the Navier–Stokes momentum equation and the continuity equation, expressed as:

$$\rho \left(u\cdot \nabla \right)u=\nabla \cdot \left[-{pI}+\mu \left(\nabla u+{\left(\nabla u\right)}^{T}\right)\right]+F,$$

$$\nabla \cdot u=0$$

where *ρ* is the fluid density, *u* is the fluid velocity vector, *p* is the fluid static pressure, *μ* is the fluid dynamic viscosity, and *F* represents external body forces such as gravity. The equations were solved under steady-state conditions using the finite element method (FEM). Appropriate boundary conditions, including specified inlet mass flow rate values, no-slip wall conditions, boundary layers, and outlet atmospheric pressure, were applied. The dependency of the numerical solution on the mesh element size was also evaluated to identify the most appropriate meshing scheme.

### Infection in the P-FLO/3D-UHU model

Fully differentiated bladder microtissue models from day 16 were used for the infection. *E. coli* UTI89-GFP (LB + supplemented with 50 μg mL^−1^ of kanamycin), COM2-mCherry or EC3-mCherry (LB+ supplemented with 100 μg mL^−1^ of spectinomycin) were cultured in static conditions for 2 days (~48 h)^33^. The microtissue models were then infected with *E. coli* UTI89-GFP, COM2-mCherry, or EC3-mCherry (5.0 × 10^6^ bacterial cells mL^−1^), and the PDMS plugs were inserted into each Transwell, followed by the placement of a custom lid over the 12-well plate, created to feature 2 mm holes to fit the inlet and outlet PTFE tubing (Darwin Microfluidics). 20 mL syringes loaded with pooled urine were connected to the plugs’ inlets and placed into the multi-channel syringe pump. The outlets were connected to a waste reservoir, and the full system was placed into an incubator at 37 °C with 5% CO_2_. A low flow rate was used to mimic the shear stress of the filling phase (0.02 mL min^−1^) for 6 h, interrupted every 2 h with a higher flow rate (0.7 mL min^−1^) for 1 min to mimic the shear stress characteristic of the voiding phase, i.e., micturition. At the end of the experiment, the plugs were carefully removed, and the apical fluid was collected for analysis (see below); afterwards, two washes with PBS were performed, and microtissues were either lysed for CFU enumeration or fixed for imaging (see below). Three independent experiments were performed.

### Antimicrobial efficacy in the 3D-UHU under dynamic (flow) conditions

Fully differentiated 3D-UHU models from day 16 were inoculated with *E. coli* UTI89-GFP (5.0 × 10^6^ bacterial cells mL^−1^). The bacterial inoculum was prepared as stated above. Immediately after, the P-FLO system was connected. Infection was allowed to take place for 12 h at 37 °C with 5% CO_2_ under a flow rate (0.02 mL min^−1^) mimicking a filling phase. After 12 h, syringes were replaced with others containing pooled human urine either untreated, or containing antibiotics (nitrofurantoin, 256 μg mL^−1^), phage cocktail (LCPR1, 1 × 10^9^ phage mL^−1^) or the combination of both treatments (256 μg mL^−1^ and 1 × 10^9^ phage mL^−1^). During treatment, a low flow rate was used to mimic the bladder filling phase (0.02 mL min^−1^) for 8 h, interrupted every 2 h with a voiding phase at the higher flow rate (0.7 mL min^−1^) for 1 min in each cycle. After plug removal, the fluid in the apical side was collected for CFU enumeration; the microtissues were washed twice with PBS, then lysed or fixed for imaging. Two microtissues per condition were used, and three independent experiments were performed.

### Determination of bacterial coverage after antimicrobial therapies

To determine bacterial coverage area, maximum-intensity projections of the GFP channel, corresponding to *E. coli* UTI89-GFP, were generated from confocal *z*-stacks acquired using a Nikon Ti2 confocal microscope. Images acquired at 40× magnification were used to quantify biofilm coverage area following treatment with nitrofurantoin, 256 μg mL^−1^, phage cocktail (LCPR1, 1 × 10^9^ phage mL^−1^), or the combination of both treatments (256 μg mL^−1^ and 1 × 10^9^ phage mL^−1^) under static and flow conditions. Segmentation of bacteria-containing regions was performed using the Labkit^85^ plugin (FIJI, built-in) with manually trained pixel classifiers to generate binary masks. The resulting masks were binarized in FIJI, and the bacterial coverage area was quantified as the proportion of GFP-positive pixels relative to the total image area. Three biological replicates (6 images per biological replicate) were analysed independently.

### Lactate dehydrogenase (LDH) cytotoxicity assay

Cytotoxicity in the bladder microtissue model was assessed by quantification of the amount of lactate dehydrogenase (LDH) released into the apical compartment, using the commercially available CyQUANT^TM^ LDH Cytotoxicity Assay (Thermo Fisher). Calculation of percentage cytotoxicity was performed as recommended by the manufacturer. Models fully lysed with 1% Triton X-100 were used as a maximum LDH control. Fluorescence intensity was measured in a Tecan Spark® 10 M plate reader (*λ*_ex_ = 490 nm and *λ*_em_ = 680 nm). Three independent experiments were performed, including three technical replicates per experiment.

### Transepithelial electrical resistance

Transepithelial electrical resistance (TEER) of 3D-UHU was measured using the EVOM3 with an STX4 electrode (World Precision Instruments, UK). Measurements were taken according to the manufacturer’s instructions. A custom 3D-printed holder was used to ensure the electrode was placed correctly in the Transwell insert^86^. Blank readings were taken from an empty 12 mm, 0.4 μm pore polycarbonate membrane plastic insert (Corning) with 900 μL of CnT Prime Epithelial 3D Airlift Medium (CELLnTEC) in the apical compartment and 1.5 mL in the basolateral compartment. Post infection, 3D-UHU models were washed twice with PBS, and pre-warmed CnT Prime Epithelial 3D Airlift Medium was added in both apical and basolateral compartments (900 μL and 1.5 mL, respectively) before TEER measurements were taken. Three biological replicates were performed.

### Resazurin assay for bacterial metabolic activity

Bacterial metabolic activity was quantified using a resazurin reduction assay. Planktonic bacteria were collected from the apical compartment of bladder microtissues at 6 h post-infection (hpi) under flow and static conditions, alongside uninfected controls. Samples were aliquoted into 96-well plates (100 μL per well). Resazurin solution (10 μL; final concentration 50 μg mL⁻¹) was added to each well, and plates were incubated at 37 °C in the dark for 1 h to allow reduction to resorufin. Fluorescence intensity was measured using a Tecan Spark® 10 M plate reader (*λ*_ex_ = 560 nm, *λ*_em_ = 600 nm). Four independent experiments were performed, each comprising two technical replicates per condition.

### Immunofluorescence staining and microscopy

Samples were fixed in 4% methanol-free formaldehyde (Fisher Scientific) in PBS and incubated at 4 °C overnight. Then, the fixative solution was aspirated out, replaced with PBS, and stored at 4 °C until staining. Sample membranes were cut out of the Transwells with a scalpel and placed in a 24-well plate with 500 μL PBS. Samples were then permeabilised with 0.2% Triton X-100 away from light for 35 min at room temperature with rocking at 15 rpm. Samples were washed twice with PBS and blocked with 5% normal goat serum (NGS, Thermo Fisher) and 1% bovine serum albumin (BSA, Merck) in PBS for 1 h at room temperature with rocking at 15 rpm, washed once with 1% BSA, and primary antibodies were then added to each sample. Primary antibodies used were rabbit anti-cytokeratin 20 (PA5-22125, Invitrogen UK) diluted at 1:75 (11.73 μg mL^−1^) and mouse anti-uroplakin 3A (sc-166808, Santa Cruz) diluted at 1:50 (4 μg mL^−1^), both resuspended in 1% NGS with 1% BSA. Samples were incubated statically overnight at 4 °C. Then, the samples were washed three times with 1% BSA. Secondary antibodies were then added: goat anti-mouse 488 (Invitrogen) diluted to 1:300 (6.67 μg mL^−1^) and goat anti-rabbit 555 (Invitrogen) diluted to 1:200 (10 μg mL^−1^) in 1% BSA, respectively, and incubated at room temperature for 2 h with rocking at 15 rpm. Samples were washed three times with PBS and incubated with phalloidin-647 (Invitrogen) diluted at 1:500 (from stock solution of 1 unit μL^−1^) in PBS for 3 h at room temperature with rocking at 15 rpm. 4’,6-diamidino-2-phenylindole (DAPI, Invitrogen) was added to the samples with phalloidin at 1:500 (2 μg mL^−1^) dilution and incubated in the same conditions for 30 min. Samples were washed 4 times with PBS and mounted onto glass microscope slides using ProLong™ Glass Antifade mounting media (Invitrogen) and covered with a cover slip. Slides were stored in a dark, dry place before imaging. Image visualization was performed by confocal laser scanning microscopy using an inverted microscope (Eclipse Ti, Nikon Inc, USA), equipped with a Digital Sight 10 microscope camera (Nikon) and X-cite LED for fluorescence imaging, using the 40× air-magnification lens and 60 × 1.4 NA oil-immersion magnification lens. The microscope was controlled by NIS-Elements v.3.30.02 software.

### Cytokine and chemokine quantification

Urine media from the apical compartments were collected from each bladder microtissue model following infection and centrifuged at 10,000 × *g* for 5 min to remove bacteria and cellular debris. The resulting supernatants were then used to quantify cytokine and chemokine production post-infection using a human premixed, customized multi-analyte Luminex bead-based immunoassay kit (R&D Systems) using the Bio-Plex 200 System according to the manufacturer’s instructions. Two technical replicates were used per condition, and three biological experiments were performed.

### Scanning electron microscopy

Samples were fixed in 2.5% (v/v) glutaraldehyde in 0.1 M phosphate buffer (for 30 min at room temperature). They were then washed twice with 0.1 M of phosphate buffer, cut out of Transwells with a scalpel, and placed in a 24-well plate. Membranes were then dehydrated in a graded ethanol (Sigma-Aldrich) series: 10 min of incubation each in 30, 50, 70, and 90% and 10 min in 100% ethanol, twice. Dehydrated microtissues were completely dried using hexamethyldisilazane (HMDS, ThermoFisher). The membranes were detached from the plastic inserts using a scalpel, mounted onto aluminium stubs using conductive carbon tape, and sputter-coated with 7 nm of gold. SEM secondary electron (SE2) images were acquired at 2 kV with a Zeiss Sigma 300 Field Emission Gun Scanning Electron Microscope (FEG-SEM) using Zeiss SmartSEM v7.02. False colouring of the images was performed using GIMP 2.10 software.

### Kinetics of bacterial cell elongation and surface coverage under static and dynamic (flow) conditions

Confocal *z*-stacks of the GFP channel (*E. coli* UTI89-GFP) were acquired from the apical surface of the urothelium using a Nikon Ti2 confocal microscope with a 60× oil objective at 2, 4, and 6 h under dynamic or static conditions. To analyse bacterial morphology, 3D images were processed in Ilastik^87^ using a supervised random forest-based machine-learning pixel classifier to generate segmentation masks of individual bacteria. The model training was performed on a representative 2048 × 2048 pixel image; the model was trained to exclude poorly segmented regions and objects not corresponding to a single bacterium. Then, the Ilastik model was applied in batch to the rest of the images of each dataset. The resulting 3D masks were imported into FIJI^88^ and analysed with a custom-based FIJI macro which employed the MorphoLibJ^89^ plugin to extract geodesic diameter (i.e., a measure of cell length). The image processing pipeline is fully available online. Three biological replicates (3 images per biological replicate) were analysed independently. The same images were used to quantify the bacterial coverage area at each time point using the pipeline described above in the bacterial coverage area section. Additionally, coverage area analysis was performed on confocal *z*-stacks of the mCherry channel (*E. coli* COM2-mCherry and EC3-mCherry) acquired using a Nikon Ti2 confocal microscope with a 60× oil objective at 6 hpi under flow or static conditions.

### Length analysis of planktonic bacterial populations

*E. coli* UTI89-GFP, COM2-mCherry and EC3-mCherry planktonic populations recovered from apical urine of infected 3D-UHU cultures at 6 hpi were assessed for bacterial cell length using a Quantom Tx Microbial Cell Counter (Logos Biosystems, Republic of Korea) according to the manufacturer’s instructions. Three biological replicates (10 images per biological replicate) were analysed independently using the BactoMeasure3D pipeline. The model was trained on a representative image with 1280 × 960 pixels.

### Image analysis and quantification of intracellular bacterial communities

Infected 3D-UHU samples after antimicrobial treatment were stained as previously described in the immunofluorescence staining section. DAPI and phalloidin were added to stain nucleic acids and actin, respectively. Confocal imaging was performed on an Evident FV4000 point scanning confocal microscope, using an Evident UPLXAPO 40XO 1.4 NA objective lens, with an image size of 1024 × 1024 pixels with a pixel size of 90 nm. Images were acquired with a resonant scanner in single-trip mode with 2 x frame averaging and a pinhole diameter of 150 μm, on to SilVIR detectors. 31.8 μm Z stacks with z-spacing of 0.43 μm were acquired at 8 individual positions per condition, see supplementary table x for channel details. Acquisition was performed in Evident CellSens Fluoview software, version 3.2.1.85.

3D image analysis measuring volume of bacteria within 3D-UHU microtissues was performed using an automated pipeline created in ZEISS arivis Pro (Carl Zeiss Microscopy GmbH, https://www.zeiss.com/microscopy/en/products/software/advanced-image-analysis.html, software version 4.5), including a deep-learning segmentation model trained using ZEISS arivis Cloud (Carl Zeiss Microscopy GmbH, https://www.arivis.cloud/).

In brief, an instance model was trained to classify nuclei and bacteria, both labelled in the same channel (DAPI). Annotation was performed with 10 images in total, with images selected across all conditions. Training was carried out in the arivis Cloud platform, and the model exported to use in Zeiss arivis Pro. To segment the cells, a 5 × 5 kernel mean filter was applied to the phalloidin channel before thresholding with a simple intensity threshold. The resulting objects were size filtered to exclude small artifacts less than a volume of 100 µm^3^. The deep learning model was used to segment cell nuclei, with a size filter to remove objects less than 30 µm^3^. The Blob Finder operation was used to segment bacteria, using a diameter of 0.525 µm (20% probability threshold, 65% split sensitivity). The compartments operation was used to define a parent-child relationship to measure bacteria volume within cells.

In parallel, to confirm the presence of intracellular communities, an *E. coli* FITC-conjugated antibody (PA1-73029, Invitrogen) was used to detect only extracellular bacteria following our protocol previously described^23^. Briefly, the microtissues were incubated with anti-*E. coli* FITC-conjugated antibody at 1:50 in PBS (100 μg mL^−1^) for 6 h at room temperature, rocking at 15 rpm. Following incubation, samples were washed 3 times with PBS after which the permeabilisation and staining (phalloidin and DAPI) protocols (as described above) were carried out. Non-fluorescent bacteria (*E. coli* UTI89) or the RFP-tagged strain were used for these infections. Under confocal microscopy, the samples with FITC/RFP/DAPI signals corresponded to the extracellular communities, while the bacteria with only RFP/DAPI signals represented intracellular pathogens. Image capture was performed on a Nikon Ti2 inverted microscope equipped with a Nikon AX confocal laser scanner and 60× magnification 1.4 NA oil-immersion lens (Nikon Instruments Inc., Tokyo, Japan). This system was also equipped with a Digital Sight 10 microscope camera (Nikon) and X-cite LED for widefield fluorescence imaging. The microscope was controlled by NIS-Elements v.6.10 software.

### Statistical analysis and schematics

Data were analysed and plotted using GraphPad Prism version 10 and RStudio 2025.09.1 + 401. The statical tests performed for each data set are indicated in the corresponding Fig. legends. Diagram/schematics were created with BioRender.

### Reporting summary

Further information on research design is available in the Nature Portfolio Reporting Summary linked to this article.

## Supplementary information

Supplementary Information File

Reporting Summary

Transparent Peer Review file

## Source data

Source Data

## Data Availability

The codes generated in this study have been deposited in the UCL Research Data Repository under 10.5522/04/32920574.v1. The data generated in this study, including the statistical analysis, are provided in the Source Data file. The custom code generated for this study is available as the BactoMeasure3D image analysis pipeline at https://github.com/aaron-crowther/BactoMeasure3D/tree/publish. The computer-aided design (CAD) files for the moulds (STL format) developed for this study are publicly available through the P-FLO GitHub repository at https://github.com/Degra97/P-FLO. Source data are provided with this paper.
