# Supplementary material for: Effect of human urinary microenvironment and fluid flow on antibiotic and phage therapy efficacy against uropathogenic Escherichia coli
Source: Nat Commun. 2026 Sep 4;17:8974. doi: 10.1038/s41467-026-76589-y (PMC13545223; doi:10.1038/s41467-026-76589-y)
Supplement: Supplementary file 1 [file 41467_2026_76589_MOESM1_ESM.pdf]

## Supplementary Figure legends

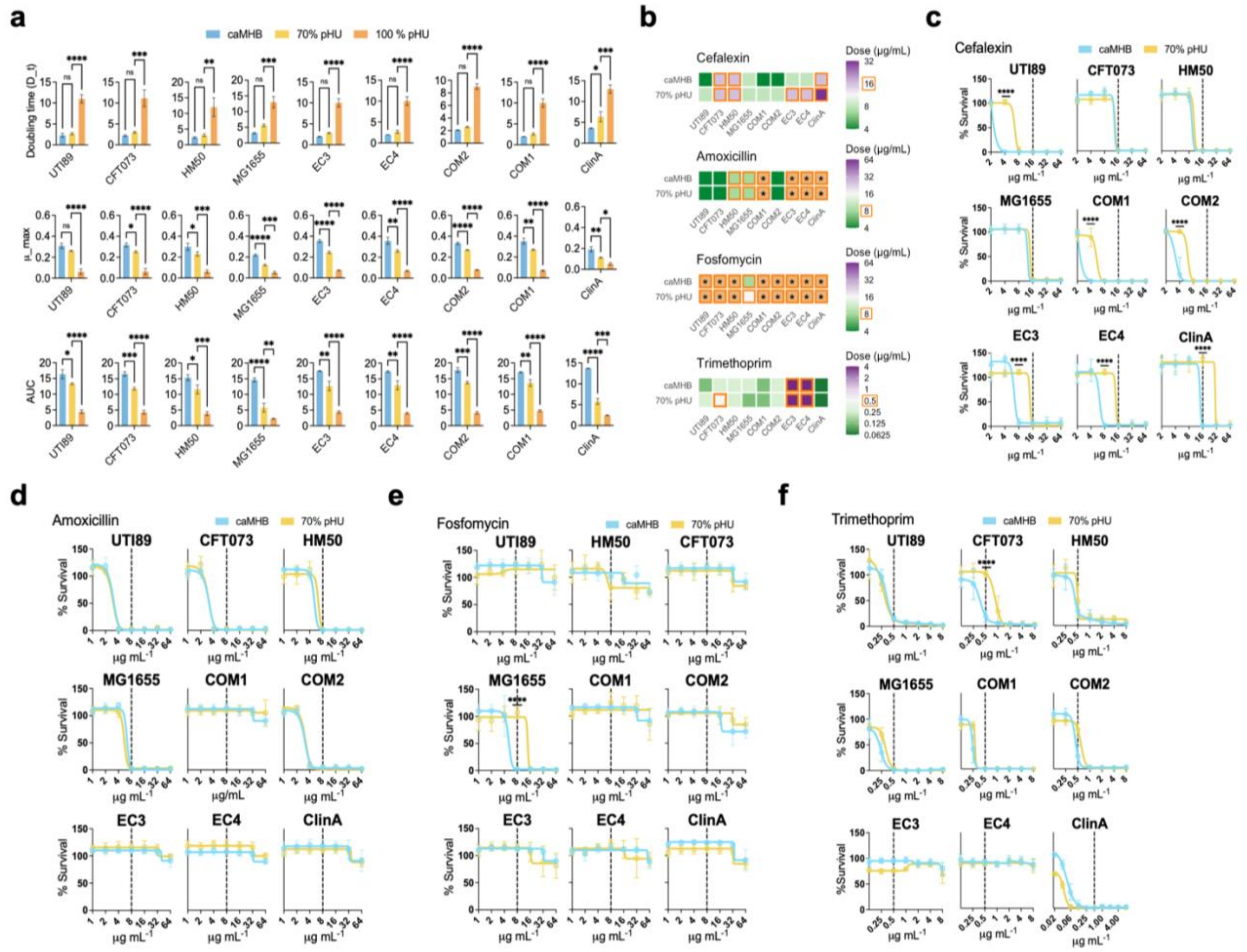


**Supplementary Fig. 1. (a)** Quantitative (non-parametric fitting) comparison of *E. coli* isolate growth parameters in caMHB (blue), 70% (yellow) and 100% (orange) pHU, including doubling time (D_t); the maximum specific growth rate (μ_max) and the area under the curve (AUC) ); standard deviation of the mean depicted. An ordinary Anova test was performed to compare the three growth microenvironments for each strain. **(b)** Heat map of the MIC results of cefalexin, amoxicillin, fosfomycin and trimethoprim, against *E. coli* strains in caMHB and 70% pHU. The orange square box defines when the MIC value sat or is above the EUCAST breakpoint, and the * means the MIC could not be determined because bacterial growth was observed at the highest concentration tested (above the EUCAST breakpoints). **(c-f)** Dose response graphs of *E. coli* strains in caMHB (blue) and 70% pHU (yellow) against cefalexin, amoxicillin, fosfomycin and trimethoprim. The dashed black line defines the EUCAST breakpoint. A t-test was performed to compare the viability at given concentrations between caMHB and 70% pHU. For all graphs the statistical significance was defined as ns, $p \geq 0.05$; *$p < 0.05$; **$p < 0.01$; ***$p < 0.001$; and ****$p < 0.0001$.

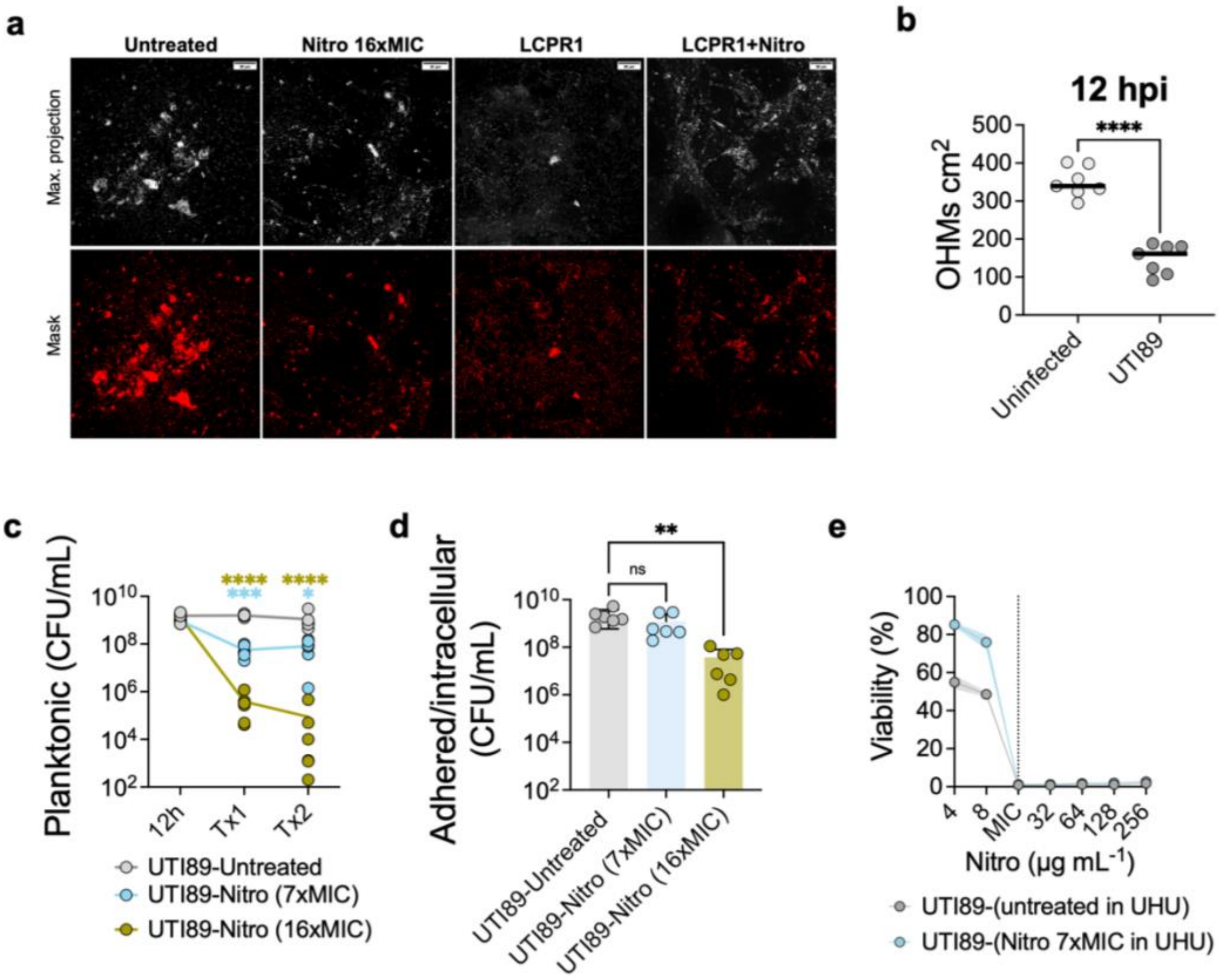


**Supplementary Fig. 2**. **(a)** Representative GFP-channel maximum z-projections of *E. coli* UTI89-GFP after antimicrobial therapy on the surface of the 3D-UHU under static conditions. The images were acquired at 40× magnification (top). Labkit-derived masks were used to calculate the percentage of bacterial coverage on the urothelial surface (bottom). The scale bar indicates 50 µm. **(b)** Epithelial barrier integrity measured by TEER on the bladder microtissue infected with UTI89-GFP 12 hpi (prior to antimicrobial therapy). An ordinary Anova test was performed to compare the different groups. **(c)** Bacterial load by CFU enumeration in the apical side (planktonic) after 12h infection, post-4h 1st treatment (Tx1) and post-4h 2nd treatment (Tx2). A Kruskal-Wallis test was performed after Tx1 and Tx2 to compare nitrofurantoin (7xMIC) with the untreated control. **(d)** Bacterial load by CFU enumeration of the attached/intracellular bacteria after the two consecutive treatments of nitrofurantoin (7xMIC). A Kruskal-Wallis test was performed to compare the treatments with the untreated control. **(e)** Dose response against nitrofurantoin in caMHB against bacterial populations recovered from the urothelium post-infection (untreated or exposed to 7xMIC). For all graphs in this Fig. , circles represent technical replicates from three biological experiments; lines indicate mean values and the error bar indicate the standard deviation between the three biological replicates where shown and statistical significance was defined as ns, $p \geq 0.05$; *$p < 0.05$; **$p < 0.01$; ***$p < 0.001$; and ****$p < 0.0001$.

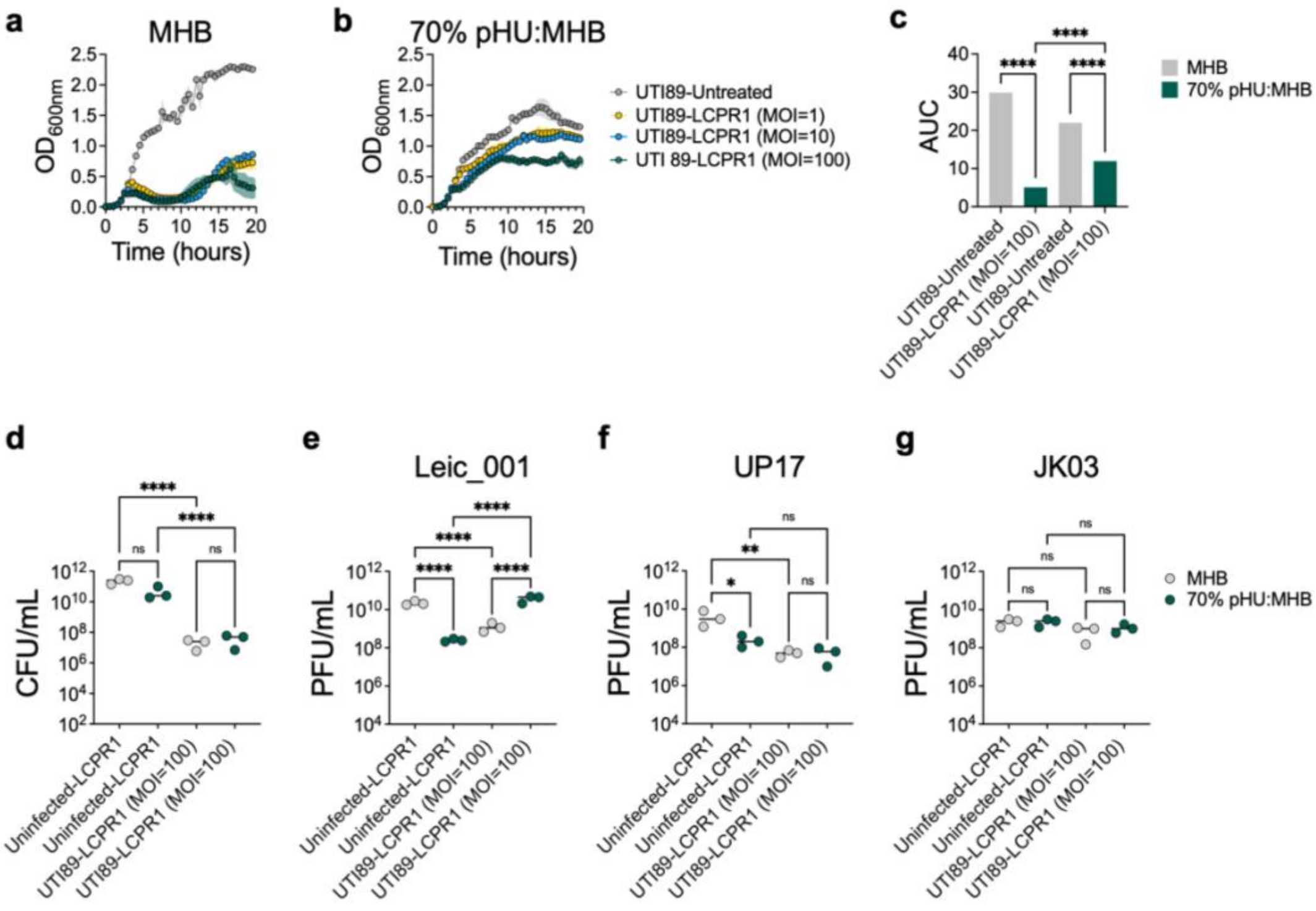


**Supplementary Fig. 3. (a)** Growth kinetics of *E. coli* UTI89 monitored by $OD_{600}$ over 20 h exposed to LCPR1 (MOI 1, 10 and 100) in MHB and **(b)** in 70% pHU:MHB. The shadowed colour represents the standard deviation between the three biological replicates, and the circles represent the mean at given timepoint. **(c)** Area under the curve (AUC) analysis to compare growth kinetics of *E. coli* UTI89 exposed to LCPR1 (MOI=100) in MHB and 70% pHU:MHB. An ordinary Anova test was performed to compare the two growth microenvironments. **(d)** CFU enumeration of *E. coli* UTI89 after 24 h exposure to LCPR1 (MOI=100) in MHB and 70% pHU:MHB . **(e-g)** PFU enumeration of Leic001, UP17 and jk03 (*E. coli* phages from LCPR1) after co-infection with *E. coli* UTI89 for 24 h at MOI100, 10 and 100 in MHB and 70% pHU:MHB. All data sets expect AUC were log-transformed to meet assumptions of normality, and differences between groups were assessed by ANOVA test. For all graphs in this Fig. , circles represent technical replicates from three biological experiments; lines indicate mean values and the error bar indicate the standard deviation between the three biological replicates where shown and statistical significance was defined as ns, $p \geq 0.05$; *$p < 0.05$; **$p < 0.01$; ***$p < 0.001$; and ****$p < 0.0001$.

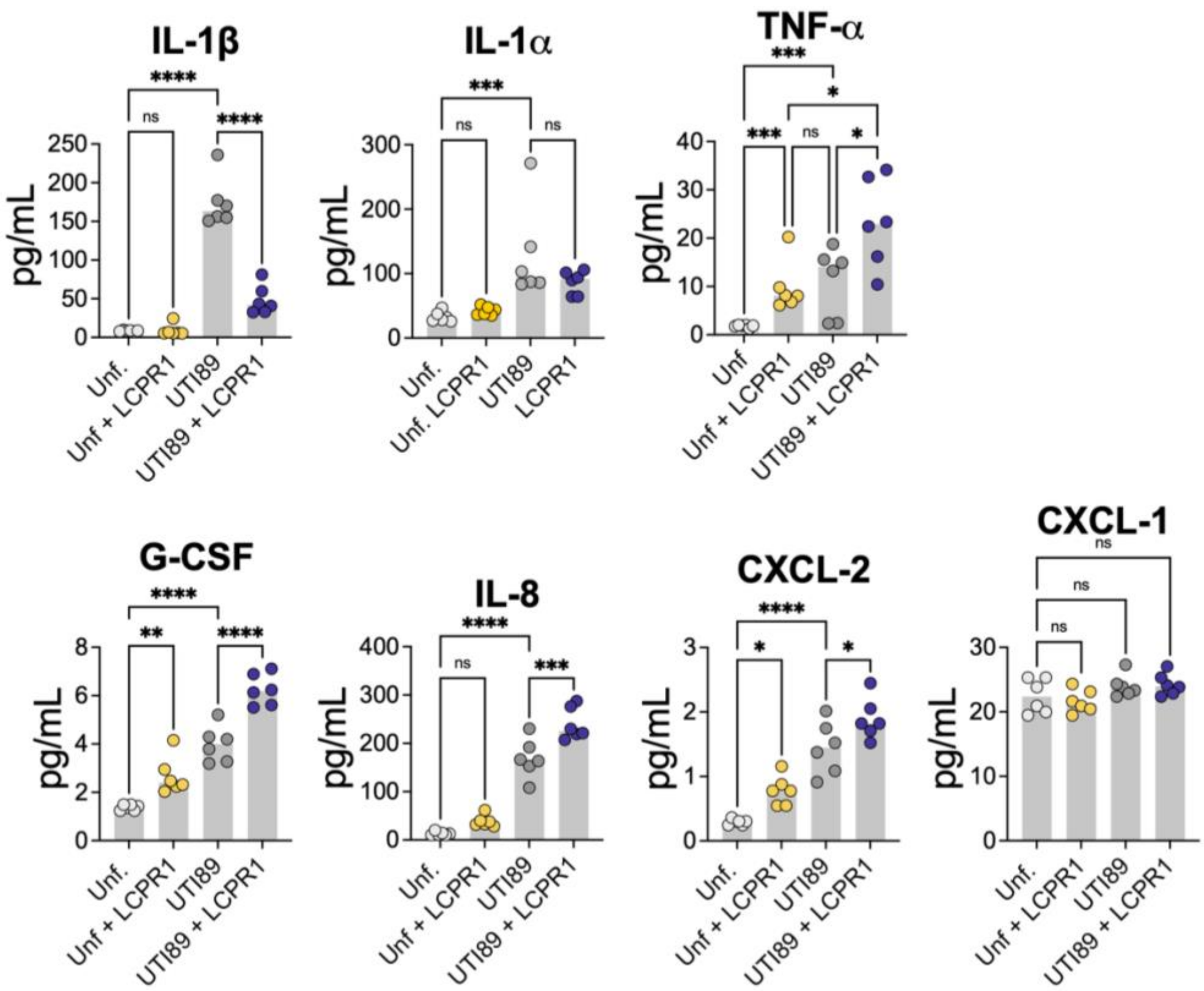


**Supplementary Fig. 4**. Cytokine and chemokine production by bladder microtissue model either uninfected; exposed to LCPR1 in the absence of bacteria; and infected with UTI89 and two sequential LCPR1 treatments (20 hpi). In all graphs, circles represent the technical replicates from three biological experiments. Differences between groups were assessed by ANOVA test and statistical significance was defined as ns, p ≥ 0.05; *p < 0.05; **p < 0.01; ***p < 0.001; and ****p < 0.000.

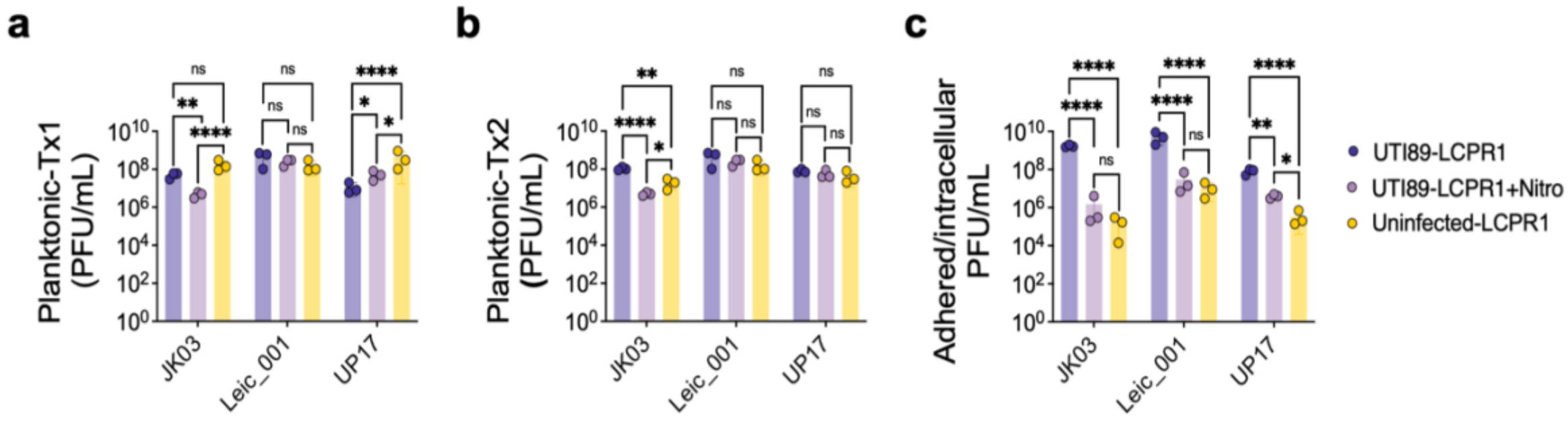


**Supplementary Fig. 5. (a)** PFU enumeration of LCPR1 (UP17, Leic001 and JK03) 20 hpi in the apical compartment of the uninfected 3D-UHU microtissue model or infected with *E. coli* UTI89 after Tx1, **(b)** Tx2 treatment and **(c)** collected from the 3D-UHU microtissue lysed at 20phi. Uninfected 3D-UHU models exposed to LCPR1 were used as controls. All data were log-transformed to meet assumptions of normality, and differences between groups were assessed by ANOVA test. Statistical significance was defined as ns, p ≥ 0.05; *p < 0.05; **p < 0.01; ***p < 0.001; and ****p < 0.000.

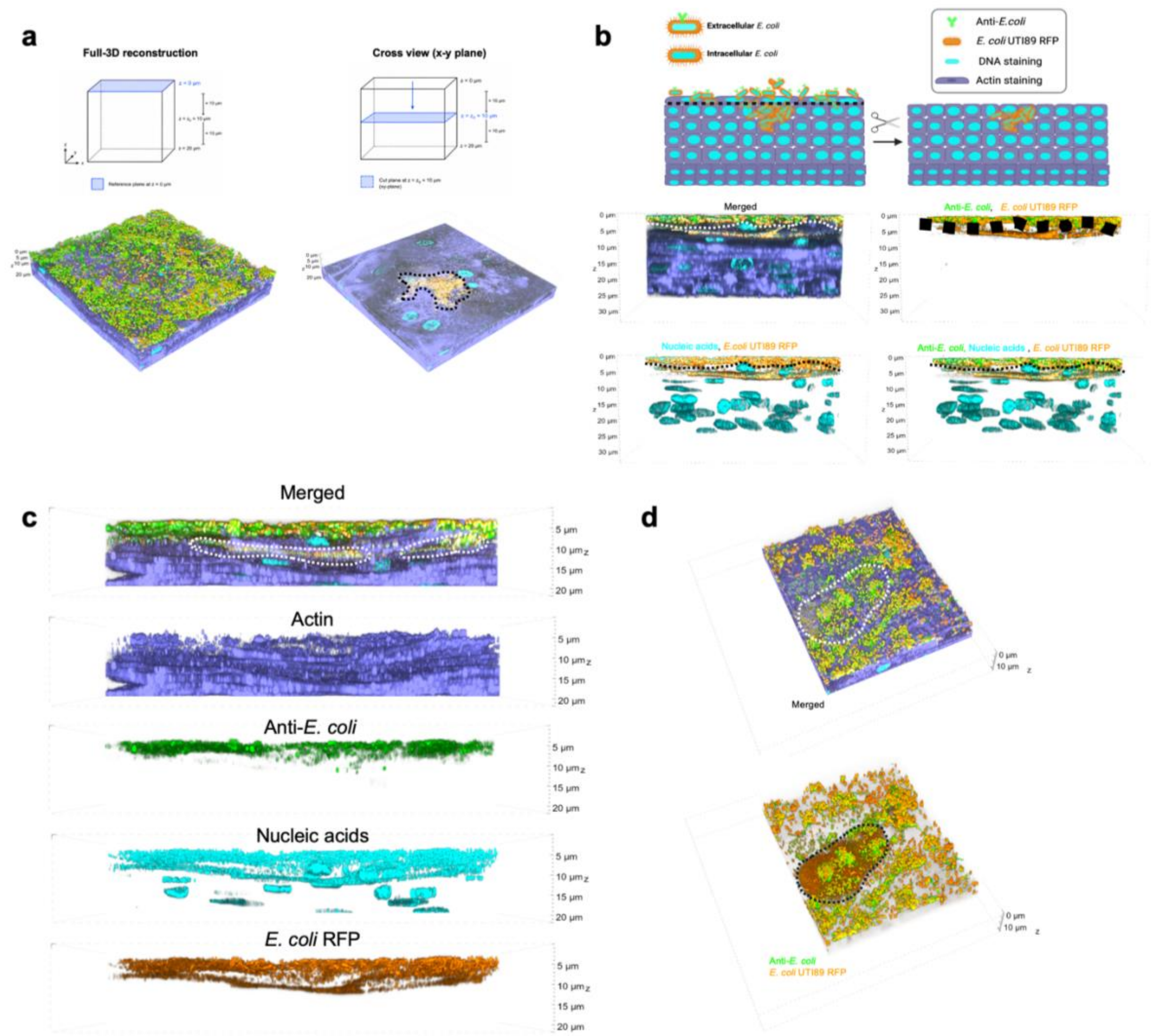


**Supplementary Fig. 6**. **(a)** 3D confocal rendering of 3D-UHU infected with *E. coli* UTI89-RFP at 20 hpi showing the full reconstructed volume (left) and a cropped cross-sectional view extracted 10 µm below the apical surface along the z-axis (right). Dashed outlines indicate IBCs. **(b)** Schematic representation of the staining strategy used to distinguish intracellular and extracellular *E. coli* populations in infected 3D-UHU (top). Extracellular bacteria are labeled by both anti-*E. coli* antibody staining (green) and bacterial RFP expression (orange), whereas intracellular bacteria are detected only by bacterial RFP expression because they are inaccessible to antibody staining. Orthogonal cross-sections of 3D-UHU infected with *E. coli* UTI89-RFP at 20 hpi are shown below as merged and split fluorescence channels. **(c)** Additional representative orthogonal cross-sections of 3D-UHU infected with *E. coli* UTI89-RFP at 20 hpi shown as merged and individual fluorescence channels. Dashed outlines indicate representative IBCs. **(d)** Additional 3D confocal renderings of 3D-UHU infected with *E. coli* UTI89-RFP at 20 hpi shown as merged channels (top) and without the F-actin channel (bottom) to facilitate visualization of intracellular bacterial communities embedded within the tissue. In all panels, bacterial RFP fluorescence (orange) identifies *E. coli* UTI89-RFP, anti-*E. coli* antibody staining (green) labels extracellular bacteria, nucleic acid staining is shown in cyan, and host-cell F-actin is shown in purple. Intracellular bacteria are defined by the absence of anti-*E. coli* antibody staining.

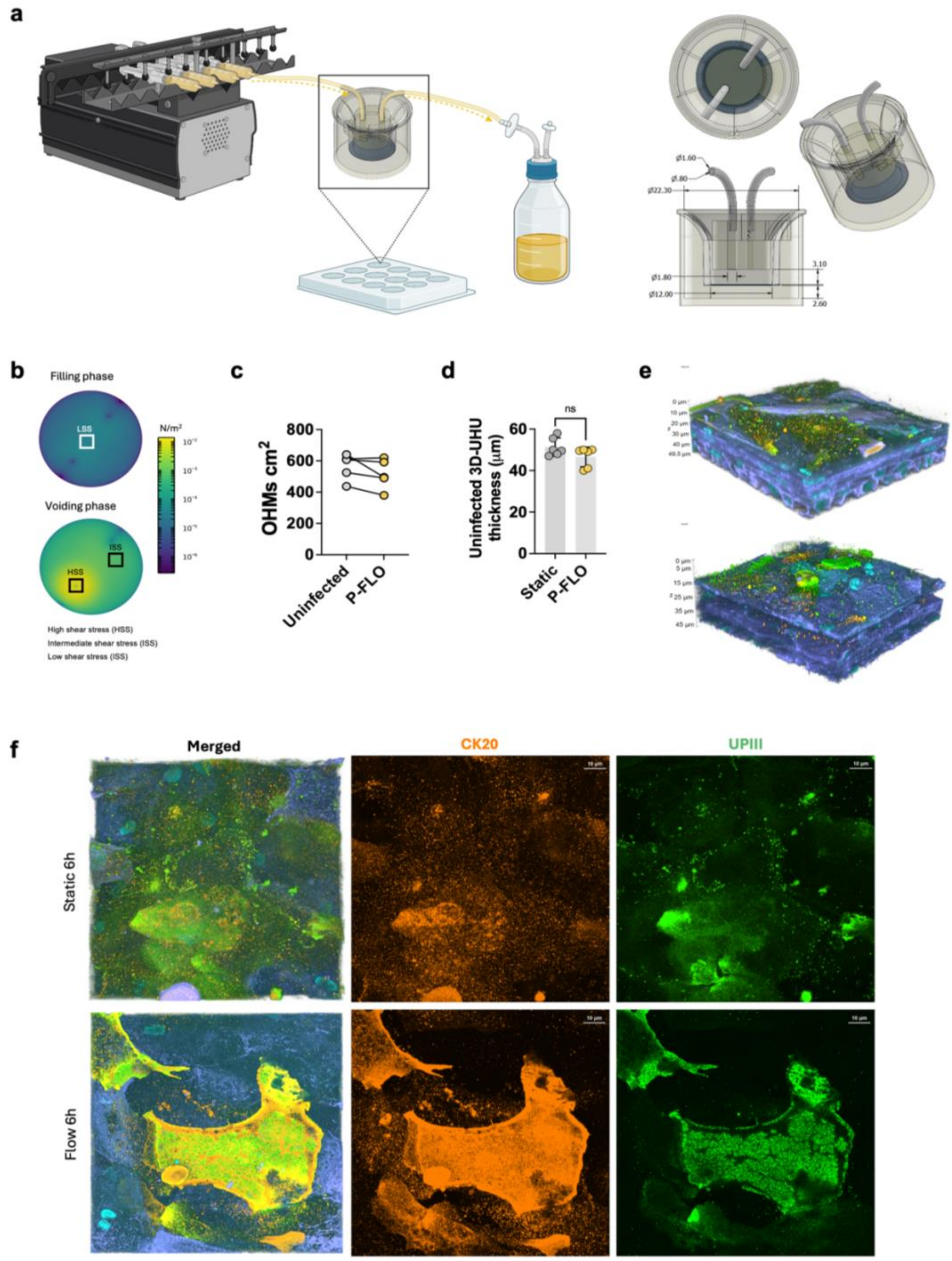


**Supplementary Fig. 7**. **(a)** Schematic representation of the P-FLO system. The setup comprises a multi-syringe pump connected to a Transwell insert within in a 12-well plate, with downstream flow directed to a waste reservoir. **(b)** Numerical simulation of the wall shear stress field in the filling phase (0.02 mL min-1) and the voiding phase (0.7 mL min-1), expressed in N $m^{-2}$. In the square boxes, areas of the urothelium experiencing different wall shear stress levels are indicated as high shear stress (HSS), intermediate shear stress (ISS), and low shear stress (LSS). **(c)** Epithelial barrier integrity measured by TEER in uninfected, fully differentiated bladder microtissue models pre- and post-insertion into the P-FLO system. At least three biological experiments were performed; each circle represents a biological replicate. **(d)** Height measurements from confocal images of uninfected 3D-UHU microtissues under static or flow conditions. A Welch's t test was performed to compare the static vs flow group. **(e)** Representative 3D rendering of confocal microscopy images of uninfected 3D-UHU in static (top) and flow (bottom) conditions. **(f)** Representative top-view confocal microscopy images of 3D-UHU under static and flow conditions, stained with urothelial differentiation markers CK20 (orange) and UPIII (green); nucleic acid (cyan); F-actin (purple). The scale bar indicates 10 µm. .Statistical significance was defined as ns, $p \geq 0.05$; *$p < 0.05$; **$p < 0.01$; ***$p < 0.001$; and ****$p < 0.000$.

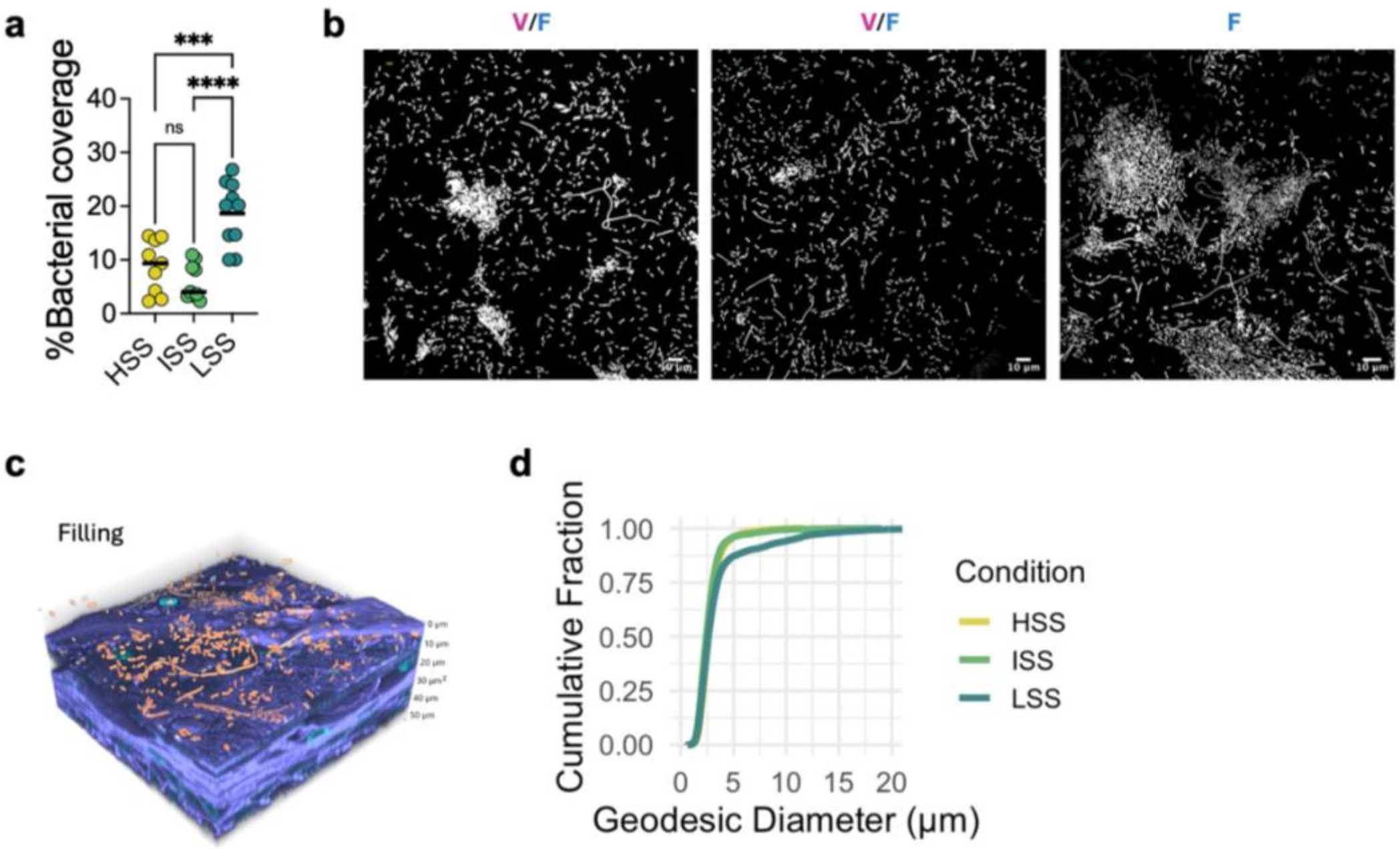


**Supplementary Fig. 8. (a)** Percentage bacterial area coverage of the urothelial surface in the HSS, ISS and LSS regions obtained from confocal imaging analysis. Data are plotted as means (line) and each circle represents a technical replicate (different images) from the three biological experiments. An ordinary Anova test was performed to compare the different groups. **(b)** Representative maximum projection confocal images showing *E. coli* UTI89-GFP signal obtained in the HSS, ISS and LSS regions. The scale bar indicates 10 µm. **(c)** Representative 3D rendering of confocal microscopy image of *E. coli* UTI89-GFP 6 hpi in the 3D-UHU using the filling protocol (6 h exposure to 0.02 mL min$^{-1}$ flow). **(d)** Cumulative fraction plot of the bacterial length obtained under filling-only and voiding/cycle regimens and grouped into HSS, ISS and LSS. Statistical significance was defined as ns, $p \geq 0.05$; *$p < 0.05$; **$p < 0.01$; ***$p < 0.001$; and ****$p < 0.000$.

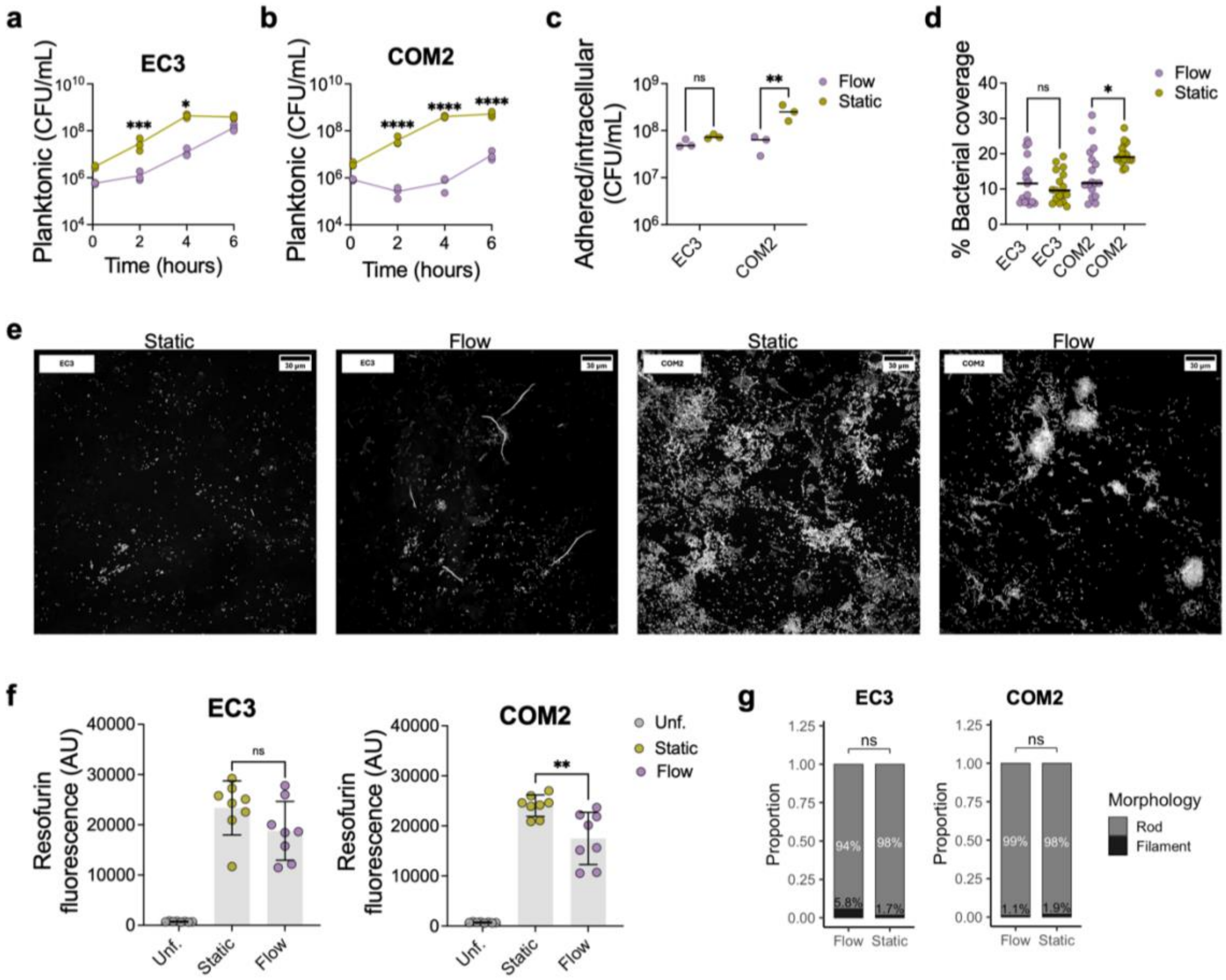


**Supplementary Fig. 9 (a)** CFU enumeration of *E. coli* EC3-mCherry and **(b)** COM2-mCherry on the apical side (planktonic) and **(c)** attached/intracellular population associated with the bladder urothelium (t=0, after each voiding event, and t= 6 h) under static and flow conditions. The data was log-transformed to fit normality and an Anova test was performed to compare the groups. **(d)** Bacterial area coverage of the urothelial surface obtained from confocal imaging under static and flow dynamic conditions (6 hpi) of *E. coli* EC3 and COM2. An ordinary Anova test was performed to compare the different groups. **(e)** Representative maximum z-projections of *E. coli* EC3-mCherry or COM2-mCherry (6hpi) under static and flow conditions on the surface of the 3D-UHU. The scale bar indicates 30 µm **(f)** Metabolic activity was quantified using a resazurin reduction assay. *E. coli* EC3 or COM2 were obtained from the apical compartment of infected bladder microtissue model (6hpi) under fluid or static conditions. A Welch's t test was performed to compare the static vs flow group. **(g)** Length analysis of *E. coli* EC3 or COM2 obtained from the apical compartment of infected bladder microtissue model (6hpi) under fluid or static conditions. Rods were classified < 5 µm and filaments > 5 µm. The proportion between rods and filaments was calculated and a Wilcoxon test was performed to compare the morphology proportions in the static vs flow conditions. For all graphs in this Fig. , circles represent technical replicates from three biological experiments; lines indicate mean values and the error bar indicate the standard deviation between the three biological replicates where shown and statistical significance was defined as ns, $p \geq 0.05$; $^{*}p < 0.05$; $^{**}p < 0.01$; $^{***}p < 0.001$; and $^{****}p < 0.0001$.

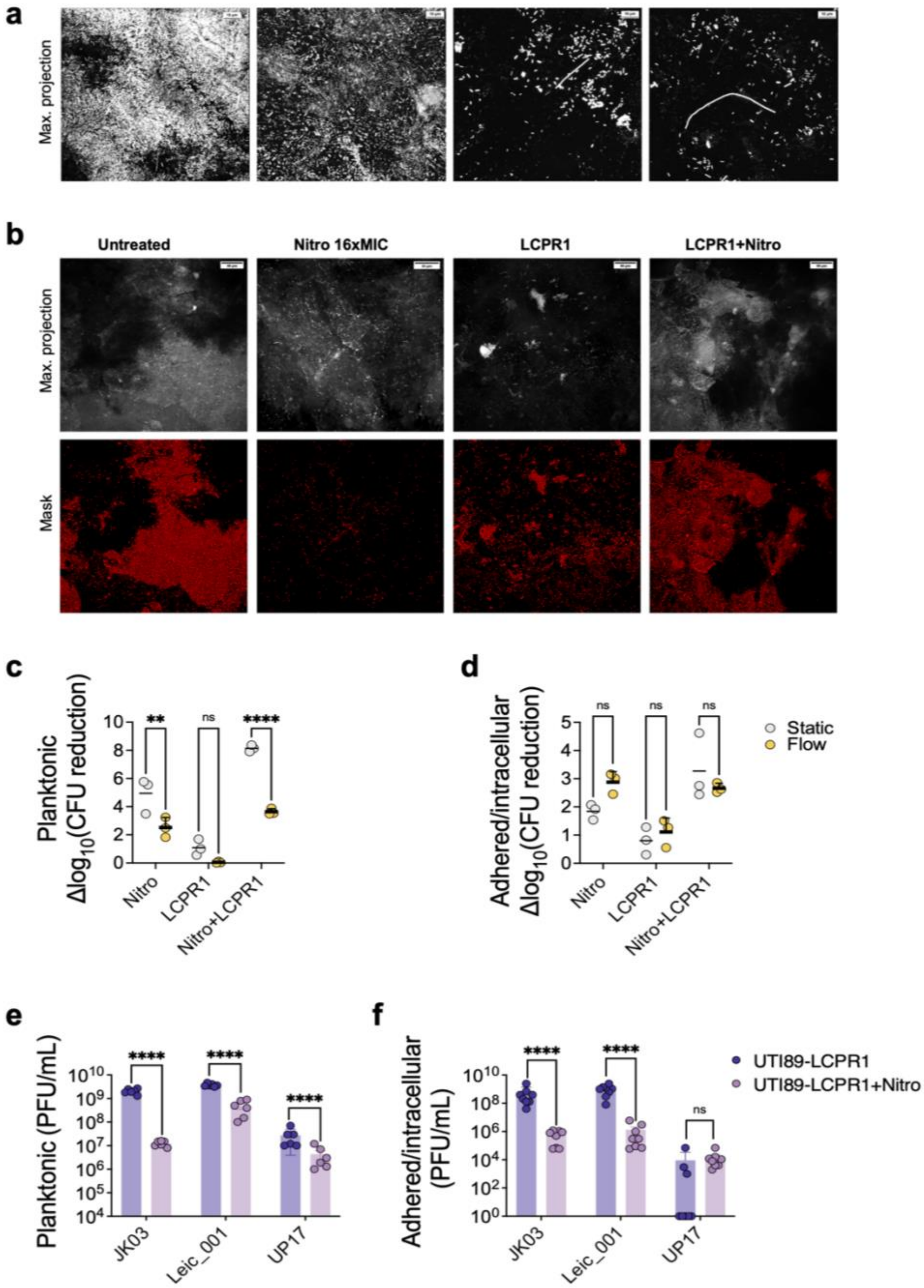


**Supplementary Fig. 10**. **(a)** Maximum z-projections of *E. coli* UTI89-GFP in the 3D-UHU under dynamic flow conditions in regions with high bacteria coverage and low bacterial coverage (left-right). **(b)** Representative GFP-channel maximum z-projections of *E. coli* UTI89-GFP after antimicrobial therapy on the surface of the 3D-UHU under flow conditions. The images were acquired at 40× magnification (top). Labkit-derived masks were used to calculate the percentage of bacterial coverage on the urothelial surface (bottom). **(c)** Comparison of the log reduction caused by antimicrobial treatments against the planktonic bacteria and **(d)** the urothelium-associated bacterial population, under static and flow conditions. Differences between groups were assessed by ANOVA test. **(e)** PCU enumeration of LCPR1 (UP17, Leic_001 and JK03) in the apical compartment of the 3D-UHU model infected *E. coli* UTI89 20 hpi under flow and **(f)** collected from the lysed microtissue comportment. The phage recovery data were log-transformed to meet assumptions of normality, and differences between groups were assessed by Anova. For all graphs in this Fig. , circles represent technical replicates from three biological experiments; lines indicate mean values and the error bar indicate the standard deviation between the three biological replicates where shown and statistical significance was defined as ns, $p \geq 0.05$; *$p < 0.05$; **$p < 0.01$; ***$p < 0.001$; and ****$p < 0.0001$.
