# Supplementary material for: Effect of human urinary microenvironment and fluid flow on antibiotic and phage therapy efficacy against uropathogenic Escherichia coli
Source: Nat Commun. 2026 Sep 4;17:8974. doi: 10.1038/s41467-026-76589-y (PMC13545223; doi:10.1038/s41467-026-76589-y)
Supplement: Supplementary file 3 [file 41467_2026_76589_MOESM3_ESM.pdf]

nature portfolio

Peer Review File

Effect of human urinary microenvironment and fluid flow on antibiotic and phage therapy efficacy against uropathogenic *Escherichia coli*

Corresponding Author: Professor Jennifer Rohn

This file contains all reviewer reports in order by version, followed by all author rebuttals in order by version.

Version 0:

Reviewer comments:

Reviewer #1

(Remarks to the Author)
This manuscript addresses a critical gap in UTI research, which is the discrepancy between standard in vitro antibiotic susceptibility testing and clinical treatment failure. The authors introduce a novel, low-cost "mesofluidic" culture system (P-FLO) compatible with standard Transwell inserts to introduce physiological flow dynamics to a 3D human urothelial microtissue model (3D-UHU). The strengths of this study are that: 1) Innovation: the P-FLO system represents a technical innovation. 2) Conditions: the authors rigorously compare standard media/conditions (MHB, static) vs. physiological ones (human urine, 3D tissue, and flow dynamics). This is critical because many treatments are tested in not physiological conditions. This will allow effectively isolates the variables contributing to treatment failure. 3) Diverse strain collection: the authors used a variety of relevant E. coli strains including uropathogenic and asymptomatic isolates as well as laboratory strains.
By using the P-FLO platform, the authors evaluated the efficacy of nitrofurantoin and a bacteriophage cocktail (LCPR1) against UPEC. The main findings were: First, antibiotic and phage efficacy are significantly reduced in human urine and the 3D tissue environment compared to standard nutrient-rich broth. This is extremely relevant because in the clinic the treatment failure is high and it contributes to antimicrobial resistance. Second, fluid flow conditions (which is physiological relevant) induced bacterial filamentation, a UPEC phenotype that facilitates immune evasion, persistence, and the infection of neighboring cells. These adaptive responses likely contribute to the pathogenesis of recurrent UTIs by enabling the bacteria to survive standard treatments. They also found that host-like conditions limit phage efficacy compared to lab media. This study this model can help to test treatment in more relevant conditions instead of relaying in lab conditions, including rich culture media. Importantly, the authors acknowledged the limitations of this models (e.g. extravasation of immune cells). This manuscript presents valuable insights for the UTI field, and the majority of the results strongly support the authors' conclusions. However, there are some issues to address before its publication.

Major comments:

1. Statistical rigor: No statistical analyses were performed in Fig. 1A, Fig. 1C, and corresponding supplementary figures.
2. Phage Cocktail Composition: It would be important to discuss the rationale behind the selection of the specific phage cocktail, which containing a vast majority of Klebsiella phages and three E. coli phages.
3. Bacterial Growth Conditions: Type 1 pili are essential for establishing infection. To induce maximal pilus expression, strains should be grown statically for 18–24 hours at 37°C, followed by a 1:1,000 subculture into fresh media for a subsequent 18–24 hours static incubation. (PMC4488945; PMC4488945; PMC10159856). However, the Methods section states that cultures were grown statically for 48 hours. It is unclear if this involved a subculturing step. If cultures were maintained for 48 hours without nutrient replenishment, the resulting prolonged stationary phase could significantly compromise cell viability and alter cell wall architecture, potentially masking phage receptors. Furthermore, this condition introduces a methodological inconsistency, as other assays in the study utilized overnight shaking or static cultures. Clarification is required regarding the exact growth protocol and how these distinct metabolic states might influence treatment susceptibility.

4. Quantification of IBCs: Although the Supplemental Figure 3 showed that antibiotic treatment promotes IBC formation, this observation lacks quantitative support. To validate this finding, formal quantification and statistical analysis comparing this

treatment to the non-treated, LCPR1, and LCPR1 + Nitro groups must be performed.

5. In Supplemental figure 4, why 100 % of urine was not used?

6. Please change the color of the actin channel in Figure 4, as the current choice offers poor visibility. A higher-contrast color is needed to resolve the filaments clearly.

7. A critical question arises from Figure 4: specifically, how does fluid flow impact IBC formation across the different treatment groups? Since flow was shown to induce filamentation—a phenotype often linked to evasion—it would be highly relevant to visualize and quantify IBCs under these dynamic conditions to see if they differ from the static model (Supplementary figure 3).

8. In lines 691-693, the authors mentioned that “Notably, phage UP17 showed reduced titers 691 levels both in the apical and microtissue compartment, which also reflects lower infectivity compared to the other two phages, JK03 and Leic001 (data not shown from independent experiments).” It would be important to include that data.

Reviewer #2

(Remarks to the Author)
Garcia Maset et al.

In this manuscript Garcia Maset et al. presents a technically sophisticated approach to address gaps in current understanding of UTI, in particular why antimicrobial efficacy measured under standard in vitro conditions fails to predict clinical outcomes.

The integration of human urine, a differentiated urothelial microtissue, and a novel mesofluidic flow system (P-FLO) represents a substantial methodological advance. While the main strength of this study is the addition of flow to an already established model, the work do demonstrate that microenvironmental and mechanical cues shape UTI89 behaviour and treatment response.
The manuscript could be of interest to audiences in infection biology, antimicrobial resistance, tissue engineering, and phage therapy. With a few additional experiments and extended analysis/discussion, I believe that this study could be accepted for publication.

Major Strengths:
This study’s central premise that physiological microenvironments and flow dynamics modulate antimicrobial efficacy is compelling and well justified with backing of experimental evidence. The study directly addresses the disconnect between MIC-based susceptibility testing and clinical treatment failure, which is a major unresolved issue in UTI management. The inclusion of phage therapy, both alone and in combination with antibiotics, enhances the translational relevance (while it remains to be seen if this can fully be translated for improved patient outcomes).

Adding flow to the 3D-UHU model is a clear improvement and a step towards a more realistic physiological situation, although as the authors also point out themselves some critical aspects (such as external immune cells and their corresponding responses) are still missing to call it a fully physiological model. I think that calling it “fully differentiated” is a stretch, even if CK20 can be detected).

I find the observation that nitrofurantoin efficacy diminishes under flow despite increased exposure particularly interesting (and counterintuitive). The observation that phage treatment primarily modulates host responses rather than bacterial burden in 3D tissue is also novel and potentially important.

Areas for improvement:
While MICs of platinic bacteria are a good measure for concentrations they survive in (in liquid culture), these values do not generally correlate to survival rates intracellularly. One alternative would be to follow antibiotic concentrations reported in urine from patients (PMID: 20231390 and refs therein). I think this should be explored.

Related to the above point, while the data look solid, parts of figure 1 (A and B) requires better justification as the results are not followed up on for the reminder of the study, thus it is somewhat difficult to understand the relevance of it. Alternatively, the results for Nitro could presented as a stand-alone or be incorporated either in Fig2 and in the supple.
Figure 2I and related text, further markers for host responses should be included, possibly IL-6, IL-8, and/or CXCL1, or markers of epithelial stress. This would strengthen claims about host modulation by phage treatment.

Initially are multiple bacterial strains examined, but as I understand it, only the response of UTI89 tested and followed up in the P-FLO model, why is that? I find this is a major limitation when claiming generalized results and conclusions for the study. One or two additional strains should be tested.

The manuscript risks overselling the efficacy of phage therapy in this system based on the following 3 points:
1, In urine, phage activity is reduced.
2, In the 3D-UHU model, LCPR1 does not significantly reduce bacterial burden, even though host modulation is observed.
3, Combination therapy does not outperform nitrofurantoin alone against adherent or intracellular bacteria.
The authors should expand on the discussion into what is currently understood about the combination therapy in-vivo.

Reviewer #3

(Remarks to the Author)
General assessment:
This manuscript describes a plug-flow–linked organoid (P-FLO) system designed to introduce controlled luminal flow and intermittent voiding-like shear stress to a differentiated human bladder epithelial model. The platform is technically interesting and addresses an important limitation of static in vitro UTI models. However, the study contains several foundational inconsistencies between simulated and experimental flow conditions, unclear volume and voiding dynamics, multiple instances where conclusions exceed the data presented and missing references to already existing studies. As a result, key physiological and mechanistic claims, particularly regarding shear stress, bacterial persistence, immunomodulation, and treatment failure, are not sufficiently supported in the current form. While the P-FLO system represents a promising technical advance, the current manuscript contains major conceptual and experimental shortcomings. Substantial clarification and additional experimental work would be required for the manuscript. In particular, inconsistencies between simulated and experimental flow conditions, limited strain variability and overinterpretation of short-term end-point data limit the strength of the conclusions.

Major concerns:

1. Inconsistencies between CFD simulations and experimental flow rates (major conceptual issue): While CFD simulations were performed at filling and voiding flow rates of 0.002 and 1.7 mL min^{-1}, respectively (line 277), the infection experiments were conducted at 0.02 and 0.7 mL min^{-1} (e.g. lines 305–306 and schematics of the P-FLO system). Since wall shear stress scales with volumetric flow rate under laminar flow conditions, the shear stresses experienced by the urothelium during infection likely differed substantially from those validated by CFD.

This discrepancy raises several additional concerns:
• If a filling flow rate of 0.02 mL min^{-1} was indeed used experimentally, the transwells in Supplementary Figure 6A and C would have received 7.2 mL of pooled human urine over 6 h, which is incompatible with the maximum supported volume of 1.5 mL specified by the supplier.
• If the CFD-derived filling rate of 0.002 mL min^{-1} is instead the correct experimental value, this corresponds to 0.24 mL per transwell over 2 h. In that case, the subsequent voiding step (0.7 mL min^{-1} or is it 1.7 mL min^{-1} for 1 min?) would completely empty the apical compartment, even when accounting for the initial inoculum volume (which is not specified in the Methods).
• It is unclear whether the apical compartment is fully voided or whether a dead volume remains, which seems likely given that the PDMS plug and tubing cannot contact the microtissue directly without causing injury. The authors should quantify this dead volume and discuss how it compares to clinically relevant post-void residual urine volumes (with up to ~100 mL considered normal in humans).

Overall, the mismatch between simulated and experimental flow conditions, combined with unclear apical volume dynamics, undermines the physiological interpretation of shear stress in the P-FLO system and must be clarified.

2. Limited strain diversity in infection experiments: Although the authors initially test multiple UPEC strains (including clinical isolates) in axenic culture, all infection experiments in the tissue model are performed exclusively with UTI89. Given that clinical isolates were already available, these should be included in the infection experiments to determine whether key phenotypes, such as filamentation under flow and discrepancies in treatment efficacy between static and flow conditions, are conserved across strains.

3. Limited antibiotic diversity in infection experiments: Given the broader claims regarding treatment failure under flow, inclusion of at least one additional clinically relevant antibiotic with a distinct mechanism of action would help establish whether the observed effects are nitrofurantoin-specific or represent a general property of the P-FLO system.

4. Overinterpretation of persistence without long-term follow-up: The authors propose bacterial persistence as a potential explanation for antibiotic and/or phage treatment failure. However, persistence is not directly demonstrated. The experimental design focuses on short-term outcomes (≤8 h of treatment) and relies exclusively on end-point measurements, without assessing regrowth after treatment removal. Recurrent UTIs in vivo are driven by long-term persistence and relapse over days to weeks, often involving quiescent intracellular bacterial communities (IBCs). Although IBCs are observed in this model, they are not followed longitudinally. Real-time imaging or delayed end-point measurements after antibiotic removal would substantially strengthen claims regarding persistence, filamentation, fluxing, and recurrence.

5. UP3 immunostaining performed with permeabilization: For immunofluorescence staining of UP3, the tissue was permeabilized. As UP3 is a membrane protein, permeabilization may artificially increase signal by staining cytosolic (immature) protein pools. UP3 staining should be performed without permeabilization to accurately reflect mature, membrane-localized expression.

6. Use of 70% pHU for antimicrobial susceptibility testing: Although the authors demonstrate that all strains can grow in 100% pooled human urine, subsequent antimicrobial susceptibility testing is performed in 70% pHU supplemented with 30% caMHB. From a physiological perspective, 100% pHU would be the most relevant medium, as reduced growth rates and altered osmolarity or pH are known to affect antibiotic efficacy (e.g. Yang et al., Urology 2014; Mickiewicz et al., Nature

Communications 2019; Fabijan et al., Bacteriology 2022; Paduthol et al., BioRxiv 2025). Additionally, MHB contains starch, which can bind toxins released upon antibiotic treatment. Even 30% caMHB may therefore buffer bacterial stress responses, potentially confounding susceptibility results. If 70% pHU is retained, this choice requires stronger justification and authors should avoid phrasing it as physiological relevant. Figure 1B should also explicitly state "70% pHU" rather than "pHU," as the current labeling is misleading.

7. Claims of IBC survival after nitrofurantoin exposure lack regrowth evidence: The authors state that IBCs survive nitrofurantoin exposure and may contribute to treatment failure (line 506). However, no regrowth of tissue-associated bacteria after antibiotic removal is demonstrated. A recent microfluidic bladder model showed such regrowth directly (Paduthol et al., BioRixv 2025). The authors should either demonstrate regrowth in their own system or temper their conclusions accordingly and acknowledge prior work.

8. Insufficient evidence for immunomodulatory effects of LCPR1: The claim that LCPR1 attenuates host inflammatory responses and reduces tissue damage (line 558) is based solely on IL-1β secretion. This is insufficient to support broad immunomodulatory conclusions. Additional markers, such as NF-κB activation, TNFα, interferons, or barrier integrity readouts (e.g. tight junction staining or TEER measurements), are needed.

9. Barrier integrity under flow and reduced tissue thickness: Despite claims that flow does not alter barrier integrity (line 588), tissues exposed to 6 h of flow appear markedly thinner (Supplementary Figure 5D) compared to static controls (Supplementary Figure 5C). The authors should comment on this observation and assess whether repeated filling/voiding leads to epithelial exfoliation (e.g. by analyzing effluent for shed cells) or cell death (e.g. by DRAQ7 stainings). In addition, Supplementary Figure 5 contains inconsistent flow-rate values (0.002 vs 0.02 mL min^{-1} for filling; 0.7 vs 1.7 mL min^{-1} for voiding), mirroring discrepancies in the main text. These must be corrected consistently across the manuscript. The markedly reduced TEER values in the static, uninfected condition (~500 $\Omega \cdot cm^2$) compared to the original 3D-UHU model (~1000 $\Omega \cdot cm^2$; Jafari & Rohn, 2023) also require verification and explanation. Does insertion of the PDMS plug compromise tissue integrity?

10. Filamentation under flow: The authors need to cite prior work already demonstrating UPEC filamentation under flow-induced mechanical stress (e.g. Andersen et al., Infection and Immunity, 2012) – which limits the novelty of the presented work. It remains unclear whether the observed filaments arise from intracellular bacteria fluxing out of infected cells or represent an extracellular phenotype. Earlier and later time points, as well as live imaging, would help clarify filament formation dynamics and reversibility.

11. Discrepancies between representative images and quantitative coverage data: In Figure 4, images under flow conditions (4B, 4E) appear to show near-complete surface coverage, whereas quantitative analysis reports ~22% coverage (Figure 4J). A similar discrepancy exists for static infection (Figure 2E vs 2J). The authors should ensure that representative images accurately reflect the quantified data or verify their Fiji analysis with manual validation.

12. Viability of adherent bacteria inferred solely from CFU counts: The conclusion that adherent bacteria under flow are non-viable after treatment is based exclusively on CFU enumeration. Given the mechanical and antibiotic stresses involved, bacteria may enter a transiently non-culturable state by standard plating techniques. Additional viability or metabolic assays (e.g. live/dead staining, resazurin assays) and recovery experiments after treatment removal are needed to support claims regarding bacterial viability.

Minor concerns:

• Lines 77-83: Claims regarding poor translatability of existing UTI models should be softened, as the current model also relies on a single immortalized cell line and usage of a laboratory-adapted UPEC strain only. Additional UTI models should be cited (e.g. Sharma et al., 2021; Horsley et al., 2018; Paduthol et al., 2025).
• Line 36: Clarify that LCPR1 urine experiments refer to axenic cultures.
• Lines 85, 109, 858–859: The term "organoid" is misleading for a transwell-based models, even if for other studies those were derived from dissociated spheroids. The system presented should be consistently described as a transwell-based bladder model. This also applies for the nomenclature of the Plug-Flow Linked Organoid (P-FLO) system; it should be named Plug-Flow Linked Transwell system or Plug-Flow Linked 3D-UHU system.
• Line 91: Remove the term "personalized," as no patient-derived tissues are used.
• Lines 94-97: Statements regarding treatment failure across pathogens require citations.
• Line 225: Change "in LB agar plates" to "on LB agar plates."
• Line 229: Avoid the term "biofilm-like" without evidence of biofilm structure.
• Figure 2 timeline discrepancy (12 h vs 14 h) should be resolved.
• Lines 515, 525, 715, 722, 729, 759, 774, 779: Various overinterpretations, missing citations, and terminology issues should be corrected as detailed in the upper comments.
• Supplementary Figures: Correct typographical errors (ISS vs LSS; PFU vs PFC).

Version 1:

Reviewer comments:

Reviewer #1

(Remarks to the Author)
The authors have comprehensively addressed all of my concerns and appropriately acknowledged the limitations of the study.

Reviewer #2

(Remarks to the Author)
I commend the authors for significantly strengthen the paper through this round of reviews, and wish to congratulate them on a beautiful piece of work! I'd recommend accept.

Reviewer #3

(Remarks to the Author)
The authors took great consideration of my comments and performed a substantial amount of additional experiments, analyses, and clarification/re-writing of the existing text. The manuscript improved considerably, especially with the addition of the immunomodulatory data and the time-resolved analysis of filamentation under flow. I particularly appreciated seeing that the authors discovered additional features of their system by performing experiments suggested by the different reviewers.

However, I still feel that some comments could be addressed further, as detailed below:

1. The authors have substantially clarified the discrepancies between CFD-simulated and experimentally applied flow conditions and corrected the incorrect flow values throughout the manuscript. The clarification that the P-FLO system represents a continuously perfused flow-through system designed to modulate wall shear stress, rather than physically filling and emptying the apical compartment, resolves several of my original concerns regarding Transwell volume capacity and complete voiding.
However, I still believe the manuscript somewhat overstates the physiological mimicry of bladder filling and voiding. The current system appears to reproduce temporal changes in shear stress rather than the broader mechanical and volumetric dynamics of true bladder filling/micturition cycles (e.g. stretch, pressure changes, urine accumulation, etc.). I would therefore encourage the authors to slightly moderate this terminology throughout the manuscript for conceptual precision.
Additionally, further clarification regarding the broad range of shear stress values reported (0.017–17 Pa; with 17 Pa more closely reflecting vascular-level shear stress than values typically associated with the bladder) together with clarification of the effective apical chamber volume and potential dead-volume/reservoir effects within the plug/tubing system would further strengthen the technical characterization of the platform.

2. The inclusion of additional clinical isolates substantially strengthens the manuscript and addresses my original concern regarding strain specificity of the observed flow-associated phenotypes. In particular, the observation that the pathogenic isolate EC3 displays filamentation under flow conditions whereas the asymptomatic isolate COM2 does not is informative. However, while the authors now demonstrate broader applicability of the infection phenotypes, the generalizability of the reported treatment-response differences between static and flow conditions across diverse UPEC strains remains less explored and could be acknowledged as a limitation.

3. The authors have acknowledged the limitation of using only one antibiotic in the study while focusing on phage therapy as a second treatment modality, either alone or in combination with antibiotics, and have now appropriately included this limitation in the Discussion.

4. I thank the authors for clarifying their use of the term persistence, specifically that they refer to phenotypic antibiotic persistence rather than long-term persistence in the context of recurrence.

5. The authors have now performed additional analyses of bacterial growth kinetics, re-run MIC assays in 100% pooled human urine (pHU), and more clearly explained their rationale for using 70% pHU supplemented with caMHB. However, their statement in lines 152–154 and 167–168 regarding the MICs observed in 100% pHU could still be misleading, as they state that the lower MICs observed in 100% pHU “may reflect the reduced bacterial growth”.
While strongly reduced growth in pHU may indeed make MIC determination technically more challenging (e.g. distinguishing growth from no-growth conditions), the current phrasing could be misinterpreted as implying that reduced growth directly causes lower MICs. Mechanistically, slow or impaired bacterial growth often renders bacteria less susceptible to many antibiotic classes, which would instead be expected to increase MIC values due to phenotypic tolerance. I would therefore advise the authors to revisit the wording of this sentence to avoid misleading non-expert readers.

6. Additional experiments, including measurements of released cytokines, were performed to address the immunomodulatory aspects of the study. Together with the TEER measurements, this new dataset highlights an interesting aspect of the LCPR1 phage cocktail on tissue immunity. I genuinely enjoyed seeing this part added to the manuscript.

7. The authors explain the difference in TEER values between the presented data and their initial 3D-UHU model from 2023 by the use of different electrodes, which is a very reasonable explanation. However, they now also mention slightly different culture conditions between the two set-ups (the Jafari paper and the current manuscript). Could the authors comment on why

they modified the previously published protocol (e.g. regarding seeding density and timing of pHU addition, as far as I can see)?
Additionally, is the passage number mentioned in the Methods section inclusive of the passages performed by the supplier? If so, the authors should more accurately reflect this in the manuscript by correcting the stated passage number, as was done in the original 3D-UHU publication.

8. The newly added temporal analyses of filamentation substantially strengthen the manuscript and improve mechanistic interpretation of flow-associated filamentation! The comparison with previous flow-based studies (Andersen et al.) is now appropriately contextualized.

9. The authors have now added additional lower-magnification images in Figure 4, and the clarification regarding image selection substantially improves concordance between representative imaging and quantitative coverage analysis.

Additionally, I have a minor readability suggestion: in several revised figures (e.g. Fig. 4C, 4D, and 4G), the order of presentation switches from static->flow to flow->static, whereas other panels (e.g. Fig. 4F) retain the original ordering of static->flow. Although the labels are correct, maintaining a consistent condition order throughout the manuscript figures would improve readability and facilitate comparison across datasets.

Open Access This Peer Review File is licensed under a Creative Commons Attribution 4.0 International License, which permits use, sharing, adaptation, distribution and reproduction in any medium or format, as long as you give appropriate credit to the original author(s) and the source, provide a link to the Creative Commons license, and indicate if changes were made.
In cases where reviewers are anonymous, credit should be given to 'Anonymous Referee' and the source.
The images or other third party material in this Peer Review File are included in the article's Creative Commons license, unless indicated otherwise in a credit line to the material. If material is not included in the article's Creative Commons license and your intended use is not permitted by statutory regulation or exceeds the permitted use, you will need to obtain permission directly from the copyright holder.
To view a copy of this license, visit https://creativecommons.org/licenses/by/4.0/

We thank the editor and the referees for the insightful and useful comments about our manuscript. We are happy that all three referees highlighted many positive attributes of our originally submitted work.

The paper, they said, addresses a critical gap in the UTI field (R1, R2), including the "major unresolved issue in UTI management" represented by the disconnect between standard susceptibility testing and antibiotic failure in the clinic (R2). All three referees noted that the P-FLO system embodied an innovative technical advance, addressing an important limitation in static models and representing a step towards more realistic physiological conditions. R2 praises our "technically sophisticated approach" and central premise, noting that our claim that "physiological microenvironments and flow dynamics modulate antimicrobial efficacy is compelling and well justified with backing of experimental evidence"; R1 agrees that our findings that the urinary tract microenvironment affect treatment response is rigorously performed, "extremely relevant" to the clinical reality and represent "valuable insights". As far as broad audience appeal, R2 notes that our work could be of interest to "audiences in infection biology, antimicrobial resistance, tissue engineering, and phage therapy" with "translational relevance".

All three referees raised interesting, important issues and flagged up areas where revised language or enhanced data could bolster and improve our work. We are very grateful for their careful scrutiny, and below we have outlined how we have addressed all their comments, including the addition of a substantial number of new experiments and analyses. We are delighted with the revised manuscript and think that the paper is vastly improved because of the peer review process. Referee comments are in black, whereas our responses are in blue.

Reviewer 1.

1. Statistical rigor: No statistical analyses were performed in Fig. 1A, Fig. 1C, and corresponding supplementary Fig..

We have now performed new statistical analyses, described in the methodology under the "bacterial growth curves" section, as follows: "QurvE[30] in RStudio was used to analyse growth curve data using a non-parametric fitting" (Line 772-773). The resulting analysis (doubling time, maximum growth, and area under the curve) can be found in **Supplementary Fig. 1A**.

In the Results section, we added the following statement to incorporate this new analysis: "First, we assessed bacterial growth in various media. In addition to the gold-standard growth media (caMHB) recommended by The European Committee on Antimicrobial Susceptibility Testing (EUCAST) guidelines for MIC, we included 70% pooled human urine (pHU) (in 30% caMHB) to mimic a UTI infection microenvironment. Such a 70% urine mix was previously used to determine drug susceptibility while still supporting adequate bacterial growth[42]. Growth in 100% pooled human urine (pHU) was markedly impaired (**Fig. 1A**), with prolonged doubling times and substantially reduced maximal growth relative to 70% pHU (**Supplementary Fig. 1A**). Although bacterial growth in 70% pHU remained lower than in caMHB, consistent with the reduced nutrient availability of urine-based media, growth kinetics more closely resembled those observed in caMHB (**Fig. 1A**), including comparable doubling times and area under the curve (AUC), with strain-dependent differences (**Supplementary Fig. 1A**). Together, these results indicated that 70% pHU provided a more suitable balance between physiological relevance and bacterial growth support for subsequent antimicrobial assays" (Line 132-144)

Additionally, Fig. 1 has been updated based on other referee's feedback and only nitrofurantoin's in vitro antimicrobial activity is now shown in **Fig 1**. The updated **Fig. 1C** (dose response for nitrofurantoin) and Supplementary **Fig. 1C-F** (rest of antibiotics) now includes

the statistical analysis (t test) comparing bacterial viability at given concentrations in the different media tested.

2. Phage Cocktail Composition: It would be important to discuss the rationale behind the selection of the specific phage cocktail, which containing a vast majority of Klebsiella phages and three E. coli phages.

We apologize that we were not clearer on this important point. The following comment was added in the Methodology section: "The optimised bacteriophage cocktail LCPR1 has been developed for another project as a broader-spectrum therapy for UTI caused by *E. coli* or *Klebsiella pneumoniae*" (Line 156-157). Similarly, in the Results section: "The bacteriophage cocktail (LCPR1) was previously optimised in a separate project and comprises three *E. coli* phages and six *K. pneumonia* phages. It was developed using a broad panel of antibiotic-resistant urinary clinical isolates collected within the UK. The inclusion of multiple phages is a known strategy to broaden the host range and improve the phage cocktail coverage[53–55]." (Line 238-242)

3. Bacterial Growth Conditions: Type 1 pili are essential for establishing infection. To induce maximal pilus expression, strains should be grown statically for 18–24 hours at 37°C, followed by a 1:1,000 subculture into fresh media for a subsequent 18–24 hours static incubation. (PMC4488945; PMC4488945; PMC10159856). However, the Methods section states that cultures were grown statically for 48 hours. It is unclear if this involved a subculturing step. If cultures were maintained for 48 hours without nutrient replenishment, the resulting prolonged stationary phase could significantly compromise cell viability and alter cell wall architecture, potentially masking phage receptors. Furthermore, this condition introduces a methodological inconsistency, as other assays in the study utilized overnight shaking or static cultures. Clarification is required regarding the exact growth protocol and how these distinct metabolic states might influence treatment susceptibility.

Thank you for this observation. We followed the protocol of Mulvey et al 2001 (the citation has been added in the Methodology section), which other UTI papers have also used and cited (e.g. 10.1111/j.1462-5822.2006.00691; doi 10.1093/infdis/jix023). We wished to generate data comparable to our previous publication (Flores et al. 2023), where *E. coli* isolates were also grown in the same conditions described by Mulvey et al.; in Flores et al., the type-1-piliated phenotype can clearly be observed in the SEM and confocal images; haemagglutination assays in this paper also confirmed the presence of the adhesin. The overnight cultures were diluted in 100% pHU (normally 1:1000 dilution to adjust for the correct MOI) and the infection was conducted for 12h in the microtissue model, during which the bacteria would have fully adapted to their new urinary environment. Bacterial growth and viability within the microtissue model after this method is robust, with all expected bacterial virulence behaviours (including strong adhesion) and host responses occurring (Flores et al. 2023). Therefore, we used this same protocol for the microtissue model infection (updated including the new strains tested as requested by the Referee 2 and 3).

The following clarification of method has been added to the Methodology section.

".*E. coli* UTI89-GFP (LB + supplemented with 50 µg mL^{-1} of kanamycin), COM2-mCherry or EC3-mCherry (LB+ supplemented with 100 µg mL^{-1} of spectinomycin) were cultured in static conditions for 2 days (~48h) as described previously[34]" (Line 840-841)

For the rest of in vitro assays that involved antimicrobial testing, we used a single overnight culture in shaking conditions following the guidelines for antimicrobial testing by EUCAST. These assays therefore followed standardized conditions recommended for susceptibility

testing, whereas the 48-hour static culture conditions were specifically applied only to the microtissue infection experiments (to induce maximal pilus expression).

4. Quantification of IBCs: Although the Supplemental Fig. 3 showed that antibiotic treatment promotes IBC formation, this observation lacks quantitative support. To validate this finding, formal quantification and statistical analysis comparing this treatment to the non-treated, LCPR1, and LCPR1 + Nitro groups must be performed.

We apologise for the confusion here. In the original manuscript in **Supplementary Fig. 3**, we visualised IBC formation in the untreated controls and also in the nitrofurantoin treatment group. We hypothesised that IBC formation was a possible cause of treatment failure. It was not our intention to suggest that treatment promoted IBC formation.

We agree IBC quantification is a great addition to the manuscript. We have now developed a new imaging pipeline to quantify IBC. The requested extra data have been added into **Fig. 2 (I-J)**, and the pipeline analysis can be found in the "Image analysis and quantification of intracellular bacterial communities" section in the Methods. These new data are exciting because they show that in static conditions, while the phage cocktail can reduce IBC, nitrofurantoin at the normal clinical dose cannot, a result that greatly strengthens the paper.

5. In Supplemental Fig. 4, why 100 % of urine was not used?

Thank you for this comment. The fundamental issue with the MIC is that good bacterial growth is needed for the readout, yet 100% human urine did not support optimal enough growth of *E. coli* strains. However, we have now added MIC/MBC testing against nitrofurantoin in 100% pHU (Fig. 1B-C), and as expected, we observed lower MIC values, likely due to the poor growth in that medium. In the Results section, we added the following statement:

"Growth in 100% pooled human urine (pHU) was markedly impaired (**Fig. 1A**), with prolonged doubling times and substantially reduced maximal growth relative to 70% pHU (**Supplementary Fig. 1A**). Although bacterial growth in 70% pHU remained lower than in caMHB, consistent with the reduced nutrient availability of urine-based media, growth kinetics more closely resembled those observed in caMHB (**Fig. 1A**), including comparable doubling times and area under the curve (AUC), with strain-dependent differences (**Supplementary Fig. 1A**). Together, these results indicated that 70% pHU provided a more suitable balance between physiological relevance and bacterial growth support for subsequent antimicrobial assays." (Line 136-144)

Because bacterial growth is essential for phage replication, we evaluated the efficacy of LCPR1 against UTI89 in caMHB and in 70% pHU:30 MHB. The original Supplementary Fig. 4 has been updated now (renamed **Supplementary Fig. 3**), with additional analyses of the growth curves (AUC) and statistical comparisons of CFUs and PFUs in the two different microenvironments tested. The following statement was added to the Result section:

"The growth kinetics of UTI89 was monitored under exposure to LCPR1 using different MOI (phage to bacteria) in two different media (MHB and 70% pHU:30% MHB). 100% pHU was not included because UTI89 exhibited insufficient growth in urine alone (**Supplementary Fig. 1A**) to support reliable assessment of phage replication and antibacterial activity." (Line 244-247)

6. Please change the color of the actin channel in Fig. 4, as the current choice offers poor visibility. A higher-contrast color is needed to resolve the filaments clearly.

We agree. A new image was added to better highlight the presence of filaments (**Fig. 4B**). However, due to the high bacterial coverage, visualization of the filaments can be challenging.

To address this, additional examples have been included **in Supplementary Fig. 10A**, showing filaments in areas of high bacterial coverage as well as selected images with lower bacterial density where the filaments are more clearly visible.

7. A critical question arises from Fig. 4: specifically, how does fluid flow impact IBC formation across the different treatment groups? Since flow was shown to induce filamentation—a phenotype often linked to evasion—it would be highly relevant to visualize and quantify IBCs under these dynamic conditions to see if they differ from the static model (Supplementary Fig. 3).

This is a great suggestion, thank you, which yielded interesting results. As mentioned in your point 4, we now have developed a new imaging pipeline to quantify IBC. We performed IBC quantification under dynamic (flow) vs static (no flow) conditions, and this is shown in the updated **Fig. 4D-E**. The Results section has been updated as follows:

"Furthermore, the bacterial invasion strategy of UTI89 previously observed in static conditions (**Fig. 2I**) was not impaired under flow conditions; indeed, a greater proportion of IBCs were observed under mechanical stress cues (**Fig. 4D-E)**, in comparison with the static model (**Supplementary Fig. 6**). Taken together, these results suggest that flow conditions promoted UTI89 attachment and intracellular invasion compared with our previously established static infection model, indicating a role for hydrodynamic forces in enhancing these virulence attributes." (Line 469-474)

8. In lines 691-693, the authors mentioned that "Notably, phage UP17 showed reduced titers 691 levels both in the apical and microtissue compartment, which also reflects lower infectivity compared to the other two phages, JK03 and Leic001 (data not shown from independent experiments)." It would be important to include that data.
I
We apologies for this. The infectivity of the individual phages was tested in the microtissue model and the data can be found in **Supplementary Fig. 5**.

The result section has been updated as follow: "Phage recovery from the 3D-UHU was also assessed in both the apical (Tx1 and Tx2) and the microtissue compartments to evaluate phage dynamics and replication (**Supplementary Fig. 5A-C**). In the apical planktonic compartments, phage recovery varied among the individual phages. JK03 showed the lowest titre under the combined treatment in both Tx1 and Tx2 (**Supplementary Fig. 5A-B**), whereas comparable titres were observed for Leic_001 and UP17 in Tx2 (**Supplementary Fig. 5B**). In the microtissue compartment (adhered/intracellular), all three phages recovered from LCPR1 alone had higher titres compared with the dual therapy group (**Supplementary Fig. 5C)**. This finding correlated with the bacterial count result, which showed that the combined treatment more effectively reduced bacterial population compared with LCPR1 alone (**Fig. 2B-C**), thereby limiting phage amplification due to reduced host availability. Nevertheless, this pattern was not observed in the apical phage recovery results, which suggests bacteria-dependent phage uptake or replication within the microtissue compartment, highlighting the complexity of phage-bacteria dynamics within the bladder microenvironment."(Line 294-306)

Reviewer 2

1. While MICs of platinic bacteria are a good measure for concentrations they survive in (in liquid culture), these values do not generally correlate to survival rates intracellularly. One alternative would be to follow antibiotic concentrations reported in urine from patients (PMID: 20231390 and refs therein). I think this should be explored.

We apologise for the confusion here. In fact, to mimic clinical treatment as closely as possible, we had deliberately chosen a concentration that is agreed to be a consensus real-world

concentration for nitrofurantoin at steady state in the human bladder. The following statement was added to clarify this in the manuscript: “Firstly, we determined the efficacy of nitrofurantoin at a clinically relevant concentration, consistent with reported human urinary pharmacokinetics[44] (16xMIC, 256 mg mL^{-1}).”(Line 206-207)

2. Related to the above point, while the data look solid, parts of Fig. 1 (A and B) requires better justification as the results are not followed up on for the reminder of the study, thus it is somewhat difficult to understand the relevance of it. Alternatively, the results for Nitro could presented as a stand-alone or be incorporated either in Fig2 and in the supple.

Thank you for this comment; we agree that our point was not getting across. Conceptually, we wanted to ground our study by first conducting a general investigation of commonly used UTI antibiotics with multiple strains to determine whether the presence of urine in MIC assays could make a major difference. Having determined that the lack of urine in MIC was likely not a major reason for failure of MIC to predict clinical outcomes, we next wanted to narrow our further investigations using one obvious test case: nitrofurantoin, which is one of the most common first-line treatments for UTI in many countries including our own.

To make the manuscript harmonize more with this conceptual framework, we have now focused only on nitrofurantoin in **Fig. 1**, and we also performed additional MIC/MBC testing of nitrofurantoin in 100% urine (as per Referee 3’s point 6). The results of the rest of antibiotics were moved to **Supplementary Fig 1**. The following statements were added in the Results section to explain the relevance and rationale of nitrofurantoin being the main test case:

“Next, we investigated the AST profiles of nitrofurantoin, one of the first-line antibiotics recommended for acute UTI in the UK and many other countries[41].” (Line 146-147)

“We next tested other antibiotics commonly used to treat UTIs (**Supplementary Fig. 1B**).” (Line 156)

“Nitrofurantoin remains a resilient and popular therapeutic option for UTIs, due to its localised delivery, favourable pharmacokinetic and pharmacodynamic properties, and multifaceted effects on bacterial physiology[51]. Resistance acquisition is constrained by the need for multiple independent mutations and the near absence of horizontally transferable resistance mechanisms[52]. Therefore, we focused on nitrofurantoin for all subsequent steps of this study. In addition, we narrowed our efforts to strain UTI89, as it is one of the most characterised UPEC isolates.”(Line 176-181)

3. Fig. 2I and related text, further markers for host responses should be included, possibly IL-6, IL-8, and/or CXCL1, or markers of epithelial stress. This would strengthen claims about host modulation by phage treatment.

We were very happy to perform tests for additional host-response markers, and we thank the referee for this suggestion, because it yielded interesting new information. We used a Luminex panel to test additional relevant chemokines/cytokines, the results of which have been added to **Fig. 2H** and **Supplementary Fig. 4**. We updated the Methods section to describe the Luminex procedure, as previously we only used ELISA, and the following statements were added to the Results section:

“To better assess the effect of LCPR1 on urothelial function, we also measured secretion of several inflammatory cytokines and chemokines. The addition of LCPR1 alone induced modest secretion of G-CSF, known to promote neutrophil tissue residency[58]. UTI89 infection induced the secretion of pro-inflammatory cytokines and chemokines, although not CXCL1 (**Supplementary Fig 4**). Strikingly, LCPR1 co-infection with UTI89 completely abrogated IL-

1b secretion, mirroring the effects seen with cytotoxicity. This was in sharp contrast to the effects on secretion of G-CSF, the neutrophil chemoattractants CXCL2 and CXCL8, and the pro-inflammatory cytokine TNFa, all of which showed greater UTI89-induced secretion following LCPR1 co-infection (**Fig. 2H** and **Supplementary Fig. 4**). Together, these findings suggest that while LCPR1 lacks bactericidal efficacy in 3D-UHU, it may protect urothelial viability, attenuate inflammasome activation and promote neutrophil influx in the urothelial environment".(Line 274-284)

In the Discussion section, the following statements were added: "In the infected 3D-UHU model, under static (non-flow) conditions, LCPR1 alone reduced IBC formation and attenuated cellular cytotoxicity. This was accompanied by lower secretion of IL-1β, but not other cytokines tested. The coupling of LDH and IL-1β release suggests a link between cellular cytotoxicity and inflammasome activation in urothelial cells after UPEC infection. In contrast, bacteriophage co-infection augmented secretion of the pro-inflammatory cytokine TNFα alongside the neutrophil chemokines and maturation agents CXCL2, CXCL8 and G-CSF. We hypothesise that phage-mediated lysis of *E. coli* releases bacterial ligands that bind to Toll-like receptors expressed by 3D-UHU[24], signalling via NF-kB and promoting inflammation, neutrophil chemotaxis and maturation, further supporting bacterial clearance. Such a model requires testing in more complex experimental systems, and does not exclude direct host-phage interactions that may directly modulate the urothelial response to UPEC interaction[75–77], evidenced by direct induction of G-CSF following 3D-UHU exposure to LCPR1 alone." (Line 580-591)

4. Initially are multiple bacterial strains examined, but as I understand it, only the response of UTI89 tested and followed up in the P-FLO model, why is that? I find this is a major limitation when claiming generalized results and conclusions for the study. One or two additional strains should be tested.

Thank you for this comment. The referee will appreciate that the complexity and resource intensity of the 3D platform and of the P-FLO system are orders of magnitude higher than that of a simple, high-throughput MIC assay, necessitating some prioritisation. Given their status as arguably the most well-characterised uropathogens, UTI89 or CFT073 are often the sole strain tested in many seminal UTI papers, both in mouse and human-cell modelling papers such as Russel et al. (2023), Rousseau et al. (2023), Paduthol et al. (2026), Guedes et al. (2026) and many others. Having said that, we agree that testing additional strains is of value. Therefore, in the P-FLO system we added two isolates, strains EC3 (a uropathogen isolated from UTI patient urine) and COM2 (a non-pathogenic *E. coli* isolated from the urine of a healthy individual). Additionally, we investigated filamentation and adhesion of EC3 and COM2 under static and flow conditions. Moreover, filamentation and metabolic activity assays of the planktonic populations (obtained from the apical compartment of the microtissue model) were performed. In response to this comment, we have added a new **Fig. 3** and **Supplementary Fig. 9**) and additional text to both the Methods (to describe the strains) and the Results, as below:

"To determine whether our observations held true beyond UTI89, we next assessed two clinical isolates in the P-FLO system: *E. coli* EC3 (derived from urine of a UTI patient) and COM2 (derived from asymptomatic donor urine). As observed for UTI89, EC3 showed significantly fewer planktonic CFUs under flow at 2 and 4 h ($p = 0.0007$ and $p = 0.0442$), with no difference at 6 h ($p = 0.8525$) (**Supplementary Fig. 9A**). COM2 CFUs were also reduced under flow, but more severely than for EC3 ($p < 0.0001$) (**Supplementary Fig. 9B**). Under static conditions, COM2 exhibited higher urothelium-associated CFU counts than did EC3 (1-log increase), indicating enhanced urothelial attachment. Flow did not impair EC3 urothelial association, with comparable CFUs ($p = 0.8447$) and surface coverage ($p = 0.634$) to static conditions, whereas COM2 CFUs were reduced ($p = 0.024$), alongside reduced attachment ($p = 0.112$) (**Supplementary Fig. 9C–E**). Confocal imaging revealed pronounced EC3

filamentation at 6 h post-infection under filling/voiding cycles. In contrast, COM2 did not filament, but rather formed aggregates under both static and flow conditions (**Supplementary Fig. 9E**). As shown in **Supplementary Fig. 9F**, the metabolic activity of COM2 planktonic population was markedly reduced under flow in comparison with static conditions ($p = 0.0091$), similarly to UTI89 (**Fig. 3J**). However, in EC3, a similar metabolic activity was observed in both conditions ($p = 0.1273$). Additionally, we investigated the bacterial length for the planktonic population in static and flow conditions, and no statistical differences between the rod/filamentation proportions were observed for EC3 and COM2 in static versus flow conditions (**Supplementary Fig. 9G**). (Line 429-446)

5. The manuscript risks overselling the efficacy of phage therapy in this system

We apologize and agree our phrasing may have overstated the results. Although our new data strengthen the case somewhat, it is still important to be circumspect. We have therefore softened our relevant conclusions in the Discussion; see the purple phrases in the paragraph below:

"In the static 3D-UHU model, the dual treatment (i.e., LCPR1 + nitrofurantoin) achieved a total eradication of the planktonic bacteria, outperforming the individual treatments. However, while the combination treatment could not clear the overall microtissue-associated bacterial population, it did appear to recapitulate key features of both monotherapies, producing reductions in bacterial burden and attachment comparable to nitrofurantoin alone while also reducing the proportion of intracellular bacterial communities, consistent with the effect of phage treatment. These data suggest that the therapeutic benefit of the combination may derive from complementary targeting of extracellular and intracellular niches, rather than from increased overall bactericidal activity. Further studies will be required to determine how efficacious phages will ultimately prove to be for UTI treatment in the clinic." (Line 595-603)

Below we also respond to each of the subpoints of this thoughtful criticism:

based on the following 3 points:

5.1. In urine, phage activity is reduced.

The referee is absolutely correct that phage activity is reduced in urine in vitro, which was an observation we did emphasize in our original paper. On the face of it, we agree that this is not a promising feature for the use of phages to treat UTI. However, in our original experiments, the phages performed better in the more complex 3D-UHU models in combination with nitrofurantoin, and we have now performed more experiments that highlight the benefit of phages even further when they are deployed in more realistic conditions (see below points 5.2 and 5.3).

Nevertheless, to clarify the subtleties of phage/urine interactions, we have performed a few more analyses. New analysis (AUC) of the growth curves of UTI89 co-infected with LCPR1 (MOI=100) in the two media tested revealed that growth UTI89 was more reduced in standard media in comparison with the urine containing media (**Supplementary Fig 3C**). Furthermore, LCPR1 at MOI 100 reduced bacterial CFU by ~4 log in MHB and ~3 log in 70% pHU:30% MHB pHU (**Supplementary Fig. 3D-E**). Additionally, the phage titres in the uninfected conditions (without bacteria) were significantly reduced in urine containing media suggesting that phages were less stable (**Supplementary Fig 3F-G**). Together these results flesh out the observation that in urine, phages are indeed somewhat impaired.

5.2, In the 3D-UHU model, LCPR1 does not significantly reduce bacterial burden, even though host modulation is observed.

We apologise that our wording was not clear around this point. We have removed the term "host modulation" as we think it is confusing.

However, based on other referee feedback, we quantified the effect of LCPR1 on UTI89 invasion (referee #1 point 4), which showed that LCPR1 actually reduced IBCs, which shows that in this specific compartment at least, phages can significantly reduce bacterial burden.

We have therefore added this statement: "In contrast, LCPR1 treatment reduced IBCs despite having no measurable effect on CFU in the adhered/intracellular compartment (**Fig. 2C,J**), indicating a selective impact on IBC formation rather than overall bacterial burden:

As regards the host responses, and in line with this referee's point 3 above (also requested by referee #3 point 8), we tested an expanded panel of cytokine and chemokine responses elicited by LCPR1 alone in our bladder microtissue model, compared with those elicited with the LCPR1 + UTI89 combination. The data have been added to the updated Fig. 2, and in the Results section, the following statements have been added:

"Notably, LCPR1 alone attenuated cellular cytotoxicity induced by UTI89 infection ($p = 0.001$), but this effect was modest relative to the protective effect on cell viability of nitrofurantoin alone ($p < 0.0001$) (**Fig. 2F**)". (Line 272-274)

5.3, Combination therapy does not outperform nitrofurantoin alone against adherent or intracellular bacteria.

Thank you for this comment. In fact, the new intracellular invasion analysis mentioned above (**Fig. 2J**) revealed a complementary activity of the combined treatments. The following statement has been added to the Results section.

"Notably, the combined treatment of phage and antibiotic decreased both the adhered and intracellular bacterial load (**Fig. 2C, J)**, consistent with complementary effects of both treatments. Together, these findings reveal complementary activities of nitrofurantoin and LCPR1 against extracellular and intracellular UPEC reservoirs within the urothelium." (Line 330-334)

6. The authors should expand on the discussion into what is currently understood about the combination therapy in-vivo

We agree this extra context would be valuable. In the Discussion, we have added:

"Compared with the use of phage or antibiotics alone, combination therapy has been reported to provide superior efficacy in suppressing bacterial populations and curbing the evolution and spread of antimicrobial resistance[78]."(Line 593-595)

Reviewer #3

(Point one has several subpoints, all stemming from the initial error and misunderstanding – we address this as one point below.)

1. Inconsistencies between CFD simulations and experimental flow rates (major conceptual issue): While CFD simulations were performed at filling and voiding flow rates of 0.002 and 1.7 mL min^{-1}, respectively (line 277), the infection experiments were conducted at 0.02 and 0.7 mL min^{-1} (e.g. lines 305–306 and schematics of the P-FLO system). Since wall shear stress scales with volumetric flow rate under laminar flow conditions, the shear stresses experienced by the urothelium during infection likely differed substantially from those validated

by CFD. … This discrepancy raises several additional concerns:
• If a filling flow rate of 0.02 mL min^{-1} was indeed used experimentally, the transwells in Supplementary Fig. 6A and C would have received 7.2 mL of pooled human urine over 6 h, which is incompatible with the maximum supported volume of 1.5 mL specified by the supplier. … • If the CFD-derived filling rate of 0.002 mL min^{-1} is instead the correct experimental value, this corresponds to 0.24 mL per transwell over 2 h. In that case, the subsequent voiding step (0.7 mL min^{-1} or is it 1.7 mL min^{-1} for 1 min?) would completely empty the apical compartment, even when accounting for the initial inoculum volume (which is not specified in the Methods). … • It is unclear whether the apical compartment is fully voided or whether a dead volume remains, which seems likely given that the PDMS plug and tubing cannot contact the microtissue directly without causing injury. The authors should quantify this dead volume and discuss how it compares to clinically relevant post-void residual urine volumes (with up to ~100 mL considered normal in humans).

Overall, the mismatch between simulated and experimental flow conditions, combined with unclear apical volume dynamics, undermines the physiological interpretation of shear stress in the P-FLO system and must be clarified.

We do most sincerely apologise for our honest mistakes in the CFD simulations and are so grateful to this referee for flagging this up. These simulations have been re-run and the methodology section has been updated with the correct flow rates used in the experiments. The errors did not affect any of our original conclusions. Below is the text we added to fix this:

"Two different volumetric flow rate values were thus selected for this work, to simulate bladder filling and voiding respectively. These were estimated by manual calculation to be 0.02 mL min-1 and 0.7 mL min-1, respectively. Subsequently, a CFD simulation was run on the P-FLO model to confirm the expected shear stress profile acting over the microtissue surface area. Given the relatively low flow rate necessary to obtain wall shear stress values in the 0.017 to 17 Pa range, the flow regime was modelled as laminar (the highest Reynolds number expected in the system is estimated to be ~12)". (Line 902-907)

We acknowledge that the referee has misunderstood the set-up of P-FLO solely because we have failed to explain it in sufficient detail, for which we apologise. We are grateful that this has been flagged up so that we could amend our descriptions accordingly to benefit all readers.

A new diagram was added in **Supplementary Fig. 7A** to explain the P-FLO system, where urine flows continuously from the syringe pump into the 3D-UHU inlet, exits the model from the 3D-UHU outlet, and is then directed into a waste reservoir. The P-FLO system does not physically fill and empty the apical compartment of the Transwells to reproduce bladder priming-voiding cycles; instead, it has been developed to mimic physiologically relevant levels of shear stress acting on the urothelial wall during these cycles. The P-FLO system specifically modulates the apical flow rate over time, to mimic the temporal changes in wall shear stress levels that occur during micturition cycles. As described in the Methodology section, these controlled variations in flow generate wall shear stress levels associated with bladder filling and voiding.

The following statements were added into the results to clarify this:

"To recapitulate the mechanical forces generated by urine flow, we engineered a mesofluidic platform called the Plug-Flow Linked Organoid (P-FLO) system (**Supplementary Fig. 7A**), designed to introduce physiologically relevant levels and temporal profiles of wall shear stress into the 3D-UHU microtissue model[23,24] (and indeed to any other microtissue or planar organoid model compatible with the Transwell system). The system enables cyclic changes of flow rate (and hence of applied wall shear stress) in the apical surface of the differentiated

urothelial microtissue, mimicking the shear forces associated with the filling and voiding phases of the micturition cycle (**Supplementary Fig. 7A**). A continuous flow of 0.02 mL min^{-1} was applied for 6 h to simulate the shear forces of bladder filling. To mimic urination cycles, this flow was interrupted every 2 h by a 1-min 'voiding' event, namely, the application of an increased flow rate of 0.7 mL min^{-1}. Additionally, we tested a flow protocol without the voiding phases (in which a continuous flow of 0.02 mL min^{-1} was applied for 6 h) "(Line 337-347)

And in the Methods section as follows:

"20 mL syringes loaded with pooled urine were connected to the plugs' inlets and placed into the multi-channel syringe pump. The outlets were connected to a waste reservoir, and the full system was placed into an incubator at 37°C with 5% CO2. A low flow rate was used to mimic the shear forces of the filling phase (0.02 mL min-1) for 6 h, interrupted every 2 h with a higher flow rate (0.7 mL min-1) for 1 min to mimic the shear forces characteristic of the voiding phase i.e. micturition."(Line 930-935)

We trust that these amendments have now provided the requested clarity.

2. Limited strain diversity in infection experiments: Although the authors initially test multiple UPEC strains (including clinical isolates) in axenic culture, all infection experiments in the tissue model are performed exclusively with UTI89. Given that clinical isolates were already available, these should be included in the infection experiments to determine whether key phenotypes, such as filamentation under flow and discrepancies in treatment efficacy between static and flow conditions, are conserved across strains.

Two additional strains were added (see answer to Referee #2, query 4 for full details), which fully addresses this comment as well.

3. Limited antibiotic diversity in infection experiments: Given the broader claims regarding treatment failure under flow, inclusion of at least one additional clinically relevant antibiotic with a distinct mechanism of action would help establish whether the observed effects are nitrofurantoin-specific or represent a general property of the P-FLO system.

We chose to focus on two radically different treatments in this paper: a phage treatment and the most widely used UTI antibiotic, alongside their combination. We refer this referee to our initial response to Referee #2 point 4 about prioritization considerations. Future studies testing more antibiotics would certainly be interesting. In the meantime, we have added the paragraph below to the Discussion section, to acknowledge this as a limitation and consideration for future research:

"A limitation of this study is the restricted diversity of treatments evaluated in the infection experiments. We chose to focus specifically on two fundamentally different antimicrobial approaches: phage therapy and the most widely used antibiotic for UTI treatment, both individually and in combination. This strategy allowed us to directly compare conventional antimicrobial treatment with phage-based therapy within the same experimental framework. Nevertheless, evaluation of additional antibiotic classes in future studies would help determine whether the observed effects are generalizable"(Line 695-700)

4. Overinterpretation of persistence without long-term follow-up: The authors propose bacterial persistence as a potential explanation for antibiotic and/or phage treatment failure. However, persistence is not directly demonstrated. The experimental design focuses on short-term outcomes (≤8 h of treatment) and relies exclusively on end-point measurements, without assessing regrowth after treatment removal. Recurrent UTIs in vivo are driven by long-term persistence and relapse over days to weeks, often involving quiescent intracellular bacterial communities (IBCs). Although IBCs are observed in this model, they are not followed

longitudinally. Real-time imaging or delayed end-point measurements after antibiotic removal would substantially strengthen claims regarding persistence, filamentation, fluxing, and recurrence.

We apologise for the confusion: our use of the word "persistence" in the Introduction was referring to phenotypic antibiotic persistence, not to long term "persistence" in the sense of recurrence. We have amended the Introduction to "phenotypic tolerance and persistence" to make it clearer.

To our knowledge, we make no claims in the paper that persistence is the cause for antibiotic or phage treatment failure.

On the other hand, we stand by our claims that the generation of IBCs and filaments would support a general phenomenon of recurrence, which we do not believe is controversial based on other related research in the literature. The proposed longer-term experiments would indeed be of interest for the future. In the conclusions we have acknowledge this limitation as follow: "In addition, we focused on treatment failure during therapy administration, so while our findings demonstrate incomplete bacterial clearance under these conditions, future studies in this model should explore post-treatment regrowth dynamics[22]."(Line 700-703)

5. UP3 immunostaining performed with permeabilization: For immunofluorescence staining of UP3, the tissue was permeabilized. As UP3 is a membrane protein, permeabilization may artificially increase signal by staining cytosolic (immature) protein pools. UP3 staining should be performed without permeabilization to accurately reflect mature, membrane-localized expression.

Our methodology was previously published in Flores et al 2023 and Jafari et al 2023. Additionally, we did test the immunostaining prior to permeabilization and no differences were observed. These data do not fit the narrative of the paper, but they can be provided if the reviewer wishes to see it.

6. Use of 70% pHU for antimicrobial susceptibility testing: Although the authors demonstrate that all strains can grow in 100% pooled human urine, subsequent antimicrobial susceptibility testing is performed in 70% pHU supplemented with 30% caMHB. From a physiological perspective, 100% pHU would be the most relevant medium, as reduced growth rates and altered osmolarity or pH are known to affect antibiotic efficacy (e.g. Yang et al., Urology 2014; Mickiewicz et al., Nature Communications 2019; Fabijan et al., Bacteriology 2022; Paduthol et al., BioRxiv 2025). Additionally, MHB contains starch, which can bind toxins released upon antibiotic treatment. Even 30% caMHB may therefore buffer bacterial stress responses, potentially confounding susceptibility results. If 70% pHU is retained, this choice requires stronger justification and authors should avoid phrasing it as physiological relevant. Fig. 1B should also explicitly state "70% pHU" rather than "pHU," as the current labeling is misleading.

As requested by Referee #1, we performed a new analysis of the bacterial growth curves. The corresponding results (doubling time, maximum growth, and area under the curve) can now be found in **Supplementary Fig. 1A,** and additional explanations are provided in the response to Referee 1 (point #1).

We want to emphasise that 70% pHU was previously reported by Heithoff et al. in an influential paper as a physiologically superior method to identify if drugs will fail in later stages of clinical trials in comparison with standard growth media. Although we had mentioned this paper in the original manuscript, the following statements were added to the Discussion, to bring this point out more strongly:

“Heithoff *et al*. showed that, by more closely recapitulating the physiological conditions of infection (i.e. presence of urine, serum or cell culture media) in comparison with standard bacterial growth media [MHB]), it was possible to achieve a closer prediction of antimicrobial performance in the clinical setting.[47] In a similar manner, we showed that three UPEC isolates had an increased MIC against cefalexin when 70% pHU was used, crossing the defined EUCAST breakpoint[47]. However, most antibiotics were largely unaffected, suggesting that additional factors besides urine might lead to a lack of concordance between MIC and clinical outcomes.

Nitrofurantoin demonstrated the most potent antimicrobial activity against all the isolates tested in both standard and urine-containing media. As we employed the cationic-adjusted version of MHB (caMHB) with divalent cations at physiological concentrations, a less marked difference between 70% pHU and caMHB microenvironments compared with Heithoff and colleagues' work was expected. However, when the granularity of the assay was increased by using time-killing assays instead of the fixed endpoint MIC, the efficacy of nitrofurantoin against UPEC UTI89 was shown to be reduced in the urine microenvironment despite this strain's sensitivity in the standard caMHB-based MIC”. Line 538-552))

Even though growth at 100% pooled human urine was markedly impaired, which is not optimal for MIC, in response to the referees we also newly tested the panel of 9 strains against nitrofurantoin in 100% pHU (added to **Fig. 1B-C**).

In the Results section we added the following statements:

“The lowest MICs were reported in 100% urine, which may reflect the reduced bacterial growth. However, there were no differences in nitrofurantoin MIC between caMHB and 70% pHU.” (Line 152-154)

“In general, we again observed lower MBC levels in the 100% urine condition due to the reduced growth conditions”.(Line 167-168)

7. Claims of IBC survival after nitrofurantoin exposure lack regrowth evidence: The authors state that IBCs survive nitrofurantoin exposure and may contribute to treatment failure (line 506). However, no regrowth of tissue-associated bacteria after antibiotic removal is demonstrated. A recent microfluidic bladder model showed such regrowth directly (Paduthol et al., BioRixv 2025). The authors should either demonstrate regrowth in their own system or temper their conclusions accordingly and acknowledge prior work.

Thanks for this comment, we wanted to understand treatment failure during the duration of the therapy provided in the manuscript, as our central question was why treatment fails even when the bacteria are susceptible in in vitro settings over *the same time scale.*

We were not aware of the very nice prior work when we submitted the paper, and we've now cited this work in the manuscript (in both the Introduction and Discussion sections).

As regrowth experiments were beyond the scope of the current study, we agree with the referee's solution of tempering our conclusions, by mentioning in the Discussion that this future work would be of significant interest:

“In addition, we focused on treatment failure during therapy administration, so while our findings demonstrate incomplete bacterial clearance under these conditions, future studies in this model should explore post-treatment regrowth dynamics[22]. (Line 700-702)

8. Insufficient evidence for immunomodulatory effects of LCPR1: The claim that LCPR1 attenuates host inflammatory responses and reduces tissue damage (line 558) is based solely on IL-1β secretion. This is insufficient to support broad immunomodulatory conclusions. Additional markers, such as NF-κB activation, TNFα, interferons, or barrier integrity readouts (e.g. tight junction staining or TEER measurements), are needed.

We completely agree and this has now been addressed. See the answer to Reviewer #2, point 3 for how we have addressed the immunomodulatory issue.

As for the request for barrier integrity assessments, we have now performed TEER measurements at 12 hpi and after treatments (20 hpi). Perhaps unsurprisingly, the phenotype could not be rescued at 12h as the barrier function of the organoid is already compromised. The data can be found in **Supplementary Fig. 2** and **Fig. 2G** and the following sentence was added in the manuscript:

"However, the barrier function of the microtissue model, as measured by TEER, was already compromised after 12h of infection (**Supplementary Fig. 2B**), which could not be restored by the nitrofurantoin treatment (**Fig. 2G**)". (Line 217-219)

9. Barrier integrity under flow and reduced tissue thickness: Despite claims that flow does not alter barrier integrity (line 588), tissues exposed to 6 h of flow appear markedly thinner (Supplementary Fig. 5D) compared to static controls (Supplementary Fig. 5C). The authors should comment on this observation and assess whether repeated filling/voiding leads to epithelial exfoliation (e.g. by analyzing effluent for shed cells) or cell death (e.g. by DRAQ7 stainings).

We apologise that the image we chose was not representative of the average behaviour, which is that urothelial thickness is unaffected by flow. A more representative image was added (**Supplementary Fig. 7E**), and urothelial thickness was analysed from at least 3 biological experiments (**Supplementary Fig. 7D**), showing no difference between the two groups. Hence, exfoliation and cell death staining were not performed.

10. In addition, Supplementary Fig. 5 contains inconsistent flow-rate values (0.002 vs 0.02 mL min^{-1} for filling; 0.7 vs 1.7 mL min^{-1} for voiding), mirroring discrepancies in the main text. These must be corrected consistently across the manuscript.

We apologise for the typo, which has been corrected in the manuscript.

11. The markedly reduced TEER values in the static, uninfected condition (~500 $\Omega \cdot cm^2$) compared to the original 3D-UHU model (~1000 $\Omega \cdot cm^2$; Jafari & Rohn, 2023) also require verification and explanation. Does insertion of the PDMS plug compromise tissue integrity?

TEER values in Jafari & Rohn were performed using EVOM3 with a STX2-Plus electrode (World Precision Instruments). Those electrodes are no longer available and, for this manuscript, we used the new generation of electrodes named STX4 (see updated Methodology section). In the new electrode system, our TEER values for uninfected organoid are around 400-300 OMS which is highly reproducible, and which is sufficient to maintain urine in the apical chamber without any leakage. The data are not comparable because different electrodes and slightly different culturing conditions were used. The insertion of the PDMS plug had no effect on TEER, as we measured a similar TEER in the static 3D-UHU (see **Supplementary Fig. 7D**).

12. Filamentation under flow: The authors need to cite prior work already demonstrating UPEC filamentation under flow-induced mechanical stress (e.g. Andersen et al., Infection

and Immunity, 2012) – which limits the novelty of the presented work. It remains unclear whether the observed filaments arise from intracellular bacteria fluxing out of infected cells or represent an extracellular phenotype. Earlier and later time points, as well as live imaging, would help clarify filament formation dynamics and reversibility.

Thank you for this important point. Although our system is very different from that of the paper mentioned, we agree that this should have been mentioned, and indeed contrasting our work with theirs brings up a point of interest about kinetics. Firstly, to respond to this point, we analysed growth kinetics, adhesion and filamentation for UTI89 over the voiding events at 2, 4, and 6 h and - in parallel - in static conditions. We thank the referee, because the new data are very illustrative and strengthen the manuscript. We have updated **Fig. 3,** and amended the Results section as below:

"Time-resolved bacterial growth was monitored at t = 0, 2, 4, and 6 h under both flow (voiding/filling cycles) and static conditions. Voiding events (t = 0, 2, and 4 h) were applied exclusively under flow, with samples collected immediately post-voiding; parallel samples were obtained at matched time points under static conditions (**Fig. 3B**). Significantly lower CFU counts were recovered from the planktonic compartment at t = 2 and 4 h under flow conditions ($p = 0.0097$ and $p = 0.0017$, respectively). By 6 hpi, bacterial loads in the apical compartment were comparable between conditions. A similar trend was observed for the attached bacterial population, with significant reductions in CFUs at t = 2 h ($p < 0.0001$) and t = 4 h ($p = 0.0148$) under flow conditions, but no difference at t = 6 h ($p = 0.8396$). (**Fig. 3C**). Despite comparable bacterial loads at 6 hpi, barrier integrity was significantly better preserved under flow conditions (**Fig. 3D**), potentially reflecting reduced cumulative bacteria-epithelium contact over time (**Fig. 3B-C**). Bacterial attachment to the urothelium was reduced after the voiding-filling cycles in comparison with the static control at 2 and 4 hpi. However, at 6 hpi, similar bacterial coverage area was observed for the static vs flow conditions (**Fig. 3E-F**). These findings indicate that, despite the mechanical challenges imposed by voiding/filling-associated shear stresses, UTI89 retains the capacity to adhere to the urothelium and establish infection-associated growth over time". (Line 374-388)

Strikingly, *E. coli* UTI89 underwent marked bacterial elongation/filamentation, as observed by both confocal and electron microscopy under the filling-voiding cycles 6 hpi), which was observed only rarely in the static model (**Fig. 3F-H)**. To assess the impact of flow dynamics on bacterial morphology during infection, the cumulative fraction of bacterial objects as a function of their geodesic diameter (i.e. a measure of cell length) under static (no flow) and dynamic (flow) conditions was quantified using our 3D-bacterial segmentation image analysis pipeline named BactoMeasure3D. Cumulative distribution analysis across the 2, 4, and 6 h time points revealed consistent differences in bacterial size profiles between conditions (**Fig. 3I**). Under static conditions, size distributions were strongly left-shifted at all time points, with the cumulative fraction rapidly approaching 1.0 at relatively short geodesic diameters, indicating a predominance of smaller bacterial cells. In contrast, flow conditions produced broader distributions that were progressively right-shifted, with a more gradual increase in cumulative fraction and enrichment of larger objects. This divergence was evident as early as 2 h and became more pronounced over time, consistent with a flow-dependent increase in cell elongation. This was further evidenced by the area-under-curve (AUC) of the cumulative fraction plots (**Fig. 3I)**. Under dynamic conditions, AUC values gradually increased over time from 5.83 to 13.1 for 2 h and 6 h, respectively, whilst under static conditions AUC changed from 6.67 to 6.43; a statistical difference between static and dynamic was only observed 6 hpi ($p = 0.0399$). In the HSS and ISS regions after voiding-filling cycles, the extent of bacterial cell elongation was comparable (**Supplementary Fig. 8D**). To assess whether the observed bacterial elongation was a consequence of the alternating filling-voiding cycles (and the associated temporal change in wall shear stress levels), we tested a flow protocol without the voiding phases. The filling phase alone (6 hpi) induced cell elongation as observed in the confocal images (**Supplementary Fig. 8B-D),** suggesting that the low wall shear stress

regimen representative of bladder filling was sufficient to induce bacterial elongation and that alternation of low and high wall shear stress levels was not the primary mechanism. These findings suggest that flow-induced filamentation represents an adaptive response to the bladder mechanical environment that may enhance bacterial attachment and surface colonization under hydrodynamic stress. Filamentous UPEC morphologies have also previously been associated with reduced susceptibility to phagocytic clearance, supporting the concept that morphological plasticity constitutes a mechanism by which bacterial pathogens can subvert host immunity[64].(Line 390-418)

This has now been contextualised in the Discussion as follows:

"In previous 2D bladder flow culture models, UPEC filamentation was observed only during later stages of infection, following prolonged intracellular colonisation, gentamicin-mediated elimination of extracellular bacteria and subsequent bacterial escape ('fluxing') from urothelial reservoirs during secondary surface colonisation under urine flow[88]. Our findings suggest that hydrodynamic shear stress and urothelial surface-associated microenvironmental cues may directly induce early filamentation independently of the classical intracellular infection cascade, since UPEC filaments were observed at early infection time points (2-6h)".(Line 649-655)

13. Discrepancies between representative images and quantitative coverage data: In Fig. 4, images under flow conditions (4B, 4E) appear to show near-complete surface coverage, whereas quantitative analysis reports ~22% coverage (Fig. 4J). A similar discrepancy exists for static infection (Fig. 2E vs 2J). The authors should ensure that representative images accurately reflect the quantified data or verify their Fiji analysis with manual validation.

We apologise for the lack of concordance quite rightly noted here. **Fig. 4** has been updated following comments from another reviewer (Referee #1, point 6) and more representative images to show bacterial coverage and IBCs under flow have been selected.

In the case of coverage area, as stated in the methodology, the 40x magnification was used to cover a larger microtissue area: "Images acquired at 40× magnification were used to quantify biofilm coverage area." To clarify this, we have added representative images for each condition acquired at 40x, for both static and flow, which can be found in **Supplementary Fig. 2A** and **Supplementary Fig. 10B.**

Additionally, the following statements were added to the Results section:

"this finding was consistent with reductions observed in the confocal images (**Fig. 2D**) and the subsequent bacterial coverage area analysis (**Fig. 2E** and **Supplementary Fig. 2A**) using images acquired at 40x magnification, enabling analysis of a larger microtissue area and providing a more representative assessment of bacterial distribution" (Line 212-215)

"Image analysis at 20 hpi (**Supplementary Fig. 2A and 10B**) at 40x magnification demonstrated that flow conditions markedly increased bacterial attachment to the urothelium, with 22% coverage under flow compared with 10% under static conditions (**Fig. 4C)**, despite comparable urothelium-associated CFU counts between the two conditions."(Line 465-469)

Higher-magnification images, particularly at 60X, may be less representative because the samples are highly heterogeneous. The reported images were selected to highlight regions where bacterial attachment was observed. We have clarified this point in the revised manuscript. We have removed the word 'representative' in the **Fig 2**. legend and clarified as follows in the legend of Fig. 2: "3D rendering of confocal microscope images of *E. coli* UTI89-GFP untreated, exposed to nitrofurantoin, LCPR1 and dual treatment in 3D-UHU. Imaging

was focused on regions with bacterial attachment due to sample heterogeneity." (Line 1127-1129)

14. Viability of adherent bacteria inferred solely from CFU counts: The conclusion that adherent bacteria under flow are non-viable after treatment is based exclusively on CFU enumeration. Given the mechanical and antibiotic stresses involved, bacteria may enter a transiently non-culturable state by standard plating techniques. Additional viability or metabolic assays (e.g. live/dead staining, resazurin assays) and recovery experiments after treatment removal are needed to support claims regarding bacterial viability.

Under CFU analysis, we observed that bacteria under flow are even more viable as higher CFUs were recovered in the planktonic side under flow in comparison with static. In the case of attached/intracellular populations, we observed similar patters between flow and static (**Supplementary Fig 10C-D**).

But to address this comment, in the 6h infection under flow, we evaluated metabolic activity using a resazurin assay of the planktonic population (**Fig. 3J**). In the case of UTI89, even though CFUs were not statistically different in both conditions, we observed that the planktonic cells were less metabolically active in flow conditions; this could potentially explain the lack of antimicrobial activity.

The following statement has been added to the Discussion section, to elaborate on this interesting point:

"In contrast to the urothelium-associated population, antimicrobial activity against planktonic bacteria was diminished under flow. This effect may reflect the reduced metabolic activity of UTI89 observed in the P-FLO/3D-UHU model at 6 hpi in comparison with the static model, which could contribute to decreased bacterial susceptibility to antimicrobial treatment."(Line 673-676)

• Lines 77-83: Claims regarding poor translatability of existing UTI models should be softened, as the current model also relies on a single immortalized cell line and usage of a laboratory-adapted UPEC strain only. Additional UTI models should be cited (e.g. Sharma et al., 2021; Horsley et al., 2018; Paduthol et al., 2025).

Thank you for this important point; we did not mean to overstate this nor to leave out important context. The Introduction has now been updated and references and examples have been added, as follows:

"Existing experimental models of UTI (ranging from cell culture to animal infection systems) have provided important mechanistic insights into UPEC behaviour but exhibit significant translational limitations. Murine models, for example, differ from humans in terms of bladder physiology, immune response and urine composition[18]. Furthermore, pharmacokinetic and pharmacodynamic parameters of antibiotics often translate poorly between mice and humans, hindering efforts to define optimal treatment regimens[19].

Recent tissue-engineering advances have provided human-relevant models to study UTI pathology. For example, a donor-derived human bladder organoid model has been developed to investigate UPEC and bacteriophage interaction at the molecular level[20]. To modulate stretching patterns during micturition and immune cell infiltration, a bladder-on-chip model was established to investigate intracellular reservoirs[21]. Advanced mini-bladder microphysiological systems incorporating stratified urothelium, urine perfusion and micturition-like dynamics have further demonstrated how urine composition can reduce fosfomycin efficacy and promote persistent cell wall-deficient UPEC populations[22]. We recently developed the static 3-dimensional urine-tolerant human urothelial microtissue Transwell model (3D-UHU), which

allows investigation of UTI in a fully stratified and differentiated urothelium in a 100% human urine apical environment[23,24]. Together, these innovations and others are driving a new era of personalized, mechanism-based approaches to investigate UTI and antimicrobial therapies" (Line 81-99).

In the interest of space, we did not cite our own earlier paper (Horsley et al.) as the 3D-UHU model is an advanced version of the same concept.

We take the referee's point about the limitations of our model being derived from one cell source, which we have now added to the Discussion:

"Additional improvements to our model could include the use of donor-derived stem cells instead of the commercially available HBLAK cells, which would allow assessment across different host backgrounds and the use of autologous immune cells".(Line 703-705)

• Line 36: Clarify that LCPR1 urine experiments refer to axenic cultures

We feel the word 'axenic' is rather unfamiliar to most. Hence, we have instead simply described the assay for a more general audience in the Methodology section:

"Additionally, serial dilutions were performed with PBS in 96-well plates and plated on LB agar plates for CFU enumeration to quantify bacterial killing and phage recovery assay (as describe above) to quantify phage infectivity."(Line 874-876)

.

• Lines 85, 109, 858–859: The term "organoid" is misleading for a transwell-based models, even if for other studies those were derived from dissociated spheroids. The system presented should be consistently described as a transwell-based bladder model. This also applies for the nomenclature of the Plug-Flow Linked Organoid (P-FLO) system; it should be named Plug-Flow Linked Transwell system or Plug-Flow Linked 3D-UHU system.

We have interacted with many referees over the years about the personal nomenclatural choice of whether an organoid can be planar as well as spheroid shaped – some agree, others do not. There are certainly examples of planar organoids (including air-liquid interface cultures) where the authors of the papers specifically refer to them as organoids – just a few recent examples:

Planar skin organoids: doi: 10.3791/69088.
Planar neural organoids: doi: 10.1007/s11060-026-05515-5
Planar gut organoids: doi: 10.1038/s41378-023-00615-y. eCollection 2023

However, to avoid any confusion, we do usually nowadays use the word "microtissue" to refer to 3D-UHU, and we note that the word "organoid" indeed still crept occasionally into our original paper when we were referring to 3D-UHU. These have been fixed throughout. However, we stand by our assessment that planar organoids can be grown in Transwells, and therefore our P-FLO system, so we will keep this flow-evocative name, especially as it is already in use after our preprint was published. We also clarified this point by adding a parenthetical observation to the final sentence of this text:

"To recapitulate the mechanical forces generated by urine flow, we engineered a mesofluidic platform called the Plug-Flow Linked Organoid (P-FLO) system (**Supplementary Fig. 7A**), designed to introduce physiologically relevant levels and temporal profiles of wall shear stress into the 3D-UHU microtissue model[23,24] (and indeed to any other microtissue or planar organoid model compatible with the Transwell system)."(Line 337-341)

In the Discussion, we also mentioned the utility to both microtissues and organoids:

"For the current study, we designed a relatively inexpensive mesofluidic system called P-FLO to incorporate controlled flow and cyclical shear stress associated with bladder filling and voiding onto the apical side of commercially available 12-well plate Transwells, which is compatible with the differentiated urothelial 3D-UHU model and many other organoid and microtissue systems."(Line 619-622)

• Line 91: Remove the term "personalized," as no patient-derived tissues are used.

We reproduce the line from the Introduction here (italicized emphasis added): "Together, these innovations *and others* are driving a new era of personalized, mechanism-based approaches to investigate UTI and antimicrobial therapies." This is a highly generalised statement about the promising and bright future of this field, where patient-derived donor cells are indeed being employed among many other innovations. Indeed, the first sentence of this paragraph highlights Zulk et al. as using donor-derived cells. As a clearly future-pointing sentence that includes multiple papers in the paragraph as well as others not cited, we cannot see how this could possibly be interpreted as being applicable to the manuscript itself.

• Lines 94-97: Statements regarding treatment failure across pathogens require citations.

Two recent review articles have now been cited (Simoni et al 2025 and Garcia-Maset et al 2025).

• Line 225: Change "in LB agar plates" to "on LB agar plates."

We apologise for the typo, which we have corrected.

• Line 229: Avoid the term "biofilm-like" without evidence of biofilm structure.

Thanks for the suggestion, we have removed the "biofilm-like" term.

• Fig. 2 timeline discrepancy (12 h vs 14 h) should be resolved.

Apologies and thanks for catching this typo: this is now changed to 12 h.

• Lines 515, 525, 715, 722, 729, 759, 774, 779: Various overinterpretations, missing citations, and terminology issues should be corrected as detailed in the upper comments.

- 515: Despite this confirmed in vitro drug-sensitivity, treatment at concentrations above the MIC (7xMIC) failed to reduce the bacterial burden in the urothelial compartment, exemplifying the discrepancy between simplified in vitro tests and platforms that mimic the host microenvironment and host-pathogen interactions.

We weren't entirely sure what the referee did not like about this, and presume they wanted a citation for the general phrase at the end, which we have now added in line 233.

- 525: In contrast, LCPR1 efficacy was markedly reduced in 70% pHU:30% MHB, with no significant growth inhibition observed at MOI 1 and 10. Only at MOI 100 partial growth inhibition was observed

The manuscript has been updated: "The bacterial growth was impaired up to 10 h for all MOIs tested in MHB. After this period, partial regrowth was observed (OD_{600} ~0.5), while the untreated control reached full growth (OD_{600} ~2) (**Supplementary Fig. 3A**). In contrast, in 70% pHU:30% MHB, the UTI89 growth curve in the presence of LCPR1 (MOI = 1 and 10) exhibited a similar growth pattern in comparison with the untreated control. Only at MOI 100, a more pronounced effect in growth was observed (**Supplementary Fig. 3B**). At MOI 100, LCPR1

decreased UTI89 growth in both media versus the untreated UTI89 control, but the area under the curve (AUC) remained higher in 70% pHU:30% MHB than in standard medium, consistent with reduced phage efficacy in urine (**Supplementary Fig. 3C**). Despite differences in initial killing efficacy, after 24 h, LCPR1 at MOI 100 reduced bacterial CFU by ~4 log in MHB and ~3 log in 70% pHU:30% MHB pHU (**Supplementary Fig. 3D**). Phage quantification following the killing assay demonstrated varying amplification levels after 24 h for each *E. coli* phage (Leic_001, JK03, and UP17). A reduction of phage titres (uninfected control with LCPR1 and media alone) in 70% pHU:30%MHB titre was also observed for Leic001 ($p < 0.0001$) and UP17 ($p = 0.0135$) in comparison with MHB, suggesting that phages were less stable in the urine microenvironment media (**Supplementary Fig. 3E-G**). No differences in phage titre were observed for JK03 in both media. In the UTI89 groups co-infected with LCPR1, the titre of Leic_001 was higher in 70% pHU:30%MHB than in MHB ($p < 0.0001$), whereas this trend was not observed for the other two *E. coli* phages (**Supplementary Fig. 3E**). The titres of JK03 and UP17 were comparable between the two media (**Supplementary Fig. 3F-G**). These findings underscore the influence of the type of culture medium on phage activity, suggesting that host-like conditions may limit therapeutic efficacy compared with standard laboratory media, as previously reported by others[46,47]." (Line 247-267)

- 715: namely intracellular reservoirs and bacterial biofilms on the urothelial surface, but it could also be compounded by additional factors such as phenotypic tolerance and persistence.

This statement has been removed from the discussion and replaced with a new sentence: "Strikingly, IBCs persisted following nitrofurantoin treatment despite an overall reduction in bacterial surface coverage, supporting the idea that protected urothelial niches can contribute to bacterial survival despite the presence of effective antibiotics in the urine".

- 722: In the 3D-UHU model, LCPR1 exhibited an attenuated host cytotoxicity and a reduced pro-inflammatory response compared with untreated infections, while bacterial burden was not reduced.

As requested by this referee's point 8, and Reviewer #2's point 3, we have included new data and amended the statement here mentioned. See the previous answer for more information on this point.

- 729: Transcriptomic analysis of phage-epithelial infections has revealed upregulation of anti-inflammatory mediators such as IL1R2, alongside repression of key pro inflammatory signalling pathways including NF-κB, TNF, TLR, and JAK-STAT57, supporting our observations.

This statement has been removed from the Discussion because of substantial changes being made to the host response results.

- 759: Introducing flow into model systems is essential to more faithfully reproduce the biophysical and biochemical cues that shape host-pathogen interactions in vivo. In static conditions, the absence of wall shear stress and nutrient gradients fails to capture key aspects of the infection microenvironment

We have added a citation to support this general statement (doi: 10.3390/bios14050225).

- 774: Although filamentation occurred in static conditions, 6 h of flow increased the frequency of UTI89 elongation in groups that were exposed to both filling/voiding cycles (with alternating low and high wall shear stress levels) or to filling only. Even at the lowest

wall shear stress tested, bacterial cell elongation frequency increased in comparison with static conditions.

As requested by point 12, see the corresponding changes.

- 779: Apart from influencing bacterial morphology, shear stress did not noticeably alter the UPEC invasion mechanism as previously observed in mice68, shed patient cells69, human biopsies70 and in the 3D-UHU static infection model (here and in Flores et al.,22).

Given our new data about IBCs, these statements have now been modified to:

"The 3D-UHU microtissue model supports the formation of both intracellular bacterial communities (IBC) and surface-associated bacterial clusters and biofilms, both of which represent key pathogenic adaptations employed by UPEC during infection[23]. UPEC invasion has previously been observed many times in mice (e.g.[68]), shed patient cells (e.g.[69]), human bladder cell lines (e.g.[70]), and human urothelial biopsies (e.g.[71]). In 3D-UHU, while nitrofurantoin significantly reduced bacterial burden, it did not cause complete eradication for the duration of our experiments even at a concentration achieved with regular clinical dosing. Strikingly, IBCs persisted following nitrofurantoin treatment despite an overall reduction in bacterial surface coverage, supporting the idea that protected urothelial niches can contribute to bacterial survival despite the presence of effective antibiotics in the urine." (Line 564-572)

• Supplementary Fig.s: Correct typographical errors (ISS vs LSS; PFU vs PFC):

ISS and LSS are actually correct, intermediate and low shear stress regions; labels are in the **Supplementary Fig. 7B** and in the corresponding legend. PFU has been corrected to PFC in the **Supplementary Fig.v3, 5** and **10;** we apologise for the typo.

Thank you for the opportunity to further revise our paper, which has improved it. The numbering system below refers to Referee 3's original list. We have deleted those items where the referee was happy and did not want more changes.

1. The authors have substantially clarified the discrepancies between CFD-simulated and experimentally applied flow conditions and corrected the incorrect flow values throughout the manuscript. The clarification that the P-FLO system represents a continuously perfused flow-through system designed to modulate wall shear stress, rather than physically filling and emptying the apical compartment, resolves several of my original concerns regarding Transwell volume capacity and complete voiding.

However, I still believe the manuscript somewhat overstates the physiological mimicry of bladder filling and voiding. The current system appears to reproduce temporal changes in shear stress rather than the broader mechanical and volumetric dynamics of true bladder filling/micturition cycles (e.g. stretch, pressure changes, urine accumulation, etc.). I would therefore encourage the authors to slightly moderate this terminology throughout the manuscript for conceptual precision.

Additionally, further clarification regarding the broad range of shear stress values reported (0.017–17 Pa; with 17 Pa more closely reflecting vascular-level shear stress than values typically associated with the bladder) together with clarification of the effective apical chamber volume and potential dead-volume/reservoir effects within the plug/tubing system would further strengthen the technical characterization of the platform.

Thank you for these comments, with which we agree.

To address the first point on overstating the physiological mimicry, we have gone through the entire manuscript and changed all overstated text (highlighted in the revised manuscript). We have also added a few sentences to emphasize that our system approximates the physiological conditions without 100% matching them. These include:

In the Results, after explaining exactly what the system does, we have added: "For brevity, we will hereafter refer to these P-FLO conditions as "filling" and "voiding" cycles" This removes the need to re-explain the limitations every time we refer to the conditions afterwards, which would become repetitive. Current lines 346-347

In the Discussion (new phrase in bold face): "Our findings demonstrate that more physiological flow conditions strongly influence UPEC infection dynamics and antimicrobial susceptibility, emphasizing the importance of incorporating hydrodynamic forces into preclinical infection models – **while acknowledging that P-FLO does lack other important cues, such as broader mechanical and volumetric dynamics of true bladder filling/micturition cycles including urothelial stretching, pressure changes and urine accumulation."** Current lines 695-700

To address the second point in this feedback; with apologies, there was a typo in the expected wall shear stress for the bladder model, and the high limit was always meant to be 1.7 Pa instead of 17 Pa (consistently with the simulation results already illustrated in the manuscript). The rest of the paragraph agrees with the now corrected value, and we thank the reviewer for catching this oversight.

The reviewer also makes a point about dead-volume/reservoir effects. Based on the simulation results, there appears to be very limited stagnant regions (i.e. dead-volume) in the well, and we wouldn't expect stagnation to occur in the connection tubing upon continuous

flow. Accordingly, the apical volume in the device and the prefilling protocol used to avoid headspace air bubbles are now clearly stated: “Before starting the experiment, both the apical and basolateral sections of the P-FLO and the tubing were prefilled with urine to avoid entrapment of air bubbles in the system.” Current lines 932-933.

2. The inclusion of additional clinical isolates substantially strengthens the manuscript and addresses my original concern regarding strain specificity of the observed flow-associated phenotypes. In particular, the observation that the pathogenic isolate EC3 displays filamentation under flow conditions whereas the asymptomatic isolate COM2 does not is informative.
However, while the authors now demonstrate broader applicability of the infection phenotypes, the generalizability of the reported treatment-response differences between static and flow conditions across diverse UPEC strains remains less explored and could be acknowledged as a limitation.

Thank you for this comment; we agree that acknowledging this limitation would clarify matters. Accordingly, we have added the following line to the Discussion:

“Future studies with more diverse strains of *E. coli,* alongside other species of uropathogenic bacteria, would be of interest to determine how universal such effects are.” (current lines 700-701).

5. The authors have now performed additional analyses of bacterial growth kinetics, re-run MIC assays in 100% pooled human urine (pHU), and more clearly explained their rationale for using 70% pHU supplemented with caMHB. However, their statement in lines 152–154 and 167–168 regarding the MICs observed in 100% pHU could still be misleading, as they state that the lower MICs observed in 100% pHU “may reflect the reduced bacterial growth”. While strongly reduced growth in pHU may indeed make MIC determination technically more challenging (e.g. distinguishing growth from no-growth conditions), the current phrasing could be misinterpreted as implying that reduced growth directly causes lower MICs. Mechanistically, slow or impaired bacterial growth often renders bacteria less susceptible to many antibiotic classes, which would instead be expected to increase MIC values due to phenotypic tolerance. I would therefore advise the authors to revisit the wording of this sentence to avoid misleading non-expert readers.

We apologise for our lack of clarity in these two separate sentences. We have reworded them as below:

“The lowest MICs were reported in 100% urine, which may reflect technical difficulties in visualising endpoints when bacterial growth is not robust, as is the case under this condition.” (lines 151-153)

“In general, we again observed lower MBC levels in the 100% urine condition similar to what occurred with the MICs.” (lines 167-168)

7. The authors explain the difference in TEER values between the presented data and their initial 3D-UHU model from 2023 by the use of different electrodes, which is a very reasonable explanation. However, they now also mention slightly different culture conditions between the two set-ups (the Jafari paper and the current manuscript). Could the authors

comment on why they modified (e.g. regarding seeding density and timing of pHU addition, as far as I can see)?Additionally, is the passage number mentioned in the Methods section inclusive of the passages performed by the supplier? If so, the authors should more accurately reflect this in the manuscript by correcting the stated passage number, as was done in the original 3D-UHU publication.

We find with our microtissue work that protocols are living documents that are constantly being tweaked. This modified protocol is the one described in our later paper Flores et al., 2023 and this was already cited in manuscript, not Jafari and Rohn, 2023. So we think that this is clear. We have added the citation in a second place in this paragraph for maximum clarity (current line 837).

Regarding the passage number, this is an important suggestion for clarity, thank you. We have inserted the parenthetical description in this line:

"Briefly, between passages 9 and 13 (where the passage number refers to the passage after thawing factory vials from the manufacturer), HBLAK cells at 80–90% confluency…" (current line 845-847)

Additionally, I have a minor readability suggestion: in several revised figures (e.g. Fig. 4C, 4D, and 4G), the order of presentation switches from static->flow to flow->static, whereas other panels (e.g. Fig. 4F) retain the original ordering of static->flow. Although the labels are correct, maintaining a consistent condition order throughout the manuscript figures would improve readability and facilitate comparison across datasets.

Thank you for this comment – we agree, and we have changed Figure 4 data presentation to follow static > flow for consistency.
